# Ligand binding: evaluating the contribution of the water molecules network using the Fragment Molecular Orbital method

**DOI:** 10.1007/s10822-021-00416-3

**Published:** 2021-08-30

**Authors:** Iva Lukac, Paul G. Wyatt, Ian H. Gilbert, Fabio Zuccotto

**Affiliations:** Drug Discovery Unit, Wellcome Centre for Anti-Infectives Research, Division of Biological Chemistry and Drug Discovery, University of Dundee, Dow Street, Dundee, DD1 5EH UK

**Keywords:** Fragment molecular orbital, Ligand binding, Water network, Quantum mechanics, Ligand optimisation

## Abstract

Water molecules play a crucial role in protein–ligand binding, and many tools exist that aim to predict the position and relative energies of these important, but challenging participants of biomolecular recognition. The available tools are, in general, capable of predicting the location of water molecules. However, predicting the effects of their displacement is still very challenging. In this work, a linear-scaling quantum mechanics-based approach was used to assess water network energetics and the changes in network stability upon ligand structural modifications. This approach offers a valuable way to improve understanding of SAR data and help guide compound design.

**Supplementary Information:**

The online version contains supplementary material available at 10.1007/s10822-021-00416-3.

## Introduction

When considering interactions of ligands with their protein targets, the contribution of solvent is often ignored or underestimated. During the binding event both ligand and protein undergo a desolvation step to allow their interaction. The reorganisation of the water molecules surrounding the ligand after the binding event contributes to the change in total binding free energy. In medicinal chemistry programs the focus is often on the energetically unstable water molecules, and targeting “unhappy” waters within the binding site is now a common drug design strategy to achieve potency gain or selectivity [1, 2] . However, often the gains or losses in affinity upon displacement or replacement of water molecules cannot be explained by considering protein–ligand interactions alone and the contribution of an extended water molecule network to ligand binding should be considered. Understanding the nature of the water molecule network around the ligand (and not exclusively water molecules in direct contact with the ligand) can help identify ligand structural modifications that could further stabilise such a network and thus be of practical use for compound design. Multiple in silico approaches [3–5] exist that aim to predict the location and energetics of water molecules in the target binding site. In general, they are successful in predicting the accurate position of most crystallographically observed water molecules. However, the calculated energy of the water molecules varies significantly between different methods, resulting in inconsistent classification of the water molecules that should be targeted.

An accurate assessment of the solvent contribution is however extremely challenging. Protein–ligand binding affinities are driven by the balance between multiple factors, many of which can only partially be accounted for by force field-based methods. On the contrary, the first principle nature of quantum mechanics (QM) calculations [6–12] , enables systematic improvements to the accuracy by which biomolecular recognition is described. QM calculations can overcome many of the limitations imposed by molecular mechanics-based approaches (e.g. polarisations and charge fluctuation). Despite the greater accuracy, QM methods have not been routinely used in drug discovery due to the high computational costs required to deal with large biological systems. Recently, the development of fragment molecular orbital (FMO) methods have increased the computational speed of QM approaches. They achieve this by fragmenting the system into smaller parts, and deriving QM based pairwise interaction energies (PIE) between the fragment and the ligand [3, 13]. By combining the PIE of all the fragment-ligand pairs it is possible to derive the total interaction energy of a ligand with its biological target.

In this work, a FMO approach has been applied to calculate the energy of single water molecules in the binding site and investigate the stability and contribution to the interaction energy of the water molecule network surrounding the ligand; this is exemplified in a set of nine Bromodomain and nine Bruton’s Tyrosine Kinase (BTK) structures. The same structures have previously been used for evaluation of five different commercial water prediction programs (3D-RISM, [14, 15] SZMAP, [16] WaterFLAP, [17] WaterRank, [18, 19] and WaterMap [20]). [3, 13] Our results show that by combining QM-FMO derived protein–ligand interaction energies together with the interaction energies for the water molecules it is possible to gain a more accurate description of the ligand binding event and estimate the contribution of the water network. This information can be very valuable to improve understanding of Structure Activity Relationship (SAR) data and help guide compound design.

## Methods

All crystal structures were processed with Schrödinger’s protein preparation wizard [21] to provide starting points for further calculations: hydrogen atoms were added after deleting any original ones, followed by adjustment of bond orders for amino acid residues and the ligand. Hydrogen positions were sampled using Schrodinger’s H-bond assignment/sample water orientation component, and the resulting networks were evaluated using the FMO method. In case of hydrogen placement ambiguity (as in the case of compound **10** in BTK for example), both solutions were evaluated.

Bond orders for amino acid residues and the ligands were adjusted and the protonation and tautomeric states of Asp, Glu, Arg, Lys, His and ligands were optimised using the protein wizard default settings (pH of 7.0 ± 1), followed by hydrogen bond network and water orientation sampling. PIE values tend to overestimate the charge-charge interactions, especially when the calculations are performed in vacuo [22]. Some of the analysed BTK ligands would be protonated at physiological pH. The protonated groups are solvent exposed, and due to desolvation effects the charge-charge interaction with the protein would be limited. Therefore, to avoid over-estimation of the charge-charge interactions we decided to follow the procedure suggested by Heifetz et al. [12] and neutralise the charged groups.

To derive the optimal starting point for the FMO calculation, the structures were subjected to a restrained minimisation procedure with the OPLS3e force field, as implemented in the minimisation protocol of the protein preparation wizard of the Schrödinger’s suite of software, where each heavy atom was allowed to deviate by up to 0.5 Å from its original position in crystal structure or model. Residues within 5 Å from the ligand atoms were included in the FMO calculations. The C-terminal carboxylic acid was capped with N-methylamine, and the N-terminal position acetylated while maintaining the geometry of the neighbouring residues. Some residues were removed/added depending on the local substructure to ensure minimal disruption in the backbone chain (see Supplementary Information). Fragmentation was done using Facio, [23, 24] according to a well-established fragmentation strategy, where each FMO fragment is defined by the side chain, the C*α* and backbone NH of a given aminoacid plus the carbonyl group of the adjacent aminoacid [10, 25] . The calculations were performed at MP2/6-31G* theory level, using GAMESS implementation [26, 27] . The analysis of the results was performed using in-house tools developed in Python 3 [28].

## Results and discussion

In this work, we studied a series of bromodomains (BRD9, BRD4 and TAF1) (Table 1). A network of five highly conserved water molecules forming a water network at the protein–ligand interface was previously identified across all these bromodomain structures [13].

**Table 1:** Bromodomain structures

Compound	Protein	PDB code [3]	pIC_50_ [13]	Ligand PIE (kcal/mol)	Water network PIE (kcal/mol)	Water PIE median
**1**	BRD9	5I40	4.88	− 70.7	− 211.6	− 41.8
**2**	BRD9	5I7X	6.63	− 89.6	− 221.1	− 41.6
**4**	BRD9	5I7Y	6.79	− 95.6	− 216.4	− 40.0
**5**	BRD9	6BQA	5.85	− 95.7	− 217.4	− 40.0
**2**	BRD4(1)	5I80	7.03	− 80.7	− 217.6	− 41.0
**4**	BRD4(1)	5I88	6.33	− 98.0	− 134.6	− 36.1
**2**	TAF1(2)	5I29	7.22	− 118.5	− 172.8	− 36.2
**4**	TAF1(2)	6BQD	6.38	− 127.7	− 108.1	− 32.4
**5**	TAF1(2)	5I1Q	7.33	− 128.5	− 142.7	− 37.6

“Water network PIE” is the total of all the water energies, whereas “Water PIE median” is the median value over the individual water molecules. Median was used as it gives a more appropriate idea of the data distribution

**Fig. 1:**
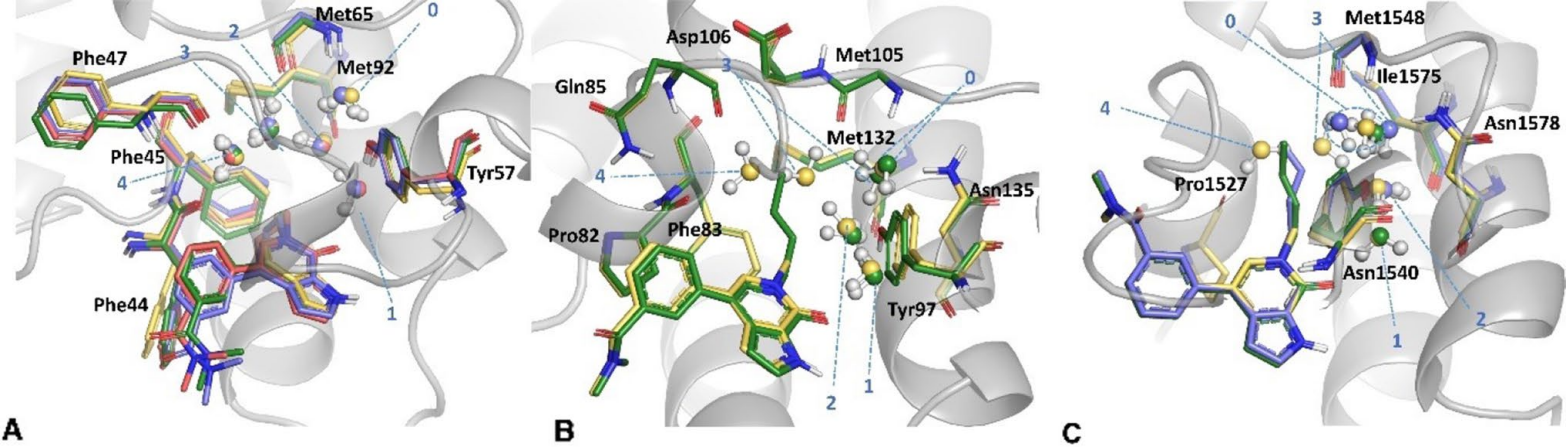
**A** Structures of four BRD9 complexes studied. **1** is shown in yellow sticks, **2** in green, **4** in blue, and **5** in salmon. **B** Structures of two BRD4 complexes studied. **2** is shown in yellow sticks, **4** in green. **C** Structures of three TAF1 complexes studied. **2** is shown in yellow sticks, **4** in green, and **5** in slate

For BRD9, the bromodomains are co-crystallised with a series of analogues, starting with a weakly potent fragment, compound **1** (compound numbering consistent with Nittinger et al. [3]), and three more complex analogues (compounds **2**, **4** and **5**). An overlay of the four BRD9 structures (5I40, 5I7Z, 5I7Y, 6BQA) shows that the growth of the ligand, during optimisation, leaves the water network intact (Fig. 1A); therefore the energies of the individual water molecules, as well as the whole water network should remain similar.

**Fig. 2:**
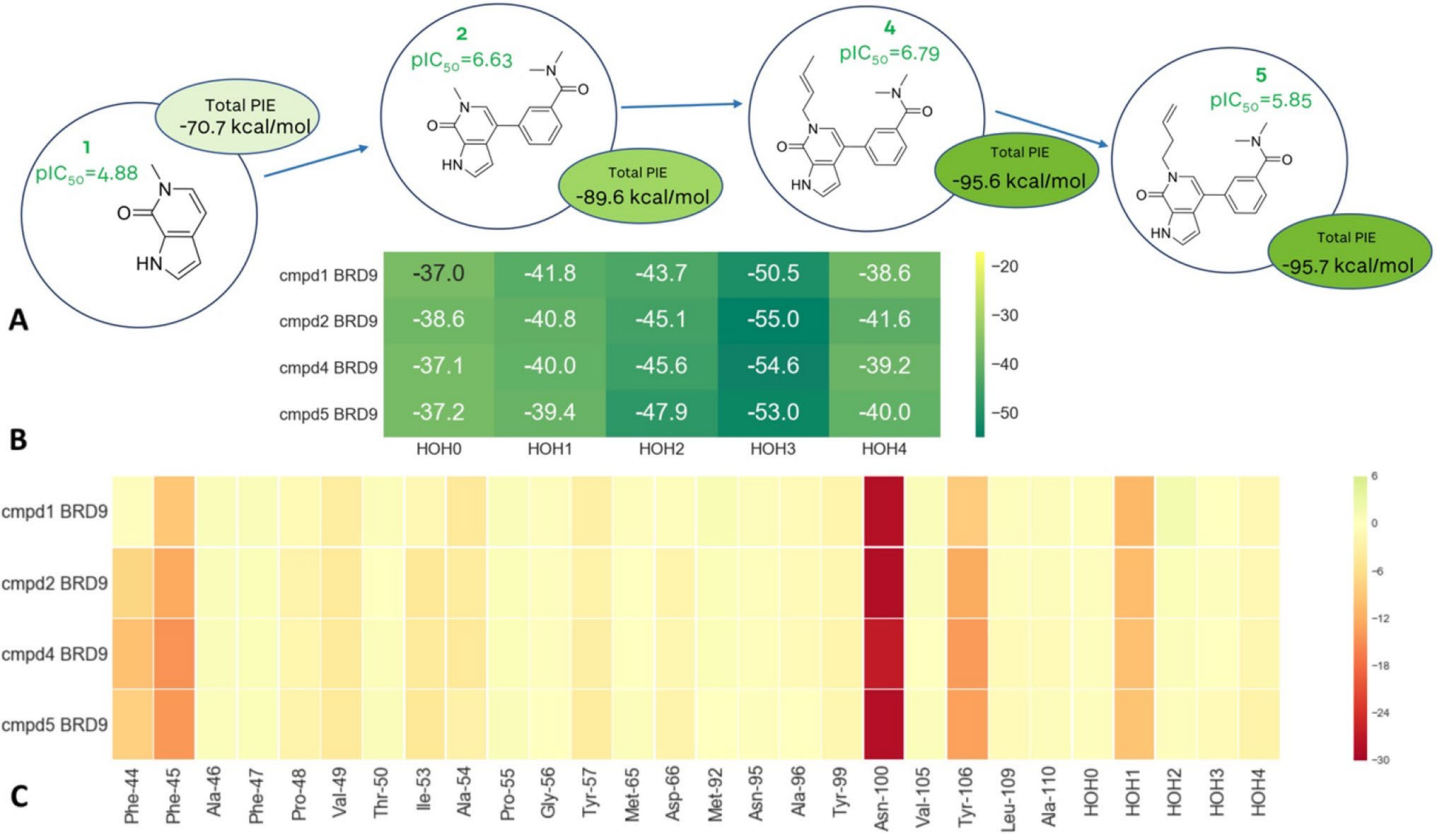
FMO results for the BRD9 in complex with 1, 2, 4 and 5. **A** Structures of four BRD9 complexes studied.** B** Energetic contributions of individual water molecules to the stability of the overall water network. **C** FMO-PIE generated contribution to ligand binding energy in kcal/mol per fragment residues. Red indicates favourable interaction energies, whereas green indicates unfavourable interaction energies. The sum of these interactions is shown in Table 1 as* Ligand PIE*

The observed differences in potency are therefore caused mainly by the differences in the protein–ligand interactions, rather than changes in the water network. This is accurately reproduced by the FMO calculations: calculated PIE correlates with the observed SAR, where the pIC_50_ increases going from **1** to **2** to **4** (Fig. 2). Compound **5** was calculated to have the same interaction energy as **4**, despite almost one log unit difference in the measured pIC_50_. It is worth remembering that a FMO calculation is an estimate of the enthalpic component of the ligand binding and it is possible that the higher entropic penalty associated with the higher flexibility of the 1-butenyl side chain in **5**, that goes into the deeper part of the pocket, is contributing to the observed discrepancy in potency. The calculated energies of water networks and their medians are comparable amongst the four structures. There is very little discrepancy in energy profiles suggesting that any changes in potency are not caused by potential changes in the water network.

**Fig. 3:**
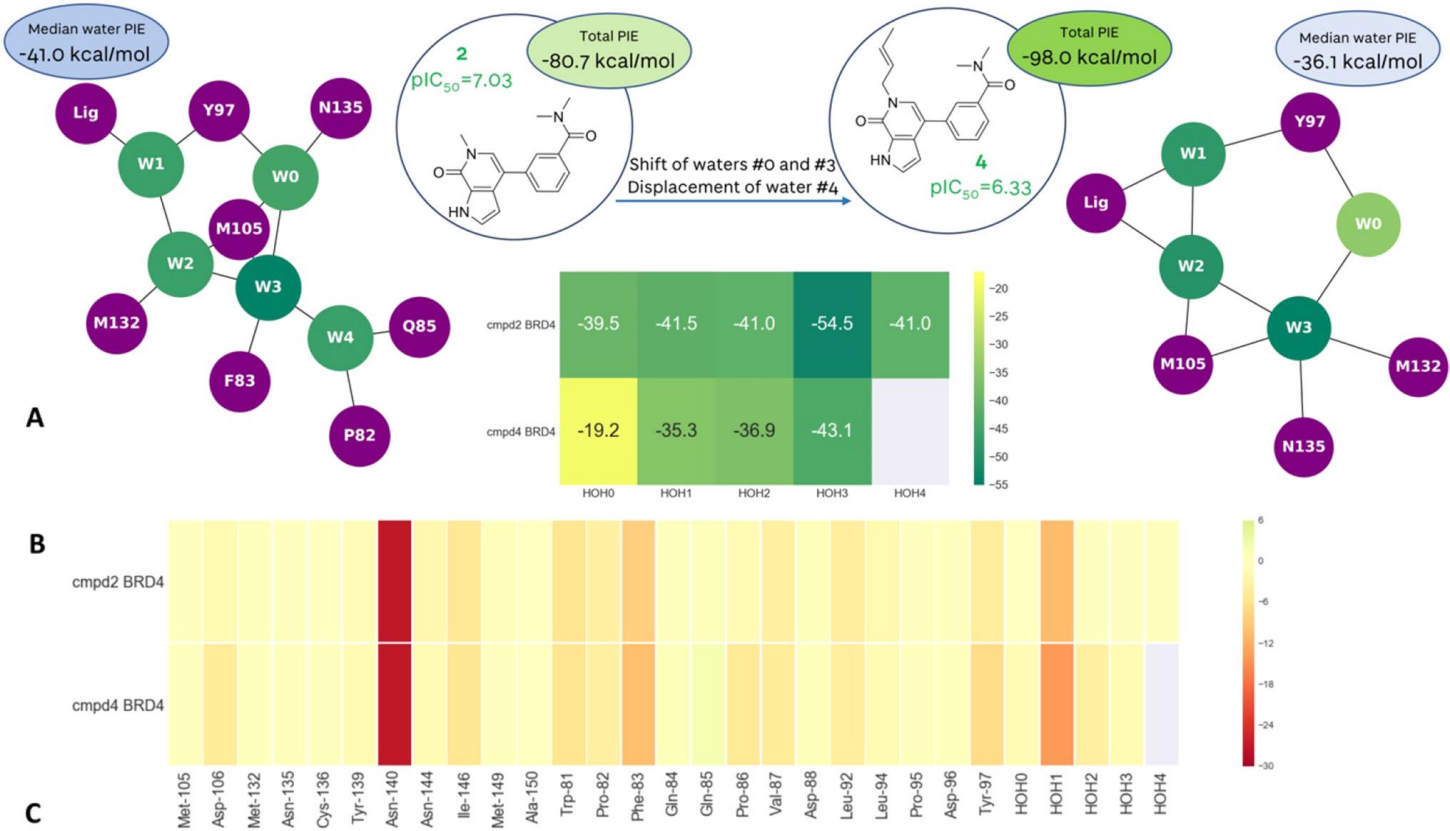
FMO results for the BRD4 in complex with **2** and **4**. **A** Extension further into the water network displaces water #4 and causes the waters #0 and #3 to rearrange. **B** Energetic contributions of individual water molecules to the stability of the overall water network. **C** FMO-PIE generated contribution to ligand binding energy in kcal/mol per fragment residues. Red indicates favourable interaction energies, whereas green indicates unfavourable interaction energies. The sum of these interactions is shown in Table 1 as *Ligand PIE*

The same compounds bind in both BRD4 and TAF1, where they adopt a binding mode in which the different substitutions on the scaffold cause the disruption of the water network (Fig. 1B and C). Extending the ligand (**2** to **4**) leads to a drop of affinity for BRD4. This is not reproduced by protein–ligand calculated PIE: looking at the binding mode (Fig. 1B) and the heatmap in Fig. 3, it can be observed that the tail extending from the pyridone portion of the molecule **4** makes additional interactions with Phe83 and Asp106. However, the decrease in potency can be explained by taking into account the Water-Network PIE. The further extension of this tail into the water network displaces water #4 and causes the waters #0 and #3 to rearrange (water numbering consistent with Nittinger et al. [3] and Crawford et al. [13]). Water #3 recovers some of the lost energy through a newly established hydrogen bond with Asn135, whereas water #0 has lost the favourable interactions with Met105 backbone and Asn135 sidechain. Each of the four water molecules is less stable in this new arrangement, significantly affecting the overall stability of the water network, which in turn has a detrimental effect on the overall affinity (Fig. 3). The fact that the SAR cannot be explained just by visually analysing the protein–ligand interactions suggests that changes in the measured affinity are caused by differences in the energetics of the water network. The displacement of a water molecule from an active site is only favourable if the newly formed protein–ligand-water interactions outweigh the loss of energy caused by disrupting the water network.

**Fig. 4:**
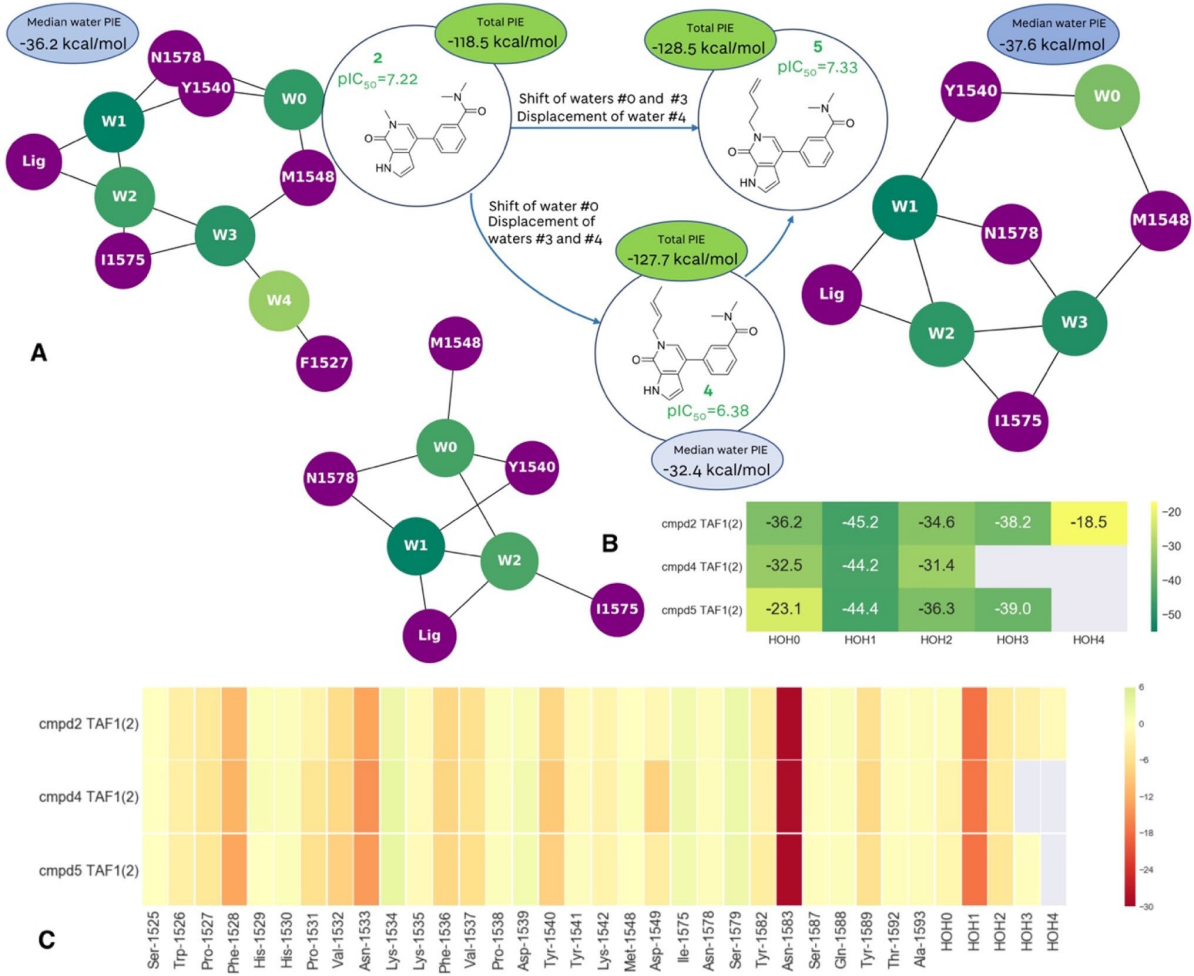
FMO results for the TAF1 in complex with **2**, **4** and **5**. **A** Extending the ligand leads to perturbance of water network and drop of affinity in **4**. In **5**, the water network is recovered, and affinity maintained. **B** Energetic contributions of individual water molecules to the stability of the overall water network. Change in the median FMO calculated ‘happiness’ of the water network follows the expected trend. **C** FMO-PIE generated contribution to ligand binding energy in kcal/mol per fragment residues. Red indicates favourable interaction energies, whereas green indicates unfavourable interaction energies. The sum of these interactions is shown in Table 1 as *Ligand PIE*

Addition of the crotyl substituent also led to the drop of affinity for TAF1 (Fig. 4). Here both waters #3 and #4 are displaced upon binding of **4**, and water #0 is shifted (Fig. 1C). The shift of the double bond to the terminal position in 1-butenyl substituent in **5** causes a slight conformational change of the alkyl chain so the water network is re-established: waters #3 and #0 are shifted, and water #4, the least stable water molecule in the complex, is displaced. Calculated PIE between the protein and the ligand does not fully explain the observed SAR as compounds **4** and **5** were calculated to be energetically equivalent, while **2** had the lowest complexation energy of the three.

Water #0 is the most stable in the complex of TAF1 with **2**, with the PIE value similar to the other two bromodomain structures (BRD4 and BRD9) in complex with the same ligand. It is tetracoordinated to Asn1578, Tyr1540, and the Met1548 backbone (engaging both NH and carbonyl oxygen). With compound **4** in TAF1, displacement of water molecules #3 and #4 causes the shift of water molecule #0, and subsequently, loss of a H-bond with the Met1548 backbone carbonyl. Some of this energy is recovered through a H-bond with water #2, whereas in **5** this does not seem to be possible, as water #3 is shifted due to the presence of the ligand substituent in such a way that it compensates loss of an H-bond with water #4 by forming an energetically comparable H-bond with the Met1548 backbone carbonyl. Although shifted, water energetics remain the same. The experimental results suggest that the average score of the water network should be favourable for **2**, decrease for **4**, and increase again for **5**. This behaviour was successfully identified by FMO: the water network is favourable for **2**, there is a decrease in the energy contribution for **4**, and again gain in energy upon re-establishing the hydrogen bond network in **5**. Similar to TAF1, gains and losses in affinity cannot be explained just by protein–ligand interactions. It is the combination of both water network stability and protein–ligand binding that helps interpret the observed SAR. As shown in the examples here, even if new protein–ligand contacts are formed, if the stability of the water network is compromised, this could be detrimental to the potency. Similarly, potency gains can be achieved by stabilising the water network, even if direct contacts between ligand and protein are weaker.

**Fig. 5:**
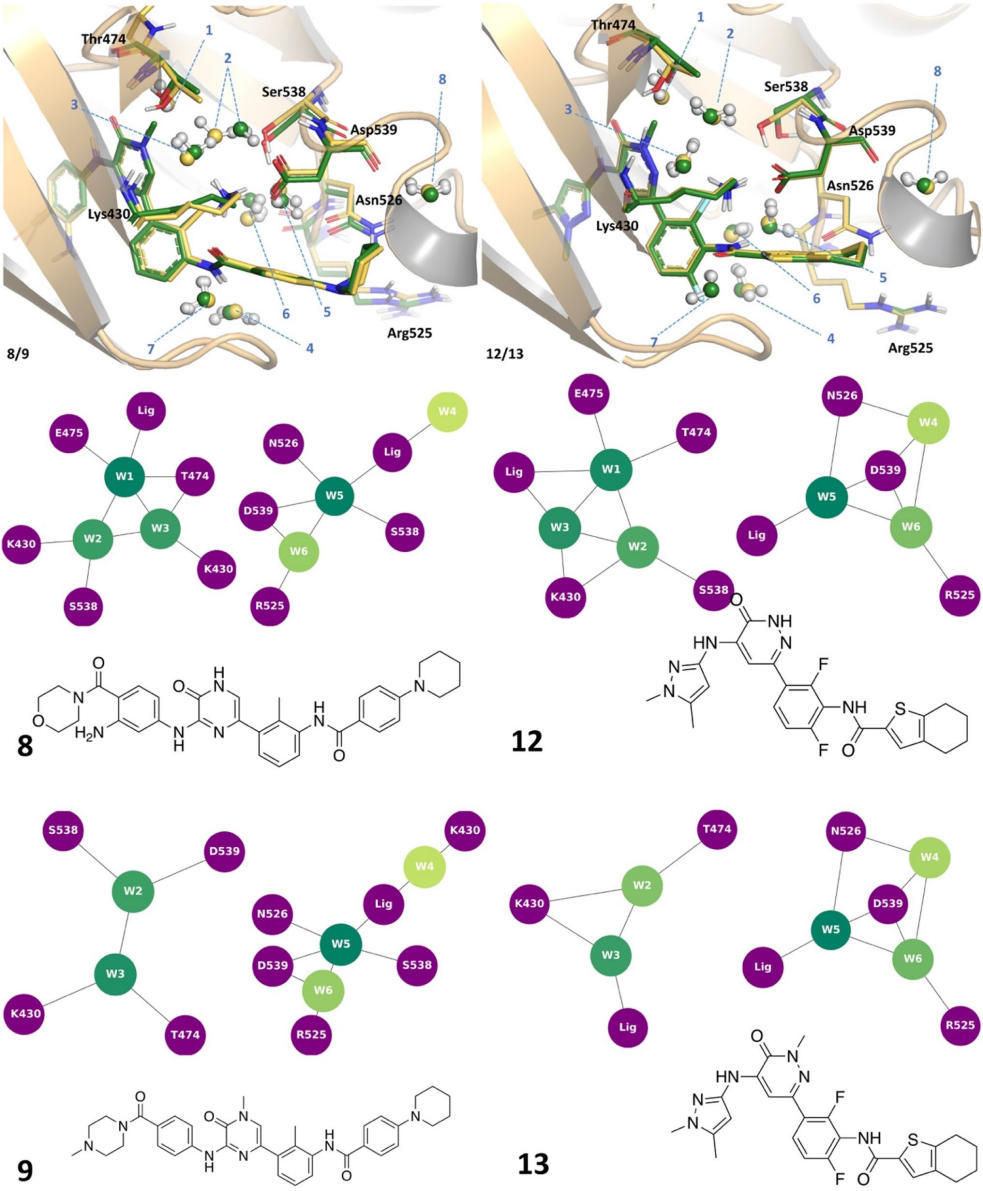
Structures of BTK pairs **8** (yellow)/**9** (green) and **12** (yellow)/**13** (green) and water network graphs

A second example was investigated, that of Bruton’s Tyrosine Kinase (Table 2). In particular, eight different structures from the protein databank were analysed. Energetics of eight structural waters were analysed as a part of the conserved network in BTK. The waters are numbered according to Nittinger et al. [3] Water #1 was displaced in two x-ray structure pairs: **8**/**9** and **12**/**13** (Fig. 5).

**Table 2:** Bruton’s Tyrosine Kinase (BTK) structures [29–32]

Compound	PDB code [3]	pIC_50_ [13]	Ligand PIE (kcal/mol)	Water network PIE (kcal/mol)	Water PIE median
**6**	6AUB	7.92	− 149.0	− 263.3	− 35.9
**7**	6BIK	8.19	− 184.5	− 174.0	− 30.1
**7_A**	6BIK	8.19	− 189.2	− 211.6	− 28.6
**7_B**	6BIK	8.19	− 186.5	− 238.0	− 33.8
**8_A**	6AUA	8.88	− 169.9	− 277.8	− 36.2
**8_B**	6AUA	8.88	− 169.0	− 278.8	− 37.2
**9**	6EP9	6.83	− 150.3	− 214.6	− 37.2
**10_A**	6BKH	8.88	− 200.1	− 271.2	− 33.9
**10_B**	6BKH	8.88	− 204.0	− 285.0	− 34.0
**11**	6BKE	8.34	− 206.3	− 226.3	− 33.5
**12**	6BLN	8.79	− 164.4	− 305.0	− 40.7
**13**	6BKW	8.30	− 159.0	− 235.2	− 32.3
**14**	5VFI	9.04	− 189.9	− 215.5	− 34.7

“Water network PIE” is the total of all the water energies, whereas “Water PIE median” is the median value over the individual water molecules. Median was used as it gives a more appropriate idea of the data distribution. The water molecule #6 in the 6BIK complex has been either removed (compound **7**) or represented in two different positions (compounds **7A** and **7B**). In complex 6AUA, compound **8** is reported in two possible conformations (compounds **8A** and **8B**). In complex 6BKH, Ser538 can adopt two possible side chain conformations (compounds **10A** and **10B**, Fig. SI3)

The crystal structure of BTK in complex with **8** was deposited in the PDB with the aniline in two different positions. FMO calculations were run on both ligand geometries (**8A** and **8B** in Table 2), and the results were equivalent (both in terms of protein ligand PIE and water network energetics), suggesting that both orientations are present in the conformational ensemble. Methylated pyridazinone in **9** and **13** displaces the water #1, leading to a decrease in affinity. This is correctly reproduced in protein–ligand PIE for the pair **8**/**9** (PIE − 169 kcal/mol for **8** and − 150.3 kcal/mol for **9**), and the calculated protein–ligand complexation energy for **12** is slightly higher than **13** (− 164.4 kcal/mol for **12**, − 159.0 kcal/mol for **13**). Loss of stable tetracoordinated water #1 (Ligand, #2, Glu475 and Thr474) causes the reorientation of water #2 in the complex with **9** in such a way that it compensates for the interactions lost by now engaging sidechain of Asp539, rather than Lys430 and water #1. This movement is slightly more favourable for water #3 as well. In complex with compound **13**, there is an additional, highly stable water molecule H-bonded to Lys430, Asp539, and waters #2 and #3 (PIE − 45.1 kcal/mol) (Fig. SI1).

Since this additional water molecule was observed only in this structure, both cases were evaluated in FMO. In the presence of the additional water molecule, the bond to water #2 is broken in favour of stabilising this additional water molecule. When this water is not present, water #3 is stabilised by Lys430, water #2 and the nitrogen of pyridazine. In any case, the overall water network is significantly less stable in **13** (median value − 32.3 kcal/mol) compared to **12** (− 40.7 kcal/mol), which contributes to the observed difference in potency. Experimental and computational results suggest water #1 is relatively stable and difficult to displace; it is the combination of both water network stability and protein–ligand binding that helps interpret the observed SAR.

**Fig. 6:**
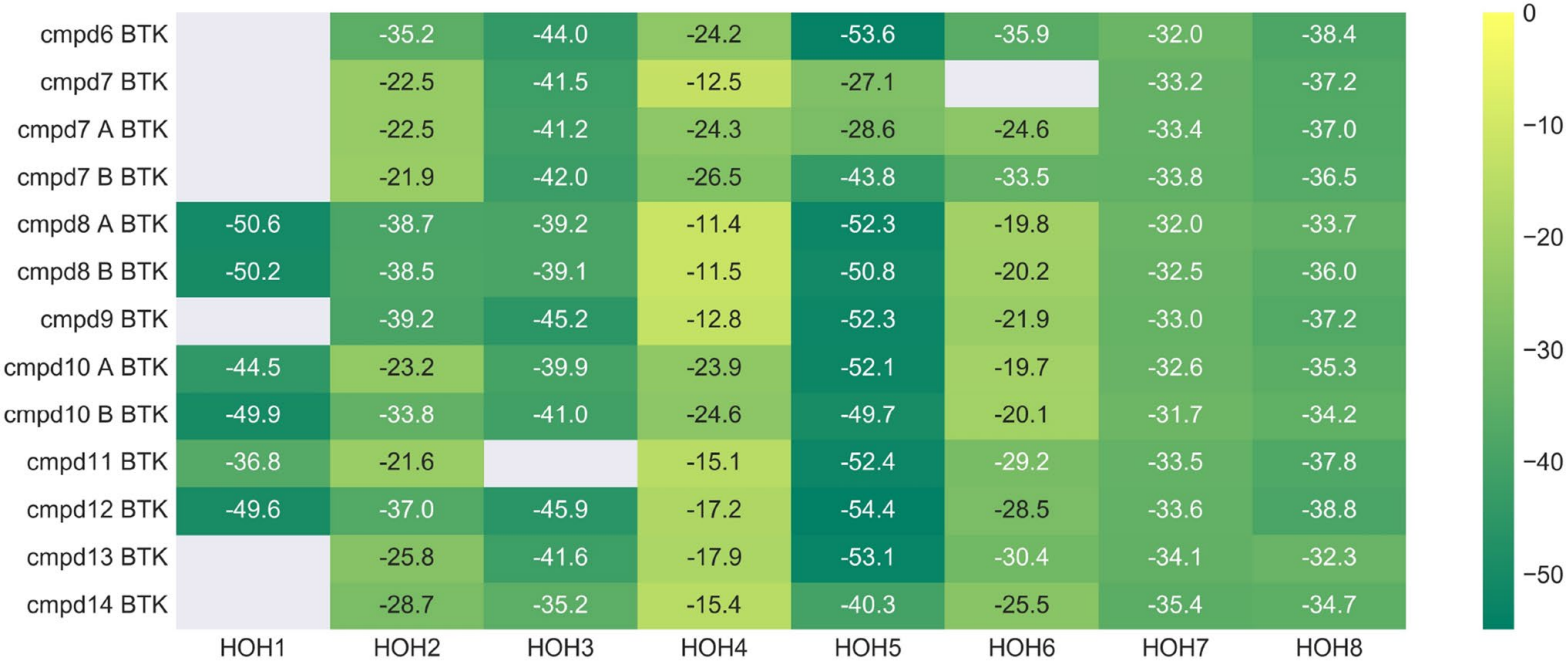
Energetic contributions of individual water molecules to the stability of the overall BTK water network

**Fig. 7:**
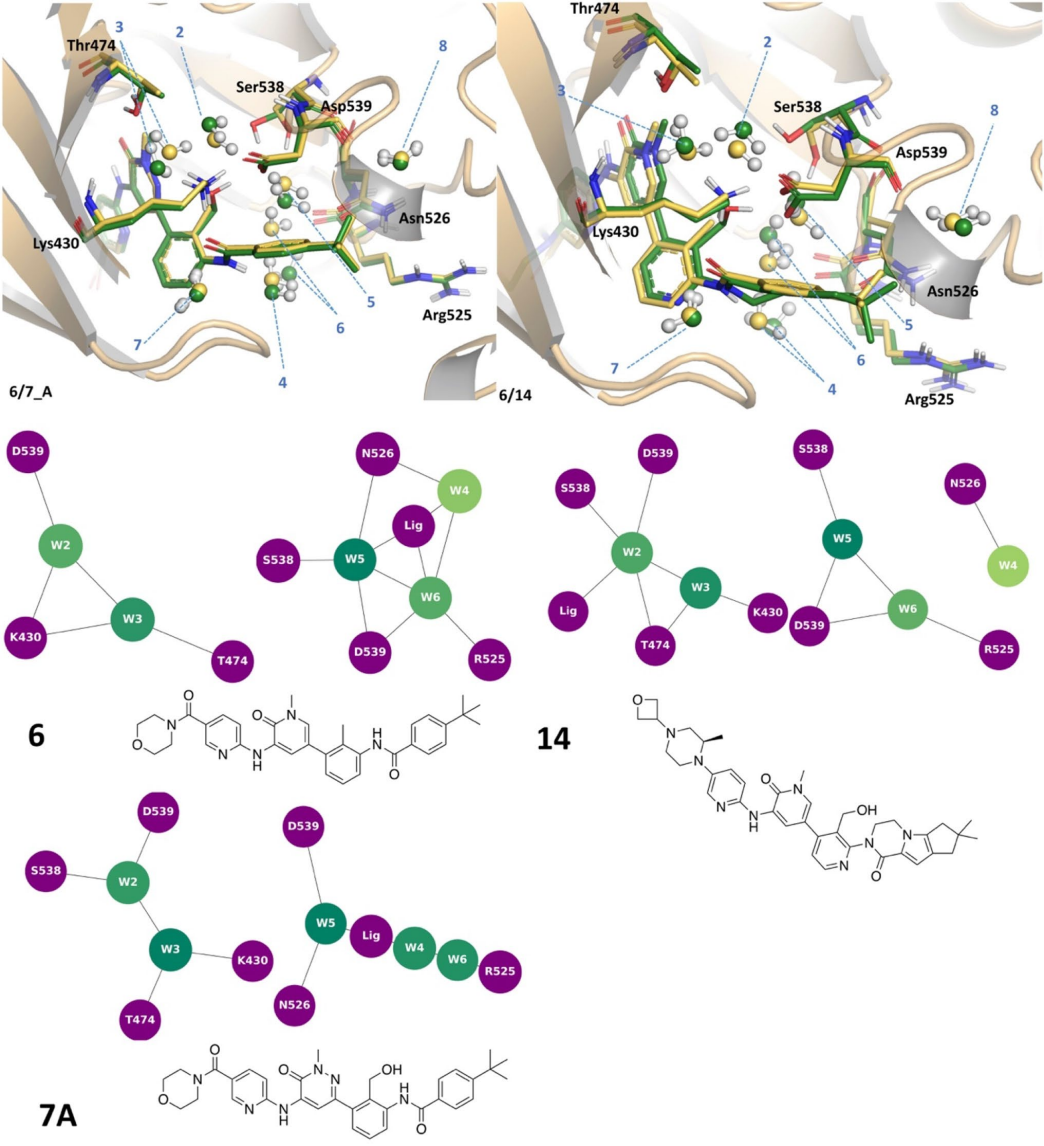
Structures of BTK pairs **6** (yellow)/**7** (green) and **6** (yellow)/**14** (green) and water network graphs

In the pair **6**/**7**, the methyl on the central phenyl ring in compound **6** is replaced by a hydroxymethyl moiety in compound **7**. In the complex with structure **7** the water molecule #6 seems to be present in two different positions with 0.45 and 0.55 occupancy. Therefore, three structures of **7** were evaluated in FMO: one with the water #6 missing (**7**), one with water #6 in the same position as it is in the complex with **6** (**7B**, occupancy 0.45 in the original PDB structure, 6BIK), and one where this water is shifted 3.4 Å (**7A**, occupancy 0.55 in the original PDB structure). The protein–ligand PIE is higher for **7** than **6 (**− 184.5 (**7**)/− 189.2 (**7A**) kcal/mol, compared to − 149.0 kcal/mol), which is in line with the observed SAR. All three representations of complex **7** structures have comparable protein–ligand PIEs, and detailed inspection of the associated FMO-PIE results reveals no significant direct interactions between compound **7** and water #6 (Fig. SI2). The water network appears to be more stable in **6**. Water #6 in the complex with **6** is stabilised by waters #4 and #5, the backbone of Arg525, as well as a weaker long-range electrostatic interaction with Asp539. When this water molecule is displaced, waters #4 and #5 become a lot less stable (from − 24.2 to − 12.5 kcal/mol (#4), − 53.6 to − 27.1 kcal/mol (#5)—Fig. 6). When water #6 is shifted, water #4 reorients in such a way that it establishes a H-bond with water #6. The presence of a longer hydroxymethyl substituent in **7** causes a sidechain flip of Ser538 (Fig. 7). The sidechain of this residue forms a H-bond with water #5 in **6**, and this movement is reflected in a slight loss of stability of water #5 in **7B**. The presence of the pyridazine (instead of pyridine in **6**), as well as the hydroxymethyl also causes a small shift in position of water #3, which has a destabilising effect on water #2. In the case of this pair (**6** and **7**), the newly formed protein–ligand interactions do not result in a significant gain of potency as the disruption of the water network partially compensates for the gain in the ligand protein interaction energy (Table 2).

A similar example is the pair **6**/**14** (Fig. 7). The protein–ligand PIE (− 189.9 kcal/mol) is higher for **14**, which is the most potent compound in the series, than for **6** (− 149.0 kcal/mol). In compound **14** the amide has been cyclised, losing the NH donor that is interacting favourably with water #4. This water molecule was identified as the least stable in all of the structures (Fig. 6). All the water molecules (apart from #7 and #8) are less stable in **14**, compared to **6**. This is not very surprising given that **14** is the largest ligand, and as such is more likely to disrupt the canonical water network. Waters #2 and #3 reorient due to the rotation of Ser538 and the presence of the hydroxymethyl substituent (Fig. 7). Water #3 seems to be particularly destabilised in this new arrangement, as the lone pairs on the oxygen are now pointing towards the negatively charged Asp539, the residue making the strongest interaction with the ligand.

It is also instructive to look at the pair **7**/**14**, where there is the same hydroxymethyl substituent and Ser538 rotation. In **7**, the presence of the additional acceptor nitrogen in the pyridazine causes water molecules #2 and #3 to orient in a way to maximise the electrostatic complementarity with both protein and ligand. Due to the size of the ligand **14**, and consequently the subtle shifts in the position of the water molecules (#5 that affects the position of Ser538 sidechain), re-orientation of water molecules #2 and #3 is more difficult in the complex with **14**. However, in case of the **6**/**14** pair, the new interactions the ligand makes with the protein in **14** more than compensates for a marginal destabilisation of the water network (water median value of − 35.9 (**6**)/− 34.7 (**14**) kcal/mol).

**Fig. 8:**
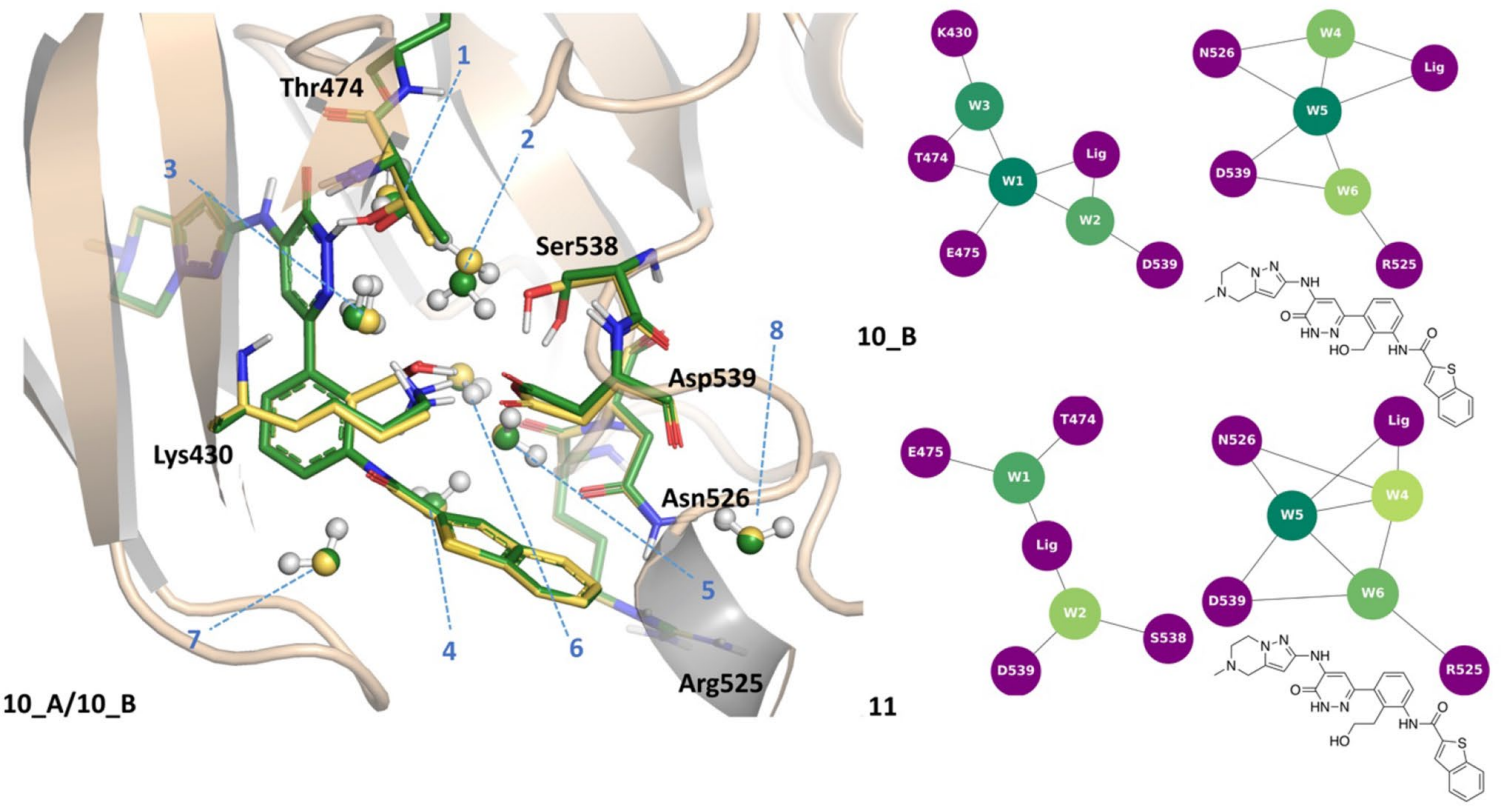
Structures of BTK pairs **10** (yellow)/**11** (green) and water network graphs

Compound **11** contains a hydroxyethyl group (equivalent to the hydroxymethyl in **10**) that causes the displacement of water #3 and, consequently, the shift of waters #4 and #6 (Fig. 8). The protein–ligand PIE is marginally higher for **11** (PIE − 206.3 kcal/mol) than for **10** (PIE − 200.1(A)/− 204.0 (B) kcal/mol), and the contribution of the water molecules network is comparable for the two structures. The complex with compound **10** has Ser538 modelled in two possible sidechain conformations, so that both scenarios A and B were evaluated. There is little difference in PIE between protein and ligand, but there are some subtle differences in water network orientation (waters #1 and #2 orient differently depending on whether or not Ser538 sidechain is available for H-bonding, **10 B**), as well as overall network stability, which is higher for **10 B** (Fig. 8). When comparing **10** and **11**, the water network is slightly more favourable for the more potent compound (**10**). In the presence of longer substituent in **11**, water #1 orients in such a way that it no longer can establish optimal interaction with water #2. Water #4 is shifted and is now stabilising water #6, rather than interacting with the ligand.

## Conclusions

In their comprehensive evaluation of water prediction programmes, Nittinger et al. [3] highlighted a critical limitation in the current approaches: each of the evaluated tools was capable of accurately predicting the location of most crystallographic water molecules, but predicting the effects of water displacement or water shift on ligand potency was inconsistent and considered to be of poor use for practical compound design.

One of the challenges in the description of the water molecules is related to the fact that these abundant molecules participate in hydrogen bonding both as a donor and acceptor. Water molecules at the protein–ligand interfaces form on average 3 hydrogen bonds [33]. A change in the orientation of a single water molecule in the binding site can affect the whole water network and not all interactions are created equal: depending on their context, non-covalent interactions will contribute differently to the overall binding. Judging the relative strength of these interactions by visual inspection, or even force-field based methods is difficult and highly inaccurate. Therefore, having tools capable of differentiating between strong and weak contacts would benefit both SAR rationalisation and prospective compound design.

The linear scaling QM FMO method provides the means by which water interaction energies can be assessed accurately and sufficiently fast. FMO has successfully been used to rationalise protein ligand binding and SAR, and is an invaluable tool that can provide insight into the chemical nature of noncovalent interactions [7, 10, 34]. In this work, it has been shown how the same approach can be used to assess stability (’happiness’) of individual water molecules and water networks.

Since the QM calculations presented here are single point energy calculations performed in vacuo, entropic and solvation effects are neglected. It is assumed that some of these cancel out when working with the congeneric chemical series, and some of the work published by others show good correlation of QM energies with the binding affinity [6–9, 34] and some of the published work tends to include additional parameters to account for these effects [8, 9, 11]. The changes in the SAR observed for the cases described in the manuscript are a combination of changes in the protein–ligand interactions, but also changes in the stability of the water network. At this point no attempt to quantitatively assess the degree of change was made, only a qualitative assessment if the change had a positive, detrimental or negligible effect. However, the approach we developed was capable of correlating changes in the water network to the measured SAR. Assessing whether water displacement had positive, detrimental or no effect on the overall compound potency is a challenging task. Water displacement is inseparably linked to the quality of the ligand designed to disturb the water network, making it difficult to objectively interpret the results. The calculations performed here show that although focusing on the’happiness’ of the individual water molecules can be instructive (e.g. the displacement of a happy water in BRD4 or BTK led to loss of potency, displacement of a ‘least happy’ water molecule in TAF1(2) did not), it is more instructive to look at the overall stability of the water network. The displacement of a water molecule from an active site is only favourable if the newly formed protein–ligand interactions outweigh the loss of energy caused by disrupting the water network. Given that the available programs for assessing crystallographic waters are successful at predicting the location of the water molecules, pairing them with an accurate assessment of water energetics such as the QM based approach described here, offers a new invaluable approach for prospective compound design.

## Supplementary Information

Below is the link to the electronic supplementary material.

Supplementary file1 (DOCX 987 kb)

## Data Availability

The datasets generated during and/or analysed during the current study are available from the corresponding author on reasonable request.
