# Supplementary material for: Ligand binding: evaluating the contribution of the water molecules network using the Fragment Molecular Orbital method
Source: J Comput Aided Mol Des. 2021 Aug 30;35(10):1025–36. doi: 10.1007/s10822-021-00416-3 (PMC8523014; doi:10.1007/s10822-021-00416-3)
Supplement: Supplementary file 1 — Supplementary file1 (DOCX 987 kb) [file 10822_2021_416_MOESM1_ESM.docx]

Ligand binding: Evaluating the contribution of the water molecules network using the Fragment Molecular Orbital method

Iva Lukac, Paul G. Wyatt, Ian H. Gilbert, and Fabio Zuccotto

Supplementary Information

Figure SI1 page 2

Figure SI2 page 3

Figure SI3 page 4

Sets of protein residues in PDB format used for FMO calculation:

BRD4 Protein page 5

BRD9 Protein page 12

BTK Protein page 19

TAF Protein page 27


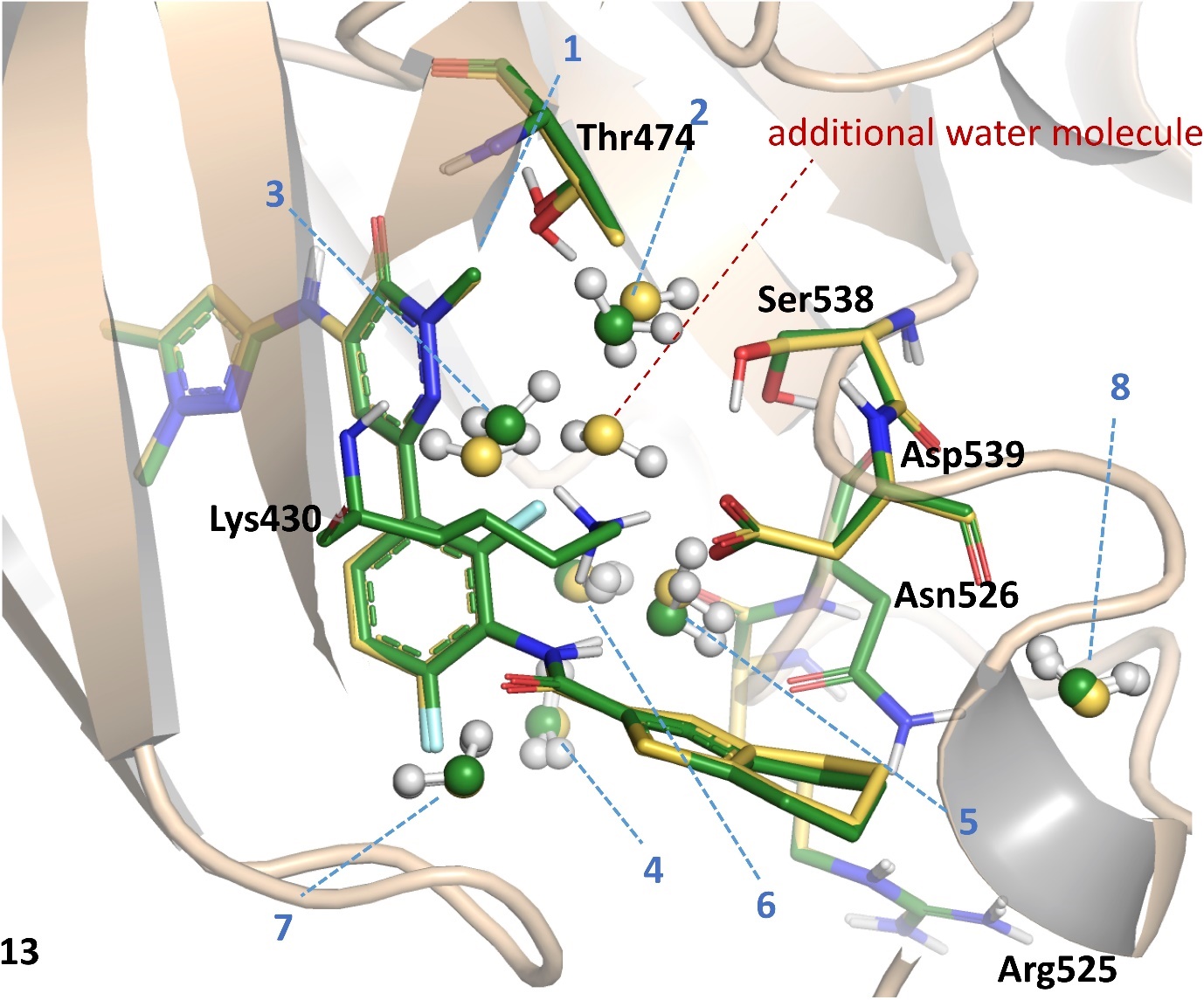


**Figure SI1**. Compound **13** with an additional highly stable water molecule H-bonded to Lys430, Asp539, and #2 and #3 (PIE -45.1 kcal/mol).


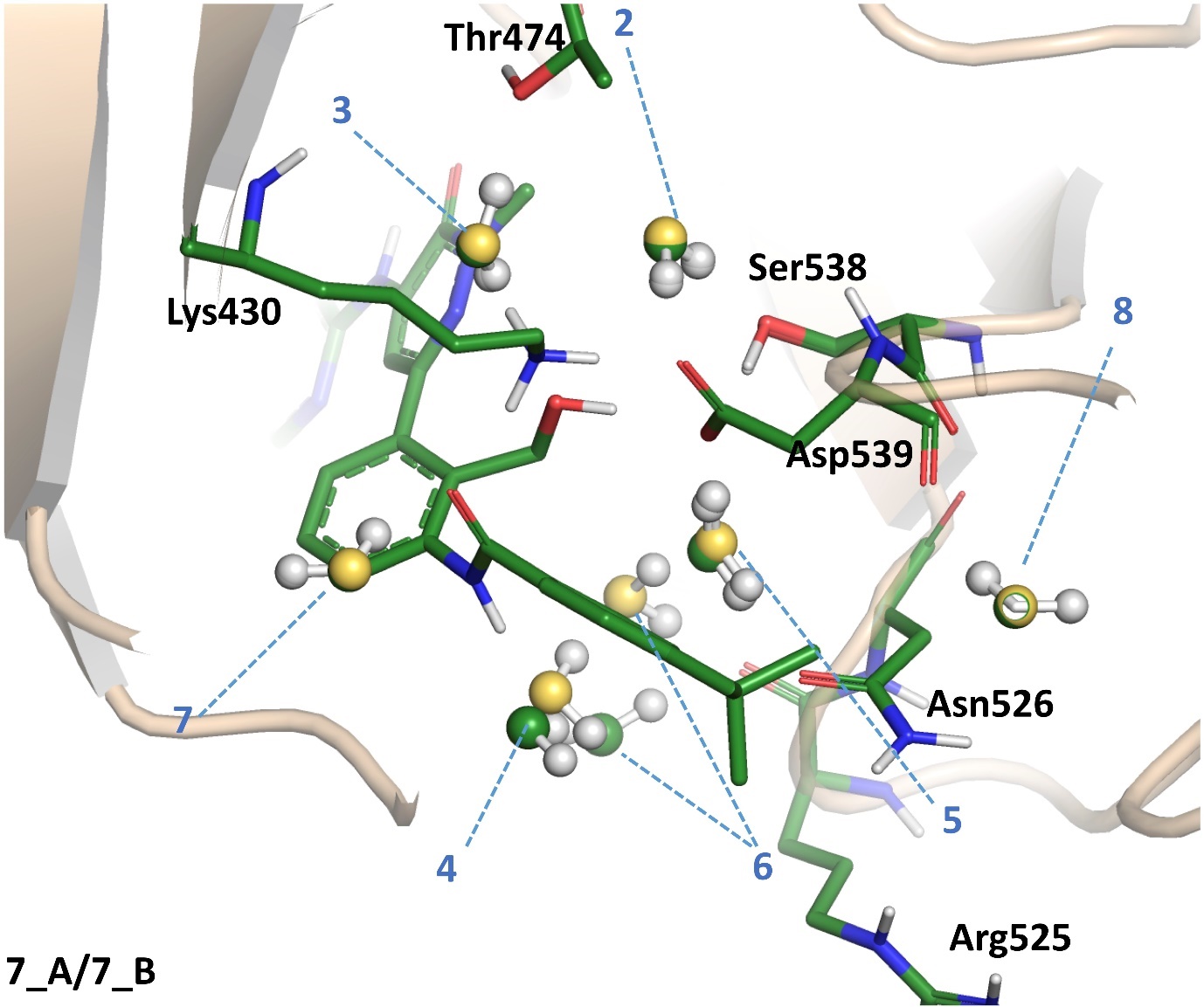


**Figure SI2.** Compound **7** complexed in BTK with water #6 in two different positions based on the PDB structure 6BIK


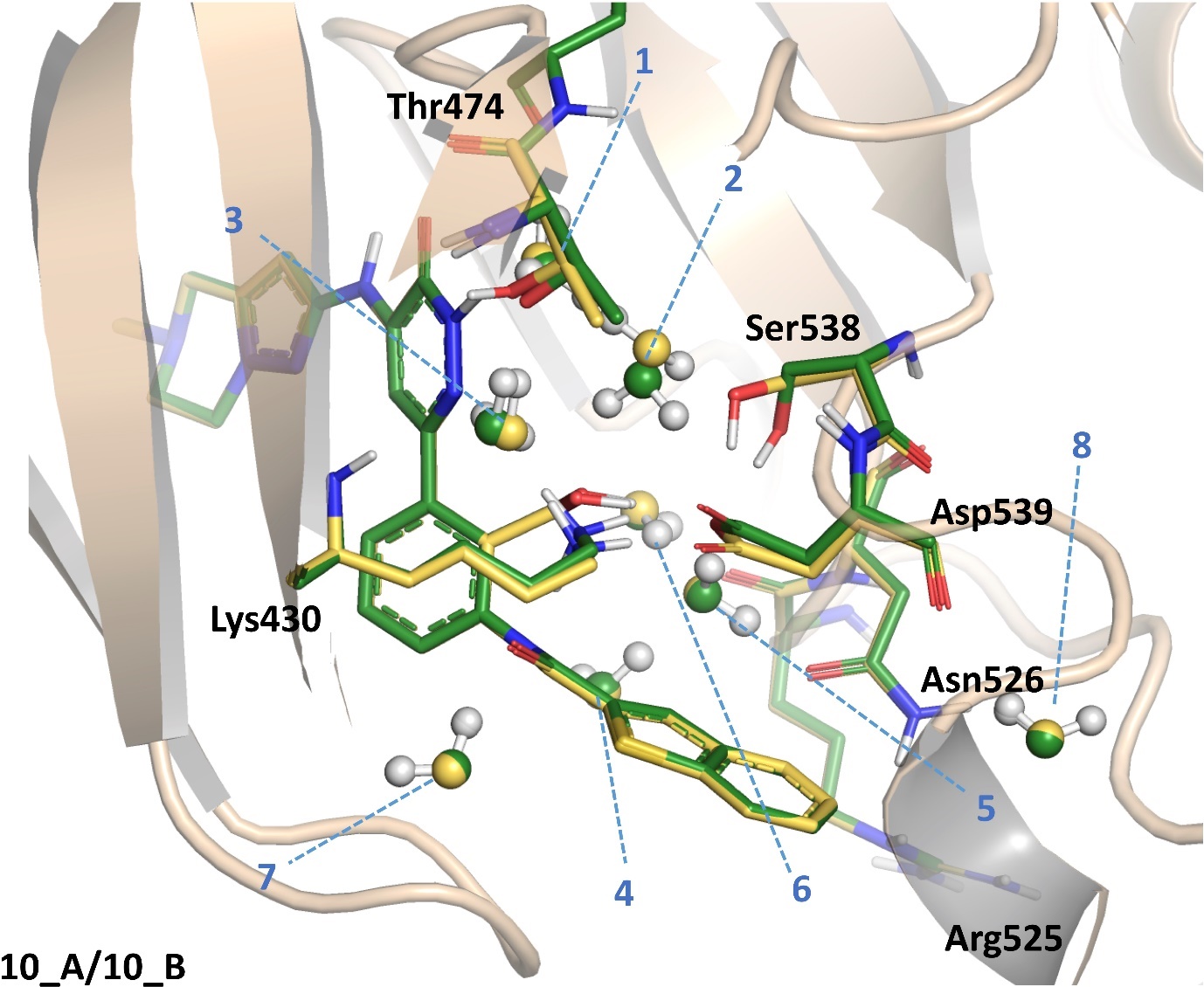


**Figure SI3.** Compound **10** in complex with BTK (PDB 6BKH) where Ser538 side chain can adopt two different conformations

**BRD4 Protein**

ATOM 1 CA ALA A 80 -5.391 1.670 11.602 1.00 23.95 C

ATOM 2 C ALA A 80 -6.845 1.584 12.082 1.00 21.21 C

ATOM 3 O ALA A 80 -7.158 0.736 12.916 1.00 20.35 O

ATOM 4 H01 ALA A 80 -5.100 2.716 11.508 1.00 23.95 H

ATOM 5 H02 ALA A 80 -5.298 1.178 10.634 1.00 23.95 H

ATOM 6 H03 ALA A 80 -4.741 1.176 12.324 1.00 23.95 H

ATOM 7 N TRP A 81 -7.691 2.504 11.601 1.00 21.06 N

ATOM 8 CA TRP A 81 -8.995 2.805 12.198 1.00 21.61 C

ATOM 9 C TRP A 81 -10.039 1.667 12.340 1.00 20.16 C

ATOM 10 O TRP A 81 -10.785 1.718 13.320 1.00 19.73 O

ATOM 11 CB TRP A 81 -9.585 4.106 11.620 1.00 21.16 C

ATOM 12 CG TRP A 81 -10.073 4.041 10.206 1.00 22.07 C

ATOM 13 CD1 TRP A 81 -9.324 4.248 9.099 1.00 22.86 C

ATOM 14 CD2 TRP A 81 -11.396 3.653 9.729 1.00 22.94 C

ATOM 15 CE2 TRP A 81 -11.377 3.640 8.301 1.00 23.46 C

ATOM 16 CE3 TRP A 81 -12.612 3.304 10.362 1.00 24.35 C

ATOM 17 NE1 TRP A 81 -10.091 4.014 7.975 1.00 22.99 N

ATOM 18 CZ2 TRP A 81 -12.508 3.296 7.541 1.00 24.88 C

ATOM 19 CZ3 TRP A 81 -13.757 2.977 9.610 1.00 26.07 C

ATOM 20 CH2 TRP A 81 -13.705 2.967 8.203 1.00 25.77 C

ATOM 21 H TRP A 81 -7.353 3.142 10.896 1.00 0.00 H

ATOM 22 HA TRP A 81 -8.754 3.052 13.229 1.00 0.00 H

ATOM 23 HB2 TRP A 81 -10.439 4.401 12.232 1.00 0.00 H

ATOM 24 HB3 TRP A 81 -8.868 4.921 11.717 1.00 0.00 H

ATOM 25 HD1 TRP A 81 -8.281 4.536 9.109 1.00 0.00 H

ATOM 26 HE1 TRP A 81 -9.722 4.113 7.032 1.00 0.00 H

ATOM 27 HE3 TRP A 81 -12.667 3.301 11.442 1.00 0.00 H

ATOM 28 HZ2 TRP A 81 -12.465 3.290 6.461 1.00 0.00 H

ATOM 29 HZ3 TRP A 81 -14.681 2.740 10.116 1.00 0.00 H

ATOM 30 HH2 TRP A 81 -14.586 2.712 7.631 1.00 0.00 H

ATOM 31 N PRO A 82 -10.048 0.615 11.485 1.00 19.80 N

ATOM 32 CA PRO A 82 -10.867 -0.584 11.750 1.00 18.89 C

ATOM 33 C PRO A 82 -10.428 -1.413 12.973 1.00 19.02 C

ATOM 34 O PRO A 82 -11.245 -2.155 13.517 1.00 20.10 O

ATOM 35 CB PRO A 82 -10.754 -1.401 10.454 1.00 19.40 C

ATOM 36 CG PRO A 82 -10.357 -0.400 9.389 1.00 20.15 C

ATOM 37 CD PRO A 82 -9.437 0.528 10.160 1.00 20.91 C

ATOM 38 HA PRO A 82 -11.901 -0.261 11.884 1.00 0.00 H

ATOM 39 HB2 PRO A 82 -9.967 -2.153 10.547 1.00 0.00 H

ATOM 40 HB3 PRO A 82 -11.672 -1.926 10.202 1.00 0.00 H

ATOM 41 HG2 PRO A 82 -9.890 -0.870 8.525 1.00 0.00 H

ATOM 42 HG3 PRO A 82 -11.235 0.155 9.052 1.00 0.00 H

ATOM 43 HD2 PRO A 82 -8.457 0.068 10.263 1.00 0.00 H

ATOM 44 HD3 PRO A 82 -9.321 1.483 9.652 1.00 0.00 H

ATOM 45 N PHE A 83 -9.158 -1.283 13.375 1.00 18.37 N

ATOM 46 CA PHE A 83 -8.513 -2.084 14.412 1.00 18.24 C

ATOM 47 C PHE A 83 -8.369 -1.299 15.730 1.00 18.46 C

ATOM 48 O PHE A 83 -7.882 -1.864 16.703 1.00 20.65 O

ATOM 49 CB PHE A 83 -7.133 -2.555 13.898 1.00 19.61 C

ATOM 50 CG PHE A 83 -7.159 -3.359 12.608 1.00 20.12 C

ATOM 51 CD1 PHE A 83 -7.468 -4.735 12.630 1.00 19.92 C

ATOM 52 CD2 PHE A 83 -7.038 -2.705 11.363 1.00 21.13 C

ATOM 53 CE1 PHE A 83 -7.587 -5.439 11.438 1.00 21.35 C

ATOM 54 CE2 PHE A 83 -7.166 -3.424 10.183 1.00 21.64 C

ATOM 55 CZ PHE A 83 -7.438 -4.785 10.221 1.00 21.61 C

ATOM 56 H PHE A 83 -8.535 -0.638 12.899 1.00 0.00 H

ATOM 57 HA PHE A 83 -9.104 -2.974 14.626 1.00 0.00 H

ATOM 58 HB2 PHE A 83 -6.474 -1.699 13.754 1.00 0.00 H

ATOM 59 HB3 PHE A 83 -6.656 -3.168 14.663 1.00 0.00 H

ATOM 60 HD1 PHE A 83 -7.619 -5.248 13.571 1.00 0.00 H

ATOM 61 HD2 PHE A 83 -6.845 -1.642 11.318 1.00 0.00 H

ATOM 62 HE1 PHE A 83 -7.820 -6.494 11.461 1.00 0.00 H

ATOM 63 HE2 PHE A 83 -7.066 -2.919 9.233 1.00 0.00 H

ATOM 64 HZ PHE A 83 -7.553 -5.333 9.296 1.00 0.00 H

ATOM 65 N GLN A 84 -8.783 -0.023 15.763 1.00 17.78 N

ATOM 66 CA GLN A 84 -8.712 0.860 16.936 1.00 18.18 C

ATOM 67 C GLN A 84 -9.753 0.561 18.039 1.00 19.41 C

ATOM 68 O GLN A 84 -9.708 1.196 19.093 1.00 20.29 O

ATOM 69 CB GLN A 84 -8.808 2.332 16.476 1.00 18.83 C

ATOM 70 H GLN A 84 -9.192 0.367 14.926 1.00 0.00 H

ATOM 71 HA GLN A 84 -7.737 0.718 17.404 1.00 0.00 H

ATOM 72 HB2 GLN A 84 -9.653 2.444 15.795 1.00 0.00 H

ATOM 73 HB3 GLN A 84 -9.040 2.972 17.330 1.00 0.00 H

ATOM 74 H01 GLN A 84 -7.861 2.602 16.009 1.00 18.83 H

ATOM 75 N GLN A 85 -10.686 -0.361 17.788 1.00 18.98 N

ATOM 76 CA GLN A 85 -11.778 -0.744 18.681 1.00 17.30 C

ATOM 77 C GLN A 85 -12.252 -2.162 18.291 1.00 17.49 C

ATOM 78 O GLN A 85 -11.895 -2.610 17.195 1.00 18.09 O

ATOM 79 CB GLN A 85 -12.909 0.326 18.647 1.00 19.93 C

ATOM 80 CG GLN A 85 -13.694 0.471 17.320 1.00 21.78 C

ATOM 81 CD GLN A 85 -12.913 1.227 16.247 1.00 23.05 C

ATOM 82 NE2 GLN A 85 -12.609 0.583 15.129 1.00 24.07 N

ATOM 83 OE1 GLN A 85 -12.576 2.394 16.431 1.00 26.83 O

ATOM 84 H GLN A 85 -10.648 -0.891 16.927 1.00 0.00 H

ATOM 85 HA GLN A 85 -11.365 -0.788 19.689 1.00 0.00 H

ATOM 86 HB2 GLN A 85 -13.631 0.098 19.427 1.00 0.00 H

ATOM 87 HB3 GLN A 85 -12.504 1.295 18.940 1.00 0.00 H

ATOM 88 HG2 GLN A 85 -14.033 -0.497 16.948 1.00 0.00 H

ATOM 89 HG3 GLN A 85 -14.601 1.045 17.511 1.00 0.00 H

ATOM 90 HE21 GLN A 85 -12.869 -0.383 14.983 1.00 0.00 H

ATOM 91 HE22 GLN A 85 -12.053 1.053 14.414 1.00 0.00 H

ATOM 92 N PRO A 86 -13.066 -2.836 19.142 1.00 19.07 N

ATOM 93 CA PRO A 86 -13.666 -4.141 18.796 1.00 18.50 C

ATOM 94 C PRO A 86 -14.530 -4.104 17.523 1.00 18.17 C

ATOM 95 O PRO A 86 -15.216 -3.107 17.284 1.00 21.95 O

ATOM 96 CB PRO A 86 -14.519 -4.496 20.031 1.00 19.73 C

ATOM 97 CG PRO A 86 -13.895 -3.721 21.177 1.00 20.23 C

ATOM 98 CD PRO A 86 -13.433 -2.437 20.503 1.00 20.91 C

ATOM 99 HA PRO A 86 -12.848 -4.856 18.688 1.00 0.00 H

ATOM 100 HB2 PRO A 86 -15.552 -4.168 19.898 1.00 0.00 H

ATOM 101 HB3 PRO A 86 -14.543 -5.570 20.223 1.00 0.00 H

ATOM 102 HG2 PRO A 86 -14.575 -3.553 22.013 1.00 0.00 H

ATOM 103 HG3 PRO A 86 -13.030 -4.267 21.556 1.00 0.00 H

ATOM 104 HD2 PRO A 86 -14.262 -1.731 20.459 1.00 0.00 H

ATOM 105 HD3 PRO A 86 -12.614 -1.984 21.063 1.00 0.00 H

ATOM 106 N VAL A 87 -14.504 -5.197 16.750 1.00 18.37 N

ATOM 107 CA VAL A 87 -15.372 -5.417 15.592 1.00 18.75 C

ATOM 108 C VAL A 87 -16.851 -5.409 16.031 1.00 18.69 C

ATOM 109 O VAL A 87 -17.248 -6.231 16.861 1.00 19.33 O

ATOM 110 CB VAL A 87 -15.060 -6.776 14.898 1.00 19.13 C

ATOM 111 CG1 VAL A 87 -16.044 -7.171 13.776 1.00 20.80 C

ATOM 112 CG2 VAL A 87 -13.627 -6.785 14.340 1.00 20.52 C

ATOM 113 H VAL A 87 -13.912 -5.976 16.998 1.00 0.00 H

ATOM 114 HA VAL A 87 -15.197 -4.606 14.881 1.00 0.00 H

ATOM 115 HB VAL A 87 -15.116 -7.567 15.649 1.00 0.00 H

ATOM 116 HG11 VAL A 87 -15.743 -8.110 13.312 1.00 0.00 H

ATOM 117 HG12 VAL A 87 -17.063 -7.321 14.134 1.00 0.00 H

ATOM 118 HG13 VAL A 87 -16.063 -6.419 12.989 1.00 0.00 H

ATOM 119 HG21 VAL A 87 -13.416 -7.703 13.792 1.00 0.00 H

ATOM 120 HG22 VAL A 87 -13.466 -5.951 13.658 1.00 0.00 H

ATOM 121 HG23 VAL A 87 -12.883 -6.710 15.133 1.00 0.00 H

ATOM 122 N ASP A 88 -17.627 -4.465 15.494 1.00 19.76 N

ATOM 123 CA ASP A 88 -19.049 -4.346 15.788 1.00 21.15 C

ATOM 124 C ASP A 88 -19.799 -5.160 14.732 1.00 21.52 C

ATOM 125 O ASP A 88 -20.074 -4.660 13.642 1.00 22.48 O

ATOM 126 CB ASP A 88 -19.513 -2.880 15.839 1.00 22.71 C

ATOM 127 CG ASP A 88 -20.892 -2.743 16.473 1.00 24.54 C

ATOM 128 OD1 ASP A 88 -21.792 -3.567 16.208 1.00 22.57 O

ATOM 129 OD2 ASP A 88 -21.070 -1.763 17.220 1.00 27.19 O1-

ATOM 130 H ASP A 88 -17.254 -3.837 14.797 1.00 0.00 H

ATOM 131 HA ASP A 88 -19.260 -4.777 16.770 1.00 0.00 H

ATOM 132 HB2 ASP A 88 -18.803 -2.318 16.447 1.00 0.00 H

ATOM 133 HB3 ASP A 88 -19.523 -2.417 14.852 1.00 0.00 H

ATOM 134 N ALA A 89 -20.105 -6.411 15.075 1.00 22.48 N

ATOM 135 CA ALA A 89 -20.814 -7.348 14.217 1.00 23.21 C

ATOM 136 H ALA A 89 -19.954 -6.699 16.038 1.00 0.00 H

ATOM 137 HA ALA A 89 -20.278 -7.398 13.268 1.00 0.00 H

ATOM 138 H01 ALA A 89 -21.834 -7.002 14.051 1.00 23.21 H

ATOM 139 H02 ALA A 89 -20.860 -8.333 14.682 1.00 23.21 H

ATOM 140 CA LEU A 92 -20.968 -3.930 10.216 1.00 26.48 C

ATOM 141 C LEU A 92 -21.756 -5.013 9.456 1.00 28.38 C

ATOM 142 O LEU A 92 -21.430 -5.279 8.300 1.00 29.68 O

ATOM 143 CB LEU A 92 -19.545 -4.427 10.538 1.00 26.13 C

ATOM 144 CG LEU A 92 -18.608 -3.341 11.110 1.00 25.06 C

ATOM 145 CD1 LEU A 92 -17.349 -3.978 11.719 1.00 24.65 C

ATOM 146 CD2 LEU A 92 -18.260 -2.259 10.070 1.00 25.84 C

ATOM 147 HB2 LEU A 92 -19.619 -5.269 11.227 1.00 0.00 H

ATOM 148 HB3 LEU A 92 -19.084 -4.837 9.638 1.00 0.00 H

ATOM 149 HG LEU A 92 -19.122 -2.848 11.935 1.00 0.00 H

ATOM 150 HD11 LEU A 92 -16.902 -3.324 12.467 1.00 0.00 H

ATOM 151 HD12 LEU A 92 -17.582 -4.921 12.211 1.00 0.00 H

ATOM 152 HD13 LEU A 92 -16.596 -4.186 10.960 1.00 0.00 H

ATOM 153 HD21 LEU A 92 -17.185 -2.115 9.984 1.00 0.00 H

ATOM 154 HD22 LEU A 92 -18.626 -2.504 9.073 1.00 0.00 H

ATOM 155 HD23 LEU A 92 -18.690 -1.297 10.350 1.00 0.00 H

ATOM 156 H01 LEU A 92 -21.486 -3.694 11.146 1.00 26.48 H

ATOM 157 H02 LEU A 92 -20.898 -3.040 9.591 1.00 26.48 H

ATOM 158 N ASN A 93 -22.770 -5.611 10.102 1.00 30.16 N

ATOM 159 CA ASN A 93 -23.615 -6.701 9.598 1.00 33.15 C

ATOM 160 C ASN A 93 -22.766 -7.957 9.300 1.00 31.49 C

ATOM 161 O ASN A 93 -22.699 -8.421 8.161 1.00 32.21 O

ATOM 162 H ASN A 93 -22.956 -5.320 11.052 1.00 0.00 H

ATOM 163 H01 ASN A 93 -24.110 -6.377 8.682 1.00 33.15 H

ATOM 164 H02 ASN A 93 -24.355 -6.951 10.358 1.00 33.15 H

ATOM 165 N LEU A 94 -22.120 -8.468 10.353 1.00 29.43 N

ATOM 166 CA LEU A 94 -21.256 -9.650 10.327 1.00 28.59 C

ATOM 167 C LEU A 94 -21.775 -10.681 11.359 1.00 28.24 C

ATOM 168 O LEU A 94 -21.078 -10.946 12.341 1.00 26.37 O

ATOM 169 CB LEU A 94 -19.789 -9.231 10.627 1.00 27.62 C

ATOM 170 CG LEU A 94 -19.158 -8.153 9.719 1.00 28.40 C

ATOM 171 CD1 LEU A 94 -17.745 -7.791 10.225 1.00 27.41 C

ATOM 172 CD2 LEU A 94 -19.156 -8.531 8.224 1.00 29.24 C

ATOM 173 H LEU A 94 -22.275 -8.047 11.267 1.00 0.00 H

ATOM 174 HA LEU A 94 -21.278 -10.135 9.351 1.00 0.00 H

ATOM 175 HB2 LEU A 94 -19.722 -8.890 11.660 1.00 0.00 H

ATOM 176 HB3 LEU A 94 -19.163 -10.121 10.572 1.00 0.00 H

ATOM 177 HG LEU A 94 -19.763 -7.253 9.823 1.00 0.00 H

ATOM 178 HD11 LEU A 94 -17.587 -6.712 10.219 1.00 0.00 H

ATOM 179 HD12 LEU A 94 -17.571 -8.131 11.247 1.00 0.00 H

ATOM 180 HD13 LEU A 94 -16.959 -8.236 9.616 1.00 0.00 H

ATOM 181 HD21 LEU A 94 -18.187 -8.370 7.752 1.00 0.00 H

ATOM 182 HD22 LEU A 94 -19.419 -9.576 8.062 1.00 0.00 H

ATOM 183 HD23 LEU A 94 -19.879 -7.926 7.676 1.00 0.00 H

ATOM 184 N PRO A 95 -23.014 -11.207 11.196 1.00 30.06 N

ATOM 185 CA PRO A 95 -23.679 -12.001 12.254 1.00 31.07 C

ATOM 186 C PRO A 95 -22.980 -13.318 12.648 1.00 30.58 C

ATOM 187 O PRO A 95 -23.227 -13.823 13.743 1.00 32.43 O

ATOM 188 CB PRO A 95 -25.089 -12.252 11.690 1.00 32.76 C

ATOM 189 CG PRO A 95 -24.914 -12.194 10.180 1.00 32.67 C

ATOM 190 CD PRO A 95 -23.868 -11.099 10.010 1.00 31.91 C

ATOM 191 HA PRO A 95 -23.754 -11.395 13.159 1.00 0.00 H

ATOM 192 HB2 PRO A 95 -25.535 -13.192 12.019 1.00 0.00 H

ATOM 193 HB3 PRO A 95 -25.755 -11.450 12.013 1.00 0.00 H

ATOM 194 HG2 PRO A 95 -24.514 -13.143 9.819 1.00 0.00 H

ATOM 195 HG3 PRO A 95 -25.840 -11.993 9.640 1.00 0.00 H

ATOM 196 HD2 PRO A 95 -23.321 -11.210 9.073 1.00 0.00 H

ATOM 197 HD3 PRO A 95 -24.361 -10.128 10.013 1.00 0.00 H

ATOM 198 N ASP A 96 -22.131 -13.854 11.767 1.00 29.61 N

ATOM 199 CA ASP A 96 -21.356 -15.077 11.954 1.00 29.55 C

ATOM 200 C ASP A 96 -19.966 -14.801 12.557 1.00 27.00 C

ATOM 201 O ASP A 96 -19.273 -15.770 12.864 1.00 25.37 O

ATOM 202 CB ASP A 96 -21.202 -15.901 10.646 1.00 33.21 C

ATOM 203 CG ASP A 96 -20.660 -15.148 9.428 1.00 36.59 C

ATOM 204 OD1 ASP A 96 -20.626 -13.898 9.439 1.00 36.37 O

ATOM 205 OD2 ASP A 96 -20.333 -15.832 8.439 1.00 38.98 O1-

ATOM 206 H ASP A 96 -21.926 -13.397 10.880 1.00 0.00 H

ATOM 207 HA ASP A 96 -21.878 -15.712 12.669 1.00 0.00 H

ATOM 208 HB2 ASP A 96 -20.550 -16.755 10.837 1.00 0.00 H

ATOM 209 HB3 ASP A 96 -22.180 -16.303 10.382 1.00 0.00 H

ATOM 210 N TYR A 97 -19.565 -13.531 12.748 1.00 24.83 N

ATOM 211 CA TYR A 97 -18.225 -13.163 13.228 1.00 22.93 C

ATOM 212 C TYR A 97 -17.868 -13.843 14.558 1.00 23.70 C

ATOM 213 O TYR A 97 -16.877 -14.565 14.632 1.00 23.70 O

ATOM 214 CB TYR A 97 -18.053 -11.630 13.305 1.00 21.05 C

ATOM 215 CG TYR A 97 -16.645 -11.187 13.668 1.00 19.85 C

ATOM 216 CD1 TYR A 97 -15.684 -11.024 12.653 1.00 20.49 C

ATOM 217 CD2 TYR A 97 -16.269 -10.996 15.014 1.00 19.82 C

ATOM 218 CE1 TYR A 97 -14.354 -10.707 12.979 1.00 20.09 C

ATOM 219 CE2 TYR A 97 -14.937 -10.680 15.344 1.00 19.63 C

ATOM 220 CZ TYR A 97 -13.974 -10.544 14.327 1.00 19.36 C

ATOM 221 OH TYR A 97 -12.679 -10.264 14.647 1.00 20.16 O

ATOM 222 H TYR A 97 -20.183 -12.770 12.489 1.00 0.00 H

ATOM 223 HA TYR A 97 -17.512 -13.535 12.492 1.00 0.00 H

ATOM 224 HB2 TYR A 97 -18.298 -11.193 12.339 1.00 0.00 H

ATOM 225 HB3 TYR A 97 -18.758 -11.201 14.018 1.00 0.00 H

ATOM 226 HD1 TYR A 97 -15.962 -11.164 11.620 1.00 0.00 H

ATOM 227 HD2 TYR A 97 -16.992 -11.126 15.802 1.00 0.00 H

ATOM 228 HE1 TYR A 97 -13.630 -10.604 12.184 1.00 0.00 H

ATOM 229 HE2 TYR A 97 -14.656 -10.554 16.380 1.00 0.00 H

ATOM 230 HH TYR A 97 -12.137 -10.119 13.834 1.00 0.00 H

ATOM 231 N TYR A 98 -18.745 -13.685 15.555 1.00 23.87 N

ATOM 232 CA TYR A 98 -18.573 -14.254 16.891 1.00 23.18 C

ATOM 233 H TYR A 98 -19.559 -13.107 15.410 1.00 0.00 H

ATOM 234 HA TYR A 98 -17.506 -14.266 17.115 1.00 0.00 H

ATOM 235 H01 TYR A 98 -18.969 -15.269 16.916 1.00 23.18 H

ATOM 236 H02 TYR A 98 -19.109 -13.658 17.629 1.00 23.18 H

ATOM 237 CA PRO A 104 -11.566 -11.173 19.581 1.00 18.68 C

ATOM 238 C PRO A 104 -10.499 -10.291 20.251 1.00 17.94 C

ATOM 239 O PRO A 104 -10.307 -10.395 21.464 1.00 19.59 O

ATOM 240 HA PRO A 104 -11.340 -11.273 18.520 1.00 0.00 H

ATOM 241 H01 PRO A 104 -12.547 -10.714 19.703 1.00 18.68 H

ATOM 242 H02 PRO A 104 -11.567 -12.159 20.047 1.00 18.68 H

ATOM 243 N MET A 105 -9.821 -9.453 19.462 1.00 16.65 N

ATOM 244 CA MET A 105 -8.784 -8.549 19.954 1.00 16.44 C

ATOM 245 C MET A 105 -8.639 -7.347 19.009 1.00 16.58 C

ATOM 246 O MET A 105 -9.019 -7.441 17.843 1.00 18.38 O

ATOM 247 H MET A 105 -10.042 -9.372 18.469 1.00 0.00 H

ATOM 248 H01 MET A 105 -7.835 -9.083 20.007 1.00 16.44 H

ATOM 249 H02 MET A 105 -9.064 -8.191 20.945 1.00 16.44 H

ATOM 250 N ASP A 106 -8.113 -6.235 19.521 1.00 16.57 N

ATOM 251 CA ASP A 106 -8.070 -4.943 18.841 1.00 16.69 C

ATOM 252 C ASP A 106 -6.986 -4.083 19.516 1.00 16.87 C

ATOM 253 O ASP A 106 -6.598 -4.355 20.655 1.00 16.58 O

ATOM 254 CB ASP A 106 -9.432 -4.204 18.869 1.00 18.33 C

ATOM 255 CG ASP A 106 -9.909 -3.800 20.261 1.00 18.53 C

ATOM 256 OD1 ASP A 106 -10.450 -4.650 20.994 1.00 18.47 O

ATOM 257 OD2 ASP A 106 -9.725 -2.620 20.612 1.00 18.11 O1-

ATOM 258 H ASP A 106 -7.767 -6.219 20.475 1.00 0.00 H

ATOM 259 HA ASP A 106 -7.785 -5.102 17.801 1.00 0.00 H

ATOM 260 HB2 ASP A 106 -9.382 -3.308 18.252 1.00 0.00 H

ATOM 261 HB3 ASP A 106 -10.210 -4.800 18.390 1.00 0.00 H

ATOM 262 N MET A 107 -6.547 -3.029 18.821 1.00 17.54 N

ATOM 263 CA MET A 107 -5.538 -2.095 19.310 1.00 17.52 C

ATOM 264 H MET A 107 -6.988 -2.788 17.935 1.00 0.00 H

ATOM 265 HA MET A 107 -4.715 -2.691 19.698 1.00 0.00 H

ATOM 266 H01 MET A 107 -5.955 -1.472 20.101 1.00 17.52 H

ATOM 267 H02 MET A 107 -5.198 -1.436 18.511 1.00 17.52 H

ATOM 268 CA THR A 131 -4.298 -12.736 17.563 1.00 17.20 C

ATOM 269 C THR A 131 -5.217 -11.825 16.721 1.00 17.33 C

ATOM 270 O THR A 131 -5.965 -12.343 15.892 1.00 18.09 O

ATOM 271 HA THR A 131 -4.333 -13.735 17.124 1.00 0.00 H

ATOM 272 H01 THR A 131 -4.650 -12.770 18.594 1.00 17.20 H

ATOM 273 H02 THR A 131 -3.277 -12.354 17.565 1.00 17.20 H

ATOM 274 N MET A 132 -5.083 -10.499 16.879 1.00 17.52 N

ATOM 275 CA MET A 132 -5.810 -9.490 16.104 1.00 16.83 C

ATOM 276 C MET A 132 -5.614 -9.654 14.582 1.00 17.25 C

ATOM 277 O MET A 132 -6.602 -9.656 13.850 1.00 18.87 O

ATOM 278 CB MET A 132 -5.425 -8.083 16.609 1.00 16.57 C

ATOM 279 CG MET A 132 -6.046 -6.902 15.844 1.00 17.94 C

ATOM 280 SD MET A 132 -5.332 -5.309 16.317 1.00 18.93 S

ATOM 281 CE MET A 132 -3.792 -5.347 15.358 1.00 21.16 C

ATOM 282 H MET A 132 -4.411 -10.143 17.553 1.00 0.00 H

ATOM 283 HA MET A 132 -6.873 -9.636 16.308 1.00 0.00 H

ATOM 284 HB2 MET A 132 -5.709 -7.996 17.658 1.00 0.00 H

ATOM 285 HB3 MET A 132 -4.343 -7.975 16.592 1.00 0.00 H

ATOM 286 HG2 MET A 132 -5.927 -7.000 14.765 1.00 0.00 H

ATOM 287 HG3 MET A 132 -7.118 -6.871 16.031 1.00 0.00 H

ATOM 288 HE1 MET A 132 -3.209 -4.443 15.532 1.00 0.00 H

ATOM 289 HE2 MET A 132 -3.177 -6.206 15.629 1.00 0.00 H

ATOM 290 HE3 MET A 132 -4.016 -5.407 14.293 1.00 0.00 H

ATOM 291 N PHE A 133 -4.364 -9.831 14.132 1.00 18.62 N

ATOM 292 CA PHE A 133 -4.053 -10.083 12.721 1.00 18.70 C

ATOM 293 H PHE A 133 -3.582 -9.769 14.778 1.00 0.00 H

ATOM 294 HA PHE A 133 -4.542 -9.308 12.129 1.00 0.00 H

ATOM 295 H01 PHE A 133 -4.425 -11.065 12.428 1.00 18.70 H

ATOM 296 H02 PHE A 133 -2.975 -10.063 12.559 1.00 18.70 H

ATOM 297 CA THR A 134 -4.905 -13.844 12.753 1.00 19.70 C

ATOM 298 C THR A 134 -6.440 -13.946 12.580 1.00 18.33 C

ATOM 299 O THR A 134 -6.889 -14.612 11.648 1.00 19.96 O

ATOM 300 HA THR A 134 -4.456 -14.136 11.801 1.00 0.00 H

ATOM 301 H01 THR A 134 -4.572 -14.514 13.546 1.00 19.70 H

ATOM 302 H02 THR A 134 -4.611 -12.831 13.027 1.00 19.70 H

ATOM 303 N ASN A 135 -7.214 -13.250 13.428 1.00 18.27 N

ATOM 304 CA ASN A 135 -8.686 -13.206 13.366 1.00 18.27 C

ATOM 305 C ASN A 135 -9.189 -12.653 12.024 1.00 18.22 C

ATOM 306 O ASN A 135 -10.092 -13.239 11.423 1.00 19.16 O

ATOM 307 CB ASN A 135 -9.264 -12.353 14.519 1.00 17.33 C

ATOM 308 CG ASN A 135 -9.111 -12.949 15.917 1.00 18.25 C

ATOM 309 ND2 ASN A 135 -9.380 -12.146 16.936 1.00 19.11 N

ATOM 310 OD1 ASN A 135 -8.781 -14.120 16.095 1.00 19.88 O

ATOM 311 H ASN A 135 -6.779 -12.746 14.197 1.00 0.00 H

ATOM 312 HA ASN A 135 -9.023 -14.238 13.477 1.00 0.00 H

ATOM 313 HB2 ASN A 135 -8.834 -11.350 14.499 1.00 0.00 H

ATOM 314 HB3 ASN A 135 -10.337 -12.222 14.360 1.00 0.00 H

ATOM 315 HD21 ASN A 135 -9.741 -11.200 16.772 1.00 0.00 H

ATOM 316 HD22 ASN A 135 -9.328 -12.498 17.890 1.00 0.00 H

ATOM 317 N CYS A 136 -8.540 -11.579 11.553 1.00 18.67 N

ATOM 318 CA CYS A 136 -8.819 -10.925 10.279 1.00 20.27 C

ATOM 319 C CYS A 136 -8.624 -11.894 9.095 1.00 20.74 C

ATOM 320 O CYS A 136 -9.497 -11.984 8.229 1.00 22.22 O

ATOM 321 CB CYS A 136 -8.007 -9.617 10.133 1.00 20.20 C

ATOM 322 SG CYS A 136 -8.408 -8.699 8.619 1.00 21.89 S

ATOM 323 H CYS A 136 -7.804 -11.172 12.115 1.00 0.00 H

ATOM 324 HA CYS A 136 -9.875 -10.652 10.297 1.00 0.00 H

ATOM 325 HB2 CYS A 136 -8.200 -8.959 10.979 1.00 0.00 H

ATOM 326 HB3 CYS A 136 -6.937 -9.822 10.133 1.00 0.00 H

ATOM 327 HG CYS A 136 -7.611 -7.653 8.863 1.00 0.00 H

ATOM 328 N TYR A 137 -7.515 -12.647 9.112 1.00 20.34 N

ATOM 329 CA TYR A 137 -7.177 -13.638 8.090 1.00 21.30 C

ATOM 330 H TYR A 137 -6.839 -12.514 9.854 1.00 0.00 H

ATOM 331 H01 TYR A 137 -7.245 -13.181 7.103 1.00 21.30 H

ATOM 332 H02 TYR A 137 -7.872 -14.476 8.152 1.00 21.30 H

ATOM 333 H03 TYR A 137 -6.161 -13.996 8.254 1.00 21.30 H

ATOM 334 CA ILE A 138 -9.531 -16.364 9.465 1.00 22.28 C

ATOM 335 C ILE A 138 -10.939 -16.011 8.941 1.00 22.55 C

ATOM 336 O ILE A 138 -11.509 -16.803 8.188 1.00 23.90 O

ATOM 337 HA ILE A 138 -9.171 -17.192 8.850 1.00 0.00 H

ATOM 338 H01 ILE A 138 -9.582 -16.665 10.511 1.00 22.28 H

ATOM 339 H02 ILE A 138 -8.861 -15.507 9.404 1.00 22.28 H

ATOM 340 N TYR A 139 -11.486 -14.857 9.353 1.00 22.08 N

ATOM 341 CA TYR A 139 -12.862 -14.483 9.021 1.00 22.55 C

ATOM 342 C TYR A 139 -13.036 -14.055 7.550 1.00 23.58 C

ATOM 343 O TYR A 139 -13.950 -14.537 6.878 1.00 23.96 O

ATOM 344 CB TYR A 139 -13.397 -13.415 10.005 1.00 21.79 C

ATOM 345 CG TYR A 139 -14.852 -13.038 9.759 1.00 22.15 C

ATOM 346 CD1 TYR A 139 -15.879 -13.940 10.106 1.00 22.76 C

ATOM 347 CD2 TYR A 139 -15.184 -11.818 9.130 1.00 22.78 C

ATOM 348 CE1 TYR A 139 -17.220 -13.637 9.800 1.00 23.42 C

ATOM 349 CE2 TYR A 139 -16.528 -11.514 8.833 1.00 24.54 C

ATOM 350 CZ TYR A 139 -17.548 -12.429 9.158 1.00 24.79 C

ATOM 351 OH TYR A 139 -18.846 -12.149 8.840 1.00 27.26 O

ATOM 352 H TYR A 139 -10.970 -14.246 9.982 1.00 0.00 H

ATOM 353 HA TYR A 139 -13.480 -15.372 9.166 1.00 0.00 H

ATOM 354 HB2 TYR A 139 -13.312 -13.780 11.030 1.00 0.00 H

ATOM 355 HB3 TYR A 139 -12.780 -12.516 9.955 1.00 0.00 H

ATOM 356 HD1 TYR A 139 -15.642 -14.877 10.589 1.00 0.00 H

ATOM 357 HD2 TYR A 139 -14.408 -11.116 8.861 1.00 0.00 H

ATOM 358 HE1 TYR A 139 -17.997 -14.339 10.059 1.00 0.00 H

ATOM 359 HE2 TYR A 139 -16.771 -10.580 8.346 1.00 0.00 H

ATOM 360 HH TYR A 139 -19.496 -12.864 9.072 1.00 0.00 H

ATOM 361 N ASN A 140 -12.181 -13.141 7.081 1.00 22.52 N

ATOM 362 CA ASN A 140 -12.339 -12.463 5.788 1.00 25.02 C

ATOM 363 CB ASN A 140 -11.704 -11.059 5.854 1.00 23.21 C

ATOM 364 CG ASN A 140 -12.326 -10.175 6.934 1.00 21.77 C

ATOM 365 ND2 ASN A 140 -11.594 -9.944 8.014 1.00 21.24 N

ATOM 366 OD1 ASN A 140 -13.458 -9.714 6.807 1.00 23.61 O

ATOM 367 H ASN A 140 -11.390 -12.853 7.646 1.00 0.00 H

ATOM 368 HB2 ASN A 140 -10.631 -11.146 6.016 1.00 0.00 H

ATOM 369 HB3 ASN A 140 -11.823 -10.550 4.896 1.00 0.00 H

ATOM 370 HD21 ASN A 140 -10.674 -10.364 8.092 1.00 0.00 H

ATOM 371 HD22 ASN A 140 -11.916 -9.359 8.789 1.00 0.00 H

ATOM 372 H01 ASN A 140 -13.400 -12.371 5.554 1.00 25.02 H

ATOM 373 H02 ASN A 140 -11.843 -13.046 5.013 1.00 25.02 H

ATOM 374 CA ASP A 144 -10.325 -8.345 0.788 1.00 35.24 C

ATOM 375 C ASP A 144 -9.467 -7.185 1.314 1.00 31.77 C

ATOM 376 O ASP A 144 -8.412 -7.434 1.898 1.00 30.16 O

ATOM 377 CB ASP A 144 -11.300 -8.787 1.916 1.00 38.70 C

ATOM 378 CG ASP A 144 -12.409 -9.759 1.519 1.00 41.15 C

ATOM 379 OD1 ASP A 144 -12.799 -9.774 0.332 1.00 42.96 O

ATOM 380 OD2 ASP A 144 -12.985 -10.387 2.434 1.00 41.61 O1-

ATOM 381 HB2 ASP A 144 -10.711 -9.253 2.708 1.00 0.00 H

ATOM 382 HB3 ASP A 144 -11.781 -7.909 2.350 1.00 0.00 H

ATOM 383 H01 ASP A 144 -9.683 -9.180 0.507 1.00 35.24 H

ATOM 384 H02 ASP A 144 -10.888 -8.026 -0.089 1.00 35.24 H

ATOM 385 N ASP A 145 -9.938 -5.943 1.130 1.00 29.93 N

ATOM 386 CA ASP A 145 -9.229 -4.712 1.524 1.00 28.44 C

ATOM 387 C ASP A 145 -8.774 -4.710 2.988 1.00 26.28 C

ATOM 388 O ASP A 145 -7.641 -4.330 3.266 1.00 27.09 O

ATOM 389 H ASP A 145 -10.793 -5.818 0.609 1.00 0.00 H

ATOM 390 H01 ASP A 145 -9.896 -3.864 1.368 1.00 28.44 H

ATOM 391 H02 ASP A 145 -8.331 -4.649 0.909 1.00 28.44 H

ATOM 392 N ILE A 146 -9.637 -5.189 3.888 1.00 24.59 N

ATOM 393 CA ILE A 146 -9.355 -5.283 5.318 1.00 24.69 C

ATOM 394 C ILE A 146 -8.196 -6.260 5.657 1.00 23.55 C

ATOM 395 O ILE A 146 -7.437 -5.999 6.591 1.00 23.63 O

ATOM 396 CB ILE A 146 -10.653 -5.651 6.092 1.00 23.87 C

ATOM 397 CG1 ILE A 146 -10.539 -5.467 7.618 1.00 24.18 C

ATOM 398 CG2 ILE A 146 -11.188 -7.060 5.770 1.00 25.24 C

ATOM 399 CD1 ILE A 146 -10.224 -4.033 8.059 1.00 24.07 C

ATOM 400 H ILE A 146 -10.543 -5.506 3.580 1.00 0.00 H

ATOM 401 HA ILE A 146 -9.041 -4.288 5.630 1.00 0.00 H

ATOM 402 HB ILE A 146 -11.421 -4.950 5.763 1.00 0.00 H

ATOM 403 HG12 ILE A 146 -11.487 -5.747 8.071 1.00 0.00 H

ATOM 404 HG13 ILE A 146 -9.800 -6.149 8.037 1.00 0.00 H

ATOM 405 HG21 ILE A 146 -12.164 -7.214 6.230 1.00 0.00 H

ATOM 406 HG22 ILE A 146 -11.310 -7.218 4.700 1.00 0.00 H

ATOM 407 HG23 ILE A 146 -10.527 -7.839 6.150 1.00 0.00 H

ATOM 408 HD11 ILE A 146 -10.427 -3.923 9.123 1.00 0.00 H

ATOM 409 HD12 ILE A 146 -9.176 -3.783 7.899 1.00 0.00 H

ATOM 410 HD13 ILE A 146 -10.834 -3.302 7.528 1.00 0.00 H

ATOM 411 N VAL A 147 -8.015 -7.317 4.848 1.00 24.32 N

ATOM 412 CA VAL A 147 -6.880 -8.233 4.950 1.00 25.29 C

ATOM 413 H VAL A 147 -8.599 -7.420 4.028 1.00 0.00 H

ATOM 414 HA VAL A 147 -6.751 -8.490 6.004 1.00 0.00 H

ATOM 415 H01 VAL A 147 -5.978 -7.751 4.572 1.00 25.29 H

ATOM 416 H02 VAL A 147 -7.062 -9.131 4.359 1.00 25.29 H

ATOM 417 CA LEU A 148 -4.527 -5.976 2.875 1.00 27.61 C

ATOM 418 C LEU A 148 -4.092 -4.888 3.882 1.00 28.09 C

ATOM 419 O LEU A 148 -2.894 -4.665 4.065 1.00 28.07 O

ATOM 420 HA LEU A 148 -3.680 -6.651 2.740 1.00 0.00 H

ATOM 421 H01 LEU A 148 -4.795 -5.518 1.923 1.00 27.61 H

ATOM 422 H02 LEU A 148 -5.397 -6.518 3.245 1.00 27.61 H

ATOM 423 N MET A 149 -5.075 -4.265 4.549 1.00 27.25 N

ATOM 424 CA MET A 149 -4.893 -3.337 5.666 1.00 27.72 C

ATOM 425 C MET A 149 -4.189 -4.009 6.862 1.00 26.17 C

ATOM 426 O MET A 149 -3.208 -3.459 7.364 1.00 26.78 O

ATOM 427 CB MET A 149 -6.253 -2.721 6.069 1.00 28.73 C

ATOM 428 CG MET A 149 -6.833 -1.760 5.015 1.00 31.61 C

ATOM 429 SD MET A 149 -8.580 -1.321 5.253 1.00 34.10 S

ATOM 430 CE MET A 149 -8.405 0.103 6.359 1.00 34.24 C

ATOM 431 H MET A 149 -6.035 -4.470 4.281 1.00 0.00 H

ATOM 432 HA MET A 149 -4.242 -2.531 5.323 1.00 0.00 H

ATOM 433 HB2 MET A 149 -6.970 -3.511 6.277 1.00 0.00 H

ATOM 434 HB3 MET A 149 -6.149 -2.190 7.014 1.00 0.00 H

ATOM 435 HG2 MET A 149 -6.246 -0.846 4.984 1.00 0.00 H

ATOM 436 HG3 MET A 149 -6.733 -2.181 4.017 1.00 0.00 H

ATOM 437 HE1 MET A 149 -9.384 0.498 6.630 1.00 0.00 H

ATOM 438 HE2 MET A 149 -7.880 -0.181 7.271 1.00 0.00 H

ATOM 439 HE3 MET A 149 -7.849 0.898 5.863 1.00 0.00 H

ATOM 440 N ALA A 150 -4.653 -5.204 7.258 1.00 24.20 N

ATOM 441 CA ALA A 150 -4.034 -6.021 8.304 1.00 24.44 C

ATOM 442 C ALA A 150 -2.591 -6.449 7.975 1.00 25.72 C

ATOM 443 O ALA A 150 -1.729 -6.318 8.840 1.00 25.70 O

ATOM 444 CB ALA A 150 -4.916 -7.236 8.631 1.00 25.86 C

ATOM 445 H ALA A 150 -5.487 -5.585 6.820 1.00 0.00 H

ATOM 446 HA ALA A 150 -3.988 -5.403 9.204 1.00 0.00 H

ATOM 447 HB1 ALA A 150 -4.423 -7.901 9.342 1.00 0.00 H

ATOM 448 HB2 ALA A 150 -5.858 -6.927 9.079 1.00 0.00 H

ATOM 449 HB3 ALA A 150 -5.147 -7.820 7.742 1.00 0.00 H

ATOM 450 N GLU A 151 -2.332 -6.890 6.734 1.00 27.29 N

ATOM 451 CA GLU A 151 -0.997 -7.256 6.246 0.83 27.47 C

ATOM 452 H GLU A 151 -3.108 -7.032 6.091 1.00 0.00 H

ATOM 453 H01 GLU A 151 -0.708 -6.584 5.438 0.83 27.47 H

ATOM 454 H02 GLU A 151 -1.013 -8.282 5.878 0.83 27.47 H

ATOM 455 H03 GLU A 151 -0.278 -7.174 7.061 0.83 27.47 H

TER

END

**BRD9 Protein**

ATOM 1 CA GLY A 43 -8.897 2.518 11.717 1.00 9.85 C

ATOM 2 C GLY A 43 -9.787 1.269 11.735 1.00 9.81 C

ATOM 3 O GLY A 43 -10.716 1.196 12.539 1.00 9.59 O

ATOM 4 HA2 GLY A 43 -8.016 2.339 12.333 1.00 0.00 H

ATOM 5 HA3 GLY A 43 -9.424 3.368 12.153 1.00 0.00 H

ATOM 6 H01 GLY A 43 -8.627 2.729 10.682 1.00 9.85 H

ATOM 7 N PHE A 44 -9.524 0.298 10.852 1.00 9.28 N

ATOM 8 CA PHE A 44 -10.216 -0.990 10.817 1.00 9.25 C

ATOM 9 C PHE A 44 -9.930 -1.824 12.082 1.00 9.12 C

ATOM 10 O PHE A 44 -10.834 -2.487 12.587 1.00 9.48 O

ATOM 11 CB PHE A 44 -9.814 -1.777 9.550 1.00 10.70 C

ATOM 12 CG PHE A 44 -10.098 -1.125 8.200 1.00 11.15 C

ATOM 13 CD1 PHE A 44 -11.311 -0.446 7.944 1.00 12.02 C

ATOM 14 CD2 PHE A 44 -9.212 -1.352 7.125 1.00 12.16 C

ATOM 15 CE1 PHE A 44 -11.580 0.042 6.670 1.00 13.60 C

ATOM 16 CE2 PHE A 44 -9.496 -0.854 5.860 1.00 13.62 C

ATOM 17 CZ PHE A 44 -10.676 -0.157 5.635 1.00 14.06 C

ATOM 18 H PHE A 44 -8.651 0.363 10.325 1.00 0.00 H

ATOM 19 HA PHE A 44 -11.291 -0.802 10.792 1.00 0.00 H

ATOM 20 HB2 PHE A 44 -8.744 -1.990 9.606 1.00 0.00 H

ATOM 21 HB3 PHE A 44 -10.309 -2.750 9.556 1.00 0.00 H

ATOM 22 HD1 PHE A 44 -12.040 -0.300 8.728 1.00 0.00 H

ATOM 23 HD2 PHE A 44 -8.303 -1.911 7.285 1.00 0.00 H

ATOM 24 HE1 PHE A 44 -12.500 0.576 6.483 1.00 0.00 H

ATOM 25 HE2 PHE A 44 -8.803 -1.019 5.048 1.00 0.00 H

ATOM 26 HZ PHE A 44 -10.893 0.226 4.648 1.00 0.00 H

ATOM 27 N PHE A 45 -8.702 -1.746 12.604 1.00 8.38 N

ATOM 28 CA PHE A 45 -8.236 -2.494 13.770 1.00 7.13 C

ATOM 29 C PHE A 45 -8.329 -1.653 15.062 1.00 7.66 C

ATOM 30 O PHE A 45 -7.946 -2.133 16.129 1.00 8.52 O

ATOM 31 CB PHE A 45 -6.802 -2.997 13.496 1.00 8.90 C

ATOM 32 CG PHE A 45 -6.677 -3.983 12.343 1.00 9.52 C

ATOM 33 CD1 PHE A 45 -7.015 -5.338 12.535 1.00 9.73 C

ATOM 34 CD2 PHE A 45 -6.389 -3.533 11.036 1.00 11.65 C

ATOM 35 CE1 PHE A 45 -6.990 -6.221 11.465 1.00 10.43 C

ATOM 36 CE2 PHE A 45 -6.382 -4.429 9.975 1.00 12.65 C

ATOM 37 CZ PHE A 45 -6.678 -5.768 10.189 1.00 12.00 C

ATOM 38 H PHE A 45 -7.999 -1.106 12.201 1.00 0.00 H

ATOM 39 HA PHE A 45 -8.852 -3.379 13.920 1.00 0.00 H

ATOM 40 HB2 PHE A 45 -6.141 -2.153 13.306 1.00 0.00 H

ATOM 41 HB3 PHE A 45 -6.412 -3.476 14.394 1.00 0.00 H

ATOM 42 HD1 PHE A 45 -7.301 -5.689 13.516 1.00 0.00 H

ATOM 43 HD2 PHE A 45 -6.180 -2.489 10.856 1.00 0.00 H

ATOM 44 HE1 PHE A 45 -7.235 -7.259 11.631 1.00 0.00 H

ATOM 45 HE2 PHE A 45 -6.155 -4.083 8.978 1.00 0.00 H

ATOM 46 HZ PHE A 45 -6.682 -6.454 9.355 1.00 0.00 H

ATOM 47 N ALA A 46 -8.821 -0.407 14.960 1.00 7.56 N

ATOM 48 CA ALA A 46 -8.859 0.579 16.042 1.00 8.87 C

ATOM 49 C ALA A 46 -9.856 0.271 17.175 1.00 7.25 C

ATOM 50 O ALA A 46 -9.624 0.710 18.301 1.00 8.35 O

ATOM 51 CB ALA A 46 -9.150 1.969 15.457 1.00 9.84 C

ATOM 52 H ALA A 46 -9.113 -0.100 14.041 1.00 0.00 H

ATOM 53 HA ALA A 46 -7.864 0.620 16.483 1.00 0.00 H

ATOM 54 HB1 ALA A 46 -9.138 2.730 16.239 1.00 0.00 H

ATOM 55 HB2 ALA A 46 -8.398 2.252 14.721 1.00 0.00 H

ATOM 56 HB3 ALA A 46 -10.129 2.009 14.982 1.00 0.00 H

ATOM 57 N PHE A 47 -10.951 -0.429 16.859 1.00 8.21 N

ATOM 58 CA PHE A 47 -12.055 -0.737 17.776 1.00 8.62 C

ATOM 59 C PHE A 47 -12.494 -2.200 17.568 1.00 9.10 C

ATOM 60 O PHE A 47 -12.167 -2.755 16.515 1.00 9.54 O

ATOM 61 CB PHE A 47 -13.244 0.215 17.491 1.00 8.80 C

ATOM 62 CG PHE A 47 -12.983 1.691 17.731 1.00 9.00 C

ATOM 63 CD1 PHE A 47 -12.913 2.196 19.046 1.00 9.47 C

ATOM 64 CD2 PHE A 47 -12.674 2.543 16.650 1.00 9.50 C

ATOM 65 CE1 PHE A 47 -12.589 3.530 19.260 1.00 10.52 C

ATOM 66 CE2 PHE A 47 -12.353 3.873 16.884 1.00 10.66 C

ATOM 67 CZ PHE A 47 -12.316 4.365 18.183 1.00 10.10 C

ATOM 68 H PHE A 47 -11.029 -0.837 15.937 1.00 0.00 H

ATOM 69 HA PHE A 47 -11.714 -0.614 18.803 1.00 0.00 H

ATOM 70 HB2 PHE A 47 -13.590 0.077 16.465 1.00 0.00 H

ATOM 71 HB3 PHE A 47 -14.095 -0.057 18.113 1.00 0.00 H

ATOM 72 HD1 PHE A 47 -13.105 1.550 19.892 1.00 0.00 H

ATOM 73 HD2 PHE A 47 -12.679 2.165 15.636 1.00 0.00 H

ATOM 74 HE1 PHE A 47 -12.543 3.918 20.268 1.00 0.00 H

ATOM 75 HE2 PHE A 47 -12.123 4.526 16.054 1.00 0.00 H

ATOM 76 HZ PHE A 47 -12.061 5.402 18.358 1.00 0.00 H

ATOM 77 N PRO A 48 -13.270 -2.793 18.509 1.00 9.72 N

ATOM 78 CA PRO A 48 -13.865 -4.131 18.311 1.00 10.70 C

ATOM 79 C PRO A 48 -14.781 -4.205 17.074 1.00 10.40 C

ATOM 80 O PRO A 48 -15.643 -3.337 16.911 1.00 10.08 O

ATOM 81 CB PRO A 48 -14.659 -4.384 19.609 1.00 10.86 C

ATOM 82 CG PRO A 48 -14.040 -3.463 20.645 1.00 10.70 C

ATOM 83 CD PRO A 48 -13.625 -2.250 19.825 1.00 10.44 C

ATOM 84 HA PRO A 48 -13.043 -4.846 18.231 1.00 0.00 H

ATOM 85 HB2 PRO A 48 -15.711 -4.119 19.484 1.00 0.00 H

ATOM 86 HB3 PRO A 48 -14.626 -5.430 19.917 1.00 0.00 H

ATOM 87 HG2 PRO A 48 -14.710 -3.221 21.471 1.00 0.00 H

ATOM 88 HG3 PRO A 48 -13.153 -3.936 21.067 1.00 0.00 H

ATOM 89 HD2 PRO A 48 -14.467 -1.567 19.719 1.00 0.00 H

ATOM 90 HD3 PRO A 48 -12.809 -1.723 20.318 1.00 0.00 H

ATOM 91 N VAL A 49 -14.597 -5.234 16.238 1.00 10.76 N

ATOM 92 CA VAL A 49 -15.485 -5.532 15.114 1.00 11.59 C

ATOM 93 C VAL A 49 -16.854 -5.999 15.661 1.00 11.82 C

ATOM 94 O VAL A 49 -16.897 -6.873 16.532 1.00 12.86 O

ATOM 95 CB VAL A 49 -14.923 -6.664 14.203 1.00 12.24 C

ATOM 96 CG1 VAL A 49 -15.766 -6.880 12.933 1.00 13.51 C

ATOM 97 CG2 VAL A 49 -13.462 -6.429 13.794 1.00 12.01 C

ATOM 98 H VAL A 49 -13.867 -5.909 16.411 1.00 0.00 H

ATOM 99 HA VAL A 49 -15.612 -4.624 14.520 1.00 0.00 H

ATOM 100 HB VAL A 49 -14.944 -7.603 14.759 1.00 0.00 H

ATOM 101 HG11 VAL A 49 -15.339 -7.664 12.307 1.00 0.00 H

ATOM 102 HG12 VAL A 49 -16.783 -7.186 13.173 1.00 0.00 H

ATOM 103 HG13 VAL A 49 -15.822 -5.973 12.330 1.00 0.00 H

ATOM 104 HG21 VAL A 49 -13.115 -7.200 13.105 1.00 0.00 H

ATOM 105 HG22 VAL A 49 -13.337 -5.464 13.304 1.00 0.00 H

ATOM 106 HG23 VAL A 49 -12.792 -6.459 14.653 1.00 0.00 H

ATOM 107 N THR A 50 -17.939 -5.392 15.176 1.00 12.37 N

ATOM 108 CA THR A 50 -19.300 -5.733 15.569 1.00 13.75 C

ATOM 109 C THR A 50 -19.901 -6.712 14.540 1.00 14.22 C

ATOM 110 O THR A 50 -19.504 -6.715 13.369 1.00 14.10 O

ATOM 111 CB THR A 50 -20.167 -4.442 15.626 1.00 14.96 C

ATOM 112 CG2 THR A 50 -19.618 -3.392 16.605 1.00 15.54 C

ATOM 113 OG1 THR A 50 -20.337 -3.824 14.358 1.00 15.74 O

ATOM 114 H THR A 50 -17.872 -4.701 14.444 1.00 0.00 H

ATOM 115 HA THR A 50 -19.313 -6.204 16.554 1.00 0.00 H

ATOM 116 HB THR A 50 -21.162 -4.723 15.976 1.00 0.00 H

ATOM 117 HG1 THR A 50 -20.693 -2.938 14.505 1.00 0.00 H

ATOM 118 HG21 THR A 50 -20.293 -2.540 16.698 1.00 0.00 H

ATOM 119 HG22 THR A 50 -19.498 -3.819 17.601 1.00 0.00 H

ATOM 120 H01 THR A 50 -18.658 -3.066 16.204 1.00 15.54 H

ATOM 121 N ASP A 51 -20.890 -7.502 14.982 1.00 16.30 N

ATOM 122 CA ASP A 51 -21.642 -8.425 14.118 1.00 17.81 C

ATOM 123 H ASP A 51 -21.146 -7.491 15.959 1.00 0.00 H

ATOM 124 H01 ASP A 51 -21.894 -9.325 14.679 1.00 17.81 H

ATOM 125 H02 ASP A 51 -21.033 -8.693 13.255 1.00 17.81 H

ATOM 126 H03 ASP A 51 -22.558 -7.940 13.780 1.00 17.81 H

ATOM 127 CA ALA A 52 -23.501 -5.542 12.356 1.00 19.82 C

ATOM 128 C ALA A 52 -22.597 -5.181 11.160 1.00 20.31 C

ATOM 129 O ALA A 52 -23.082 -5.140 10.029 1.00 21.72 O

ATOM 130 HA ALA A 52 -24.379 -6.060 11.966 1.00 0.00 H

ATOM 131 H01 ALA A 52 -23.806 -4.635 12.878 1.00 19.82 H

ATOM 132 H02 ALA A 52 -22.965 -6.179 13.060 1.00 19.82 H

ATOM 133 N ILE A 53 -21.307 -4.939 11.428 1.00 18.94 N

ATOM 134 CA ILE A 53 -20.290 -4.675 10.412 1.00 18.33 C

ATOM 135 C ILE A 53 -19.858 -5.962 9.679 1.00 17.17 C

ATOM 136 O ILE A 53 -19.794 -5.964 8.449 1.00 17.33 O

ATOM 137 CB ILE A 53 -19.057 -3.936 11.028 1.00 19.33 C

ATOM 138 CG1 ILE A 53 -19.389 -2.457 11.335 1.00 21.01 C

ATOM 139 CG2 ILE A 53 -17.725 -4.034 10.253 1.00 19.49 C

ATOM 140 CD1 ILE A 53 -19.407 -1.530 10.106 1.00 21.80 C

ATOM 141 H ILE A 53 -20.978 -4.985 12.386 1.00 0.00 H

ATOM 142 HA ILE A 53 -20.737 -4.024 9.658 1.00 0.00 H

ATOM 143 HB ILE A 53 -18.861 -4.404 11.994 1.00 0.00 H

ATOM 144 HG12 ILE A 53 -20.347 -2.388 11.853 1.00 0.00 H

ATOM 145 HG13 ILE A 53 -18.652 -2.075 12.044 1.00 0.00 H

ATOM 146 HG21 ILE A 53 -16.964 -3.399 10.706 1.00 0.00 H

ATOM 147 HG22 ILE A 53 -17.324 -5.048 10.255 1.00 0.00 H

ATOM 148 HG23 ILE A 53 -17.843 -3.725 9.216 1.00 0.00 H

ATOM 149 HD11 ILE A 53 -20.066 -0.678 10.272 1.00 0.00 H

ATOM 150 HD12 ILE A 53 -18.410 -1.136 9.903 1.00 0.00 H

ATOM 151 HD13 ILE A 53 -19.747 -2.036 9.203 1.00 0.00 H

ATOM 152 N ALA A 54 -19.594 -7.029 10.440 1.00 16.31 N

ATOM 153 CA ALA A 54 -19.117 -8.313 9.936 1.00 16.57 C

ATOM 154 C ALA A 54 -20.082 -9.420 10.407 1.00 16.29 C

ATOM 155 O ALA A 54 -19.960 -9.848 11.554 1.00 16.17 O

ATOM 156 CB ALA A 54 -17.696 -8.522 10.484 1.00 16.53 C

ATOM 157 H ALA A 54 -19.675 -6.955 11.452 1.00 0.00 H

ATOM 158 HA ALA A 54 -19.031 -8.320 8.850 1.00 0.00 H

ATOM 159 HB1 ALA A 54 -17.296 -9.487 10.186 1.00 0.00 H

ATOM 160 HB2 ALA A 54 -17.011 -7.756 10.119 1.00 0.00 H

ATOM 161 HB3 ALA A 54 -17.682 -8.492 11.572 1.00 0.00 H

ATOM 162 N PRO A 55 -21.042 -9.854 9.554 1.00 17.80 N

ATOM 163 CA PRO A 55 -22.032 -10.897 9.901 1.00 18.25 C

ATOM 164 C PRO A 55 -21.464 -12.171 10.561 1.00 17.68 C

ATOM 165 O PRO A 55 -20.613 -12.841 9.971 1.00 17.94 O

ATOM 166 CB PRO A 55 -22.722 -11.195 8.559 1.00 19.79 C

ATOM 167 CG PRO A 55 -22.651 -9.878 7.806 1.00 20.10 C

ATOM 168 CD PRO A 55 -21.286 -9.333 8.206 1.00 19.20 C

ATOM 169 HA PRO A 55 -22.757 -10.433 10.570 1.00 0.00 H

ATOM 170 HB2 PRO A 55 -22.177 -11.961 8.003 1.00 0.00 H

ATOM 171 HB3 PRO A 55 -23.746 -11.547 8.684 1.00 0.00 H

ATOM 172 HG2 PRO A 55 -22.777 -9.984 6.727 1.00 0.00 H

ATOM 173 HG3 PRO A 55 -23.431 -9.209 8.171 1.00 0.00 H

ATOM 174 HD2 PRO A 55 -20.509 -9.712 7.539 1.00 0.00 H

ATOM 175 HD3 PRO A 55 -21.278 -8.243 8.161 1.00 0.00 H

ATOM 176 N GLY A 56 -21.947 -12.472 11.773 1.00 17.69 N

ATOM 177 CA GLY A 56 -21.634 -13.697 12.512 1.00 17.65 C

ATOM 178 C GLY A 56 -20.222 -13.705 13.119 1.00 16.99 C

ATOM 179 O GLY A 56 -19.745 -14.780 13.485 1.00 17.98 O

ATOM 180 H GLY A 56 -22.617 -11.848 12.199 1.00 0.00 H

ATOM 181 HA2 GLY A 56 -22.357 -13.817 13.320 1.00 0.00 H

ATOM 182 HA3 GLY A 56 -21.748 -14.565 11.860 1.00 0.00 H

ATOM 183 N TYR A 57 -19.549 -12.549 13.227 1.00 15.92 N

ATOM 184 CA TYR A 57 -18.175 -12.415 13.725 1.00 15.32 C

ATOM 185 C TYR A 57 -17.956 -13.053 15.108 1.00 16.40 C

ATOM 186 O TYR A 57 -17.051 -13.876 15.247 1.00 16.40 O

ATOM 187 CB TYR A 57 -17.732 -10.938 13.702 1.00 13.89 C

ATOM 188 CG TYR A 57 -16.250 -10.716 13.927 1.00 13.12 C

ATOM 189 CD1 TYR A 57 -15.387 -10.707 12.814 1.00 13.17 C

ATOM 190 CD2 TYR A 57 -15.725 -10.534 15.225 1.00 12.12 C

ATOM 191 CE1 TYR A 57 -14.008 -10.519 12.994 1.00 12.70 C

ATOM 192 CE2 TYR A 57 -14.338 -10.358 15.405 1.00 11.86 C

ATOM 193 CZ TYR A 57 -13.482 -10.345 14.287 1.00 11.82 C

ATOM 194 OH TYR A 57 -12.143 -10.167 14.450 1.00 11.92 O

ATOM 195 H TYR A 57 -19.994 -11.690 12.928 1.00 0.00 H

ATOM 196 HA TYR A 57 -17.542 -12.958 13.021 1.00 0.00 H

ATOM 197 HB2 TYR A 57 -17.948 -10.525 12.722 1.00 0.00 H

ATOM 198 HB3 TYR A 57 -18.302 -10.342 14.414 1.00 0.00 H

ATOM 199 HD1 TYR A 57 -15.776 -10.845 11.815 1.00 0.00 H

ATOM 200 HD2 TYR A 57 -16.379 -10.539 16.085 1.00 0.00 H

ATOM 201 HE1 TYR A 57 -13.364 -10.520 12.127 1.00 0.00 H

ATOM 202 HE2 TYR A 57 -13.935 -10.229 16.399 1.00 0.00 H

ATOM 203 HH TYR A 57 -11.716 -9.978 13.581 1.00 0.00 H

ATOM 204 N SER A 58 -18.821 -12.732 16.082 1.00 17.11 N

ATOM 205 CA SER A 58 -18.753 -13.265 17.446 1.00 18.80 C

ATOM 206 H SER A 58 -19.538 -12.013 15.903 1.00 0.00 H

ATOM 207 H01 SER A 58 -18.015 -14.066 17.487 1.00 18.80 H

ATOM 208 H02 SER A 58 -19.730 -13.655 17.734 1.00 18.80 H

ATOM 209 H03 SER A 58 -18.463 -12.469 18.132 1.00 18.80 H

ATOM 210 CA PRO A 64 -11.735 -10.906 19.552 1.00 11.12 C

ATOM 211 C PRO A 64 -10.609 -10.072 20.194 1.00 10.14 C

ATOM 212 O PRO A 64 -10.448 -10.106 21.416 1.00 10.79 O

ATOM 213 HA PRO A 64 -11.530 -11.085 18.495 1.00 0.00 H

ATOM 214 H01 PRO A 64 -12.678 -10.368 19.648 1.00 11.12 H

ATOM 215 H02 PRO A 64 -11.794 -11.867 20.064 1.00 11.12 H

ATOM 216 N MET A 65 -9.872 -9.329 19.367 1.00 9.43 N

ATOM 217 CA MET A 65 -8.827 -8.403 19.794 1.00 8.83 C

ATOM 218 C MET A 65 -8.757 -7.220 18.814 1.00 8.56 C

ATOM 219 O MET A 65 -9.239 -7.329 17.685 1.00 9.25 O

ATOM 220 H MET A 65 -10.073 -9.329 18.365 1.00 0.00 H

ATOM 221 H01 MET A 65 -7.867 -8.920 19.810 1.00 8.83 H

ATOM 222 H02 MET A 65 -9.057 -8.033 20.793 1.00 8.83 H

ATOM 223 N ASP A 66 -8.182 -6.104 19.261 1.00 8.47 N

ATOM 224 CA ASP A 66 -8.171 -4.822 18.557 1.00 8.55 C

ATOM 225 C ASP A 66 -7.197 -3.898 19.308 1.00 7.69 C

ATOM 226 O ASP A 66 -6.901 -4.140 20.484 1.00 8.47 O

ATOM 227 CB ASP A 66 -9.568 -4.146 18.470 1.00 9.34 C

ATOM 228 CG ASP A 66 -10.193 -3.790 19.814 1.00 10.04 C

ATOM 229 OD1 ASP A 66 -10.712 -4.688 20.504 1.00 10.86 O

ATOM 230 OD2 ASP A 66 -10.139 -2.600 20.177 1.00 9.70 O1-

ATOM 231 H ASP A 66 -7.774 -6.068 20.189 1.00 0.00 H

ATOM 232 HA ASP A 66 -7.793 -4.980 17.547 1.00 0.00 H

ATOM 233 HB2 ASP A 66 -9.506 -3.235 17.875 1.00 0.00 H

ATOM 234 HB3 ASP A 66 -10.270 -4.764 17.910 1.00 0.00 H

ATOM 235 N PHE A 67 -6.762 -2.826 18.640 1.00 7.97 N

ATOM 236 CA PHE A 67 -5.869 -1.812 19.199 1.00 8.19 C

ATOM 237 H PHE A 67 -7.080 -2.667 17.685 1.00 0.00 H

ATOM 238 H01 PHE A 67 -5.604 -2.084 20.221 1.00 8.19 H

ATOM 239 H02 PHE A 67 -6.371 -0.845 19.199 1.00 8.19 H

ATOM 240 H03 PHE A 67 -4.965 -1.751 18.593 1.00 8.19 H

ATOM 241 CA LEU A 91 -4.185 -12.735 17.642 1.00 8.27 C

ATOM 242 C LEU A 91 -5.161 -11.897 16.796 1.00 9.47 C

ATOM 243 O LEU A 91 -5.923 -12.463 16.017 1.00 9.15 O

ATOM 244 HA LEU A 91 -4.214 -13.756 17.258 1.00 0.00 H

ATOM 245 H01 LEU A 91 -4.491 -12.721 18.688 1.00 8.27 H

ATOM 246 H02 LEU A 91 -3.175 -12.329 17.579 1.00 8.27 H

ATOM 247 N MET A 92 -5.056 -10.568 16.912 1.00 9.23 N

ATOM 248 CA MET A 92 -5.790 -9.589 16.114 1.00 9.18 C

ATOM 249 C MET A 92 -5.603 -9.793 14.593 1.00 9.54 C

ATOM 250 O MET A 92 -6.588 -9.791 13.856 1.00 9.51 O

ATOM 251 CB MET A 92 -5.355 -8.184 16.574 1.00 9.31 C

ATOM 252 CG MET A 92 -6.066 -7.012 15.889 1.00 10.44 C

ATOM 253 SD MET A 92 -5.230 -5.431 16.158 1.00 10.37 S

ATOM 254 CE MET A 92 -3.918 -5.589 14.914 1.00 11.84 C

ATOM 255 H MET A 92 -4.389 -10.193 17.582 1.00 0.00 H

ATOM 256 HA MET A 92 -6.851 -9.714 16.339 1.00 0.00 H

ATOM 257 HB2 MET A 92 -5.523 -8.094 17.646 1.00 0.00 H

ATOM 258 HB3 MET A 92 -4.281 -8.073 16.439 1.00 0.00 H

ATOM 259 HG2 MET A 92 -6.156 -7.164 14.815 1.00 0.00 H

ATOM 260 HG3 MET A 92 -7.080 -6.936 16.272 1.00 0.00 H

ATOM 261 HE1 MET A 92 -3.333 -4.674 14.866 1.00 0.00 H

ATOM 262 HE2 MET A 92 -3.245 -6.412 15.155 1.00 0.00 H

ATOM 263 HE3 MET A 92 -4.345 -5.770 13.928 1.00 0.00 H

ATOM 264 N CYS A 93 -4.356 -9.994 14.152 1.00 8.80 N

ATOM 265 CA CYS A 93 -4.016 -10.249 12.750 1.00 8.15 C

ATOM 266 H CYS A 93 -3.586 -9.975 14.811 1.00 0.00 H

ATOM 267 H01 CYS A 93 -4.142 -9.333 12.173 1.00 8.15 H

ATOM 268 H02 CYS A 93 -4.671 -11.024 12.352 1.00 8.15 H

ATOM 269 H03 CYS A 93 -2.980 -10.580 12.682 1.00 8.15 H

ATOM 270 CA ASP A 94 -4.791 -14.038 12.863 1.00 9.60 C

ATOM 271 C ASP A 94 -6.319 -14.138 12.765 1.00 10.01 C

ATOM 272 O ASP A 94 -6.801 -14.897 11.927 1.00 11.09 O

ATOM 273 HA ASP A 94 -4.418 -14.310 11.874 1.00 0.00 H

ATOM 274 H01 ASP A 94 -4.411 -14.727 13.617 1.00 9.60 H

ATOM 275 H02 ASP A 94 -4.469 -13.039 13.156 1.00 9.60 H

ATOM 276 N ASN A 95 -7.062 -13.369 13.574 1.00 9.69 N

ATOM 277 CA ASN A 95 -8.530 -13.278 13.522 1.00 9.56 C

ATOM 278 C ASN A 95 -9.013 -12.751 12.165 1.00 9.90 C

ATOM 279 O ASN A 95 -9.875 -13.380 11.551 1.00 11.19 O

ATOM 280 CB ASN A 95 -9.087 -12.388 14.657 1.00 10.34 C

ATOM 281 CG ASN A 95 -9.059 -13.013 16.052 1.00 10.43 C

ATOM 282 ND2 ASN A 95 -9.321 -12.204 17.069 1.00 10.81 N

ATOM 283 OD1 ASN A 95 -8.851 -14.213 16.222 1.00 11.21 O

ATOM 284 H ASN A 95 -6.597 -12.833 14.306 1.00 0.00 H

ATOM 285 HA ASN A 95 -8.890 -14.299 13.660 1.00 0.00 H

ATOM 286 HB2 ASN A 95 -8.586 -11.419 14.669 1.00 0.00 H

ATOM 287 HB3 ASN A 95 -10.139 -12.177 14.455 1.00 0.00 H

ATOM 288 HD21 ASN A 95 -9.608 -11.235 16.891 1.00 0.00 H

ATOM 289 HD22 ASN A 95 -9.356 -12.557 18.023 1.00 0.00 H

ATOM 290 N ALA A 96 -8.401 -11.663 11.677 1.00 9.92 N

ATOM 291 CA ALA A 96 -8.708 -11.084 10.370 1.00 10.58 C

ATOM 292 C ALA A 96 -8.413 -12.040 9.206 1.00 11.27 C

ATOM 293 O ALA A 96 -9.216 -12.119 8.276 1.00 12.39 O

ATOM 294 CB ALA A 96 -7.976 -9.750 10.196 1.00 10.41 C

ATOM 295 H ALA A 96 -7.704 -11.190 12.238 1.00 0.00 H

ATOM 296 HA ALA A 96 -9.780 -10.886 10.339 1.00 0.00 H

ATOM 297 HB1 ALA A 96 -8.063 -9.372 9.176 1.00 0.00 H

ATOM 298 HB2 ALA A 96 -8.405 -8.997 10.854 1.00 0.00 H

ATOM 299 HB3 ALA A 96 -6.917 -9.841 10.433 1.00 0.00 H

ATOM 300 N MET A 97 -7.308 -12.789 9.300 1.00 10.66 N

ATOM 301 CA MET A 97 -6.961 -13.831 8.334 1.00 11.55 C

ATOM 302 H MET A 97 -6.681 -12.645 10.085 1.00 0.00 H

ATOM 303 HA MET A 97 -7.183 -13.437 7.345 1.00 0.00 H

ATOM 304 H01 MET A 97 -7.552 -14.727 8.521 1.00 11.55 H

ATOM 305 H02 MET A 97 -5.908 -14.101 8.416 1.00 11.55 H

ATOM 306 CA THR A 98 -9.423 -16.416 9.820 1.00 14.49 C

ATOM 307 C THR A 98 -10.841 -16.071 9.321 1.00 14.69 C

ATOM 308 O THR A 98 -11.444 -16.892 8.626 1.00 15.40 O

ATOM 309 HA THR A 98 -9.079 -17.287 9.257 1.00 0.00 H

ATOM 310 H01 THR A 98 -9.448 -16.647 10.885 1.00 14.49 H

ATOM 311 H02 THR A 98 -8.749 -15.573 9.672 1.00 14.49 H

ATOM 312 N TYR A 99 -11.354 -14.881 9.669 1.00 13.62 N

ATOM 313 CA TYR A 99 -12.702 -14.451 9.296 1.00 13.33 C

ATOM 314 C TYR A 99 -12.815 -14.127 7.793 1.00 13.50 C

ATOM 315 O TYR A 99 -13.739 -14.609 7.134 1.00 15.25 O

ATOM 316 CB TYR A 99 -13.173 -13.275 10.189 1.00 13.99 C

ATOM 317 CG TYR A 99 -14.586 -12.793 9.882 1.00 14.88 C

ATOM 318 CD1 TYR A 99 -15.701 -13.445 10.450 1.00 15.71 C

ATOM 319 CD2 TYR A 99 -14.790 -11.731 8.977 1.00 14.85 C

ATOM 320 CE1 TYR A 99 -17.007 -13.065 10.081 1.00 15.62 C

ATOM 321 CE2 TYR A 99 -16.093 -11.367 8.591 1.00 15.22 C

ATOM 322 CZ TYR A 99 -17.202 -12.044 9.131 1.00 15.62 C

ATOM 323 OH TYR A 99 -18.460 -11.707 8.726 1.00 16.93 O

ATOM 324 H TYR A 99 -10.817 -14.249 10.261 1.00 0.00 H

ATOM 325 HA TYR A 99 -13.378 -15.286 9.495 1.00 0.00 H

ATOM 326 HB2 TYR A 99 -13.136 -13.569 11.238 1.00 0.00 H

ATOM 327 HB3 TYR A 99 -12.486 -12.432 10.093 1.00 0.00 H

ATOM 328 HD1 TYR A 99 -15.560 -14.251 11.156 1.00 0.00 H

ATOM 329 HD2 TYR A 99 -13.945 -11.216 8.544 1.00 0.00 H

ATOM 330 HE1 TYR A 99 -17.854 -13.575 10.516 1.00 0.00 H

ATOM 331 HE2 TYR A 99 -16.235 -10.572 7.873 1.00 0.00 H

ATOM 332 HH TYR A 99 -19.161 -12.223 9.168 1.00 0.00 H

ATOM 333 N ASN A 100 -11.891 -13.306 7.278 1.00 13.02 N

ATOM 334 CA ASN A 100 -11.965 -12.777 5.914 1.00 13.70 C

ATOM 335 CB ASN A 100 -11.297 -11.388 5.801 1.00 13.65 C

ATOM 336 CG ASN A 100 -11.854 -10.335 6.756 1.00 12.30 C

ATOM 337 ND2 ASN A 100 -11.136 -10.082 7.839 1.00 12.53 N

ATOM 338 OD1 ASN A 100 -12.909 -9.751 6.521 1.00 12.98 O

ATOM 339 H ASN A 100 -11.096 -13.016 7.835 1.00 0.00 H

ATOM 340 HB2 ASN A 100 -10.217 -11.467 5.926 1.00 0.00 H

ATOM 341 HB3 ASN A 100 -11.450 -11.008 4.793 1.00 0.00 H

ATOM 342 HD21 ASN A 100 -10.306 -10.639 8.023 1.00 0.00 H

ATOM 343 HD22 ASN A 100 -11.412 -9.374 8.528 1.00 0.00 H

ATOM 344 H01 ASN A 100 -13.012 -12.690 5.625 1.00 13.70 H

ATOM 345 H02 ASN A 100 -11.433 -13.463 5.255 1.00 13.70 H

ATOM 346 CA THR A 104 -9.976 -8.583 0.922 0.52 17.81 C

ATOM 347 C THR A 104 -8.882 -7.640 1.456 0.52 17.36 C

ATOM 348 O THR A 104 -7.761 -8.082 1.731 0.52 17.13 O

ATOM 349 CB THR A 104 -10.820 -9.086 2.129 0.52 17.48 C

ATOM 350 CG2 THR A 104 -11.966 -10.025 1.724 0.52 17.65 C

ATOM 351 OG1 THR A 104 -10.030 -9.736 3.112 0.52 17.18 O

ATOM 352 HB THR A 104 -11.270 -8.218 2.614 0.52 0.00 H

ATOM 353 HG1 THR A 104 -9.842 -10.638 2.788 0.52 0.00 H

ATOM 354 HG21 THR A 104 -12.560 -10.319 2.589 0.52 0.00 H

ATOM 355 HG22 THR A 104 -12.637 -9.531 1.021 0.52 0.00 H

ATOM 356 HG23 THR A 104 -11.602 -10.931 1.243 0.52 0.00 H

ATOM 357 H01 THR A 104 -9.517 -9.431 0.414 0.52 17.81 H

ATOM 358 H02 THR A 104 -10.613 -8.055 0.213 0.52 17.81 H

ATOM 359 N VAL A 105 -9.231 -6.357 1.619 1.00 17.11 N

ATOM 360 CA VAL A 105 -8.343 -5.335 2.177 0.70 17.16 C

ATOM 361 C VAL A 105 -7.923 -5.638 3.634 1.00 16.08 C

ATOM 362 O VAL A 105 -6.784 -5.366 4.008 1.00 14.79 O

ATOM 363 CB VAL A 105 -8.997 -3.923 2.107 0.70 18.38 C

ATOM 364 CG1 VAL A 105 -9.243 -3.491 0.650 0.70 19.57 C

ATOM 365 CG2 VAL A 105 -10.286 -3.749 2.940 0.70 18.41 C

ATOM 366 H VAL A 105 -10.155 -6.045 1.361 1.00 0.00 H

ATOM 367 HA VAL A 105 -7.431 -5.324 1.575 0.70 0.00 H

ATOM 368 HB VAL A 105 -8.270 -3.216 2.513 0.70 0.00 H

ATOM 369 HG11 VAL A 105 -9.607 -2.464 0.600 0.70 0.00 H

ATOM 370 HG12 VAL A 105 -8.323 -3.536 0.066 0.70 0.00 H

ATOM 371 HG13 VAL A 105 -9.980 -4.124 0.156 0.70 0.00 H

ATOM 372 HG21 VAL A 105 -10.718 -2.759 2.792 0.70 0.00 H

ATOM 373 HG22 VAL A 105 -11.050 -4.479 2.674 0.70 0.00 H

ATOM 374 H01 VAL A 105 -9.989 -3.891 3.979 0.70 18.41 H

ATOM 375 N TYR A 106 -8.834 -6.250 4.402 1.00 15.34 N

ATOM 376 CA TYR A 106 -8.661 -6.603 5.808 1.00 13.88 C

ATOM 377 C TYR A 106 -7.616 -7.712 5.996 1.00 13.62 C

ATOM 378 O TYR A 106 -6.746 -7.573 6.854 1.00 13.17 O

ATOM 379 CB TYR A 106 -10.025 -7.000 6.410 1.00 14.62 C

ATOM 380 CG TYR A 106 -11.143 -6.019 6.094 1.00 14.62 C

ATOM 381 CD1 TYR A 106 -11.120 -4.722 6.647 1.00 14.51 C

ATOM 382 CD2 TYR A 106 -12.181 -6.382 5.210 1.00 15.58 C

ATOM 383 CE1 TYR A 106 -12.109 -3.785 6.293 1.00 15.48 C

ATOM 384 CE2 TYR A 106 -13.173 -5.444 4.861 1.00 16.53 C

ATOM 385 CZ TYR A 106 -13.130 -4.141 5.392 1.00 16.83 C

ATOM 386 OH TYR A 106 -14.070 -3.224 5.022 1.00 18.12 O

ATOM 387 H TYR A 106 -9.750 -6.424 4.017 1.00 0.00 H

ATOM 388 HA TYR A 106 -8.301 -5.716 6.334 1.00 0.00 H

ATOM 389 HB2 TYR A 106 -10.316 -7.979 6.030 1.00 0.00 H

ATOM 390 HB3 TYR A 106 -9.940 -7.114 7.492 1.00 0.00 H

ATOM 391 HD1 TYR A 106 -10.334 -4.429 7.329 1.00 0.00 H

ATOM 392 HD2 TYR A 106 -12.222 -7.378 4.792 1.00 0.00 H

ATOM 393 HE1 TYR A 106 -12.065 -2.792 6.715 1.00 0.00 H

ATOM 394 HE2 TYR A 106 -13.961 -5.727 4.178 1.00 0.00 H

ATOM 395 HH TYR A 106 -13.897 -2.351 5.396 1.00 0.00 H

ATOM 396 N TYR A 107 -7.675 -8.760 5.157 1.00 13.58 N

ATOM 397 CA TYR A 107 -6.694 -9.849 5.139 1.00 12.92 C

ATOM 398 H TYR A 107 -8.408 -8.801 4.457 1.00 0.00 H

ATOM 399 H01 TYR A 107 -6.452 -10.103 4.107 1.00 12.92 H

ATOM 400 H02 TYR A 107 -7.110 -10.722 5.641 1.00 12.92 H

ATOM 401 H03 TYR A 107 -5.789 -9.531 5.656 1.00 12.92 H

ATOM 402 CA LYS A 108 -4.041 -7.974 3.078 1.00 13.54 C

ATOM 403 C LYS A 108 -3.332 -7.039 4.071 1.00 13.05 C

ATOM 404 O LYS A 108 -2.120 -7.166 4.248 1.00 13.61 O

ATOM 405 H01 LYS A 108 -4.287 -8.913 3.574 1.00 13.54 H

ATOM 406 H02 LYS A 108 -3.384 -8.171 2.231 1.00 13.54 H

ATOM 407 H03 LYS A 108 -4.957 -7.500 2.724 1.00 13.54 H

ATOM 408 N LEU A 109 -4.096 -6.154 4.728 1.00 12.89 N

ATOM 409 CA LEU A 109 -3.573 -5.260 5.758 1.00 12.59 C

ATOM 410 C LEU A 109 -3.093 -6.034 6.998 1.00 11.40 C

ATOM 411 O LEU A 109 -1.982 -5.771 7.449 1.00 11.73 O

ATOM 412 CB LEU A 109 -4.596 -4.150 6.097 1.00 11.96 C

ATOM 413 CG LEU A 109 -4.018 -2.972 6.921 1.00 12.94 C

ATOM 414 CD1 LEU A 109 -2.891 -2.233 6.167 1.00 14.26 C

ATOM 415 CD2 LEU A 109 -5.133 -2.003 7.365 1.00 13.98 C

ATOM 416 H LEU A 109 -5.088 -6.080 4.519 1.00 0.00 H

ATOM 417 HA LEU A 109 -2.695 -4.781 5.321 1.00 0.00 H

ATOM 418 HB2 LEU A 109 -5.011 -3.746 5.174 1.00 0.00 H

ATOM 419 HB3 LEU A 109 -5.436 -4.600 6.630 1.00 0.00 H

ATOM 420 HG LEU A 109 -3.589 -3.380 7.837 1.00 0.00 H

ATOM 421 HD21 LEU A 109 -5.105 -1.844 8.445 1.00 0.00 H

ATOM 422 HD22 LEU A 109 -5.059 -1.022 6.897 1.00 0.00 H

ATOM 423 H01 LEU A 109 -6.063 -2.479 7.056 1.00 13.98 H

ATOM 424 H02 LEU A 109 -2.678 -1.288 6.666 1.00 14.26 H

ATOM 425 H03 LEU A 109 -3.205 -2.040 5.141 1.00 14.26 H

ATOM 426 H04 LEU A 109 -1.993 -2.850 6.161 1.00 14.26 H

ATOM 427 N ALA A 110 -3.880 -7.014 7.475 1.00 10.47 N

ATOM 428 CA ALA A 110 -3.524 -7.890 8.597 1.00 11.01 C

ATOM 429 C ALA A 110 -2.194 -8.631 8.386 1.00 11.00 C

ATOM 430 O ALA A 110 -1.340 -8.605 9.270 1.00 11.36 O

ATOM 431 CB ALA A 110 -4.648 -8.897 8.872 1.00 10.99 C

ATOM 432 H ALA A 110 -4.787 -7.182 7.049 1.00 0.00 H

ATOM 433 HA ALA A 110 -3.420 -7.260 9.482 1.00 0.00 H

ATOM 434 HB1 ALA A 110 -4.374 -9.576 9.679 1.00 0.00 H

ATOM 435 HB2 ALA A 110 -5.562 -8.390 9.173 1.00 0.00 H

ATOM 436 HB3 ALA A 110 -4.876 -9.506 7.997 1.00 0.00 H

ATOM 437 N LYS A 111 -2.022 -9.239 7.203 1.00 11.10 N

ATOM 438 CA LYS A 111 -0.790 -9.920 6.810 1.00 11.29 C

ATOM 439 H LYS A 111 -2.787 -9.238 6.532 1.00 0.00 H

ATOM 440 H01 LYS A 111 -0.511 -9.615 5.801 1.00 11.29 H

ATOM 441 H02 LYS A 111 -0.947 -10.998 6.834 1.00 11.29 H

ATOM 442 H03 LYS A 111 0.008 -9.654 7.503 1.00 11.29 H

TER

END

**BTK Protein**

ATOM 1 CA GLU A 407 -49.405 18.829 16.659 1.00-12.13 C

ATOM 2 C GLU A 407 -47.891 19.063 16.485 1.00-12.13 C

ATOM 3 O GLU A 407 -47.193 19.239 17.486 1.00-12.13 O

ATOM 4 H01 GLU A 407 -49.941 19.289 15.829 1.00-12.13 H

ATOM 5 H02 GLU A 407 -49.608 17.758 16.673 1.00-12.13 H

ATOM 6 H03 GLU A 407 -49.737 19.275 17.597 1.00-12.13 H

ATOM 7 N LEU A 408 -47.417 19.065 15.233 1.00-12.13 N

ATOM 8 CA LEU A 408 -46.011 19.291 14.888 1.00-12.13 C

ATOM 9 C LEU A 408 -45.764 20.746 14.452 1.00-12.13 C

ATOM 10 O LEU A 408 -44.647 21.236 14.617 1.00-12.13 O

ATOM 11 CB LEU A 408 -45.568 18.299 13.788 1.00-12.13 C

ATOM 12 CG LEU A 408 -45.668 16.812 14.187 1.00-12.13 C

ATOM 13 CD1 LEU A 408 -45.256 15.891 13.020 1.00-12.13 C

ATOM 14 CD2 LEU A 408 -44.879 16.504 15.473 1.00-12.13 C

ATOM 15 H LEU A 408 -48.051 18.963 14.442 1.00-12.13 H

ATOM 16 HA LEU A 408 -45.381 19.130 15.764 1.00-12.13 H

ATOM 17 HB2 LEU A 408 -46.149 18.476 12.881 1.00-12.13 H

ATOM 18 HB3 LEU A 408 -44.531 18.509 13.526 1.00-12.13 H

ATOM 19 HG LEU A 408 -46.715 16.602 14.397 1.00-12.13 H

ATOM 20 HD11 LEU A 408 -46.032 15.158 12.799 1.00-12.13 H

ATOM 21 HD12 LEU A 408 -45.075 16.451 12.102 1.00-12.13 H

ATOM 22 HD13 LEU A 408 -44.341 15.338 13.229 1.00-12.13 H

ATOM 23 HD21 LEU A 408 -44.528 15.476 15.511 1.00-12.13 H

ATOM 24 HD22 LEU A 408 -44.006 17.149 15.580 1.00-12.13 H

ATOM 25 HD23 LEU A 408 -45.512 16.654 16.347 1.00-12.13 H

ATOM 26 N GLY A 409 -46.797 21.427 13.945 1.00-12.13 N

ATOM 27 CA GLY A 409 -46.741 22.835 13.566 1.00-12.13 C

ATOM 28 C GLY A 409 -47.649 23.051 12.353 1.00 4.09 C

ATOM 29 O GLY A 409 -48.437 22.177 11.987 1.00 4.09 O

ATOM 30 H GLY A 409 -47.667 20.950 13.742 1.00-12.13 H

ATOM 31 HA2 GLY A 409 -47.090 23.448 14.398 1.00-12.13 H

ATOM 32 HA3 GLY A 409 -45.726 23.152 13.321 1.00-12.13 H

ATOM 33 N THR A 410 -47.539 24.234 11.743 1.00 4.09 N

ATOM 34 CA THR A 410 -48.267 24.640 10.544 1.00 4.09 C

ATOM 35 C THR A 410 -47.258 24.961 9.426 1.00 -8.27 C

ATOM 36 O THR A 410 -46.266 25.647 9.684 1.00 -8.27 O

ATOM 37 CB THR A 410 -49.117 25.907 10.842 1.00 4.09 C

ATOM 38 CG2 THR A 410 -49.948 26.446 9.666 1.00 4.09 C

ATOM 39 OG1 THR A 410 -50.024 25.616 11.894 1.00 4.09 O

ATOM 40 H THR A 410 -46.833 24.891 12.047 1.00 4.09 H

ATOM 41 HA THR A 410 -48.916 23.842 10.189 1.00 4.09 H

ATOM 42 HB THR A 410 -48.459 26.708 11.187 1.00 4.09 H

ATOM 43 HG1 THR A 410 -49.505 25.291 12.643 1.00 4.09 H

ATOM 44 HG21 THR A 410 -50.557 27.297 9.972 1.00 4.09 H

ATOM 45 HG22 THR A 410 -49.318 26.788 8.845 1.00 4.09 H

ATOM 46 HG23 THR A 410 -50.620 25.684 9.275 1.00 4.09 H

ATOM 47 N GLY A 411 -47.521 24.447 8.221 1.00 -8.27 N

ATOM 48 CA GLY A 411 -46.742 24.702 7.013 1.00 -8.27 C

ATOM 49 C GLY A 411 -47.637 25.408 5.988 1.00 -2.24 C

ATOM 50 O GLY A 411 -48.721 25.899 6.314 1.00 -2.24 O

ATOM 51 H GLY A 411 -48.378 23.916 8.085 1.00 -8.27 H

ATOM 52 HA2 GLY A 411 -45.861 25.316 7.205 1.00 -8.27 H

ATOM 53 HA3 GLY A 411 -46.396 23.754 6.609 1.00 -8.27 H

ATOM 54 N GLN A 412 -47.172 25.461 4.732 1.00 -2.24 N

ATOM 55 CA GLN A 412 -47.837 26.127 3.605 1.00 -2.24 C

ATOM 56 C GLN A 412 -49.223 25.529 3.271 1.00 -7.46 C

ATOM 57 O GLN A 412 -50.092 26.262 2.797 1.00 -7.46 O

ATOM 58 CB GLN A 412 -46.879 26.094 2.387 1.00 -2.24 C

ATOM 59 CG GLN A 412 -47.322 26.853 1.116 1.00 -2.24 C

ATOM 60 CD GLN A 412 -47.416 28.372 1.289 1.00 -2.24 C

ATOM 61 NE2 GLN A 412 -48.384 28.996 0.632 1.00 -2.24 N

ATOM 62 OE1 GLN A 412 -46.605 28.988 1.976 1.00 -2.24 O

ATOM 63 H GLN A 412 -46.295 25.008 4.519 1.00 -2.24 H

ATOM 64 HA GLN A 412 -47.993 27.168 3.897 1.00 -2.24 H

ATOM 65 HB2 GLN A 412 -45.899 26.469 2.686 1.00 -2.24 H

ATOM 66 HB3 GLN A 412 -46.720 25.056 2.103 1.00 -2.24 H

ATOM 67 HG2 GLN A 412 -46.596 26.664 0.324 1.00 -2.24 H

ATOM 68 HG3 GLN A 412 -48.268 26.455 0.746 1.00 -2.24 H

ATOM 69 HE21 GLN A 412 -49.051 28.467 0.094 1.00 -2.24 H

ATOM 70 HE22 GLN A 412 -48.453 30.018 0.597 1.00 -2.24 H

ATOM 71 N PHE A 413 -49.421 24.233 3.551 1.00 -7.46 N

ATOM 72 CA PHE A 413 -50.679 23.523 3.314 1.00 -7.46 C

ATOM 73 C PHE A 413 -51.559 23.429 4.575 1.00 0.21 C

ATOM 74 O PHE A 413 -52.613 22.798 4.521 1.00 0.21 O

ATOM 75 CB PHE A 413 -50.393 22.125 2.726 1.00 -7.46 C

ATOM 76 CG PHE A 413 -49.727 22.093 1.357 1.00 -7.46 C

ATOM 77 CD1 PHE A 413 -48.670 21.191 1.111 1.00 -7.46 C

ATOM 78 CD2 PHE A 413 -50.241 22.851 0.282 1.00 -7.46 C

ATOM 79 CE1 PHE A 413 -48.110 21.103 -0.156 1.00 -7.46 C

ATOM 80 CE2 PHE A 413 -49.672 22.742 -0.980 1.00 -7.46 C

ATOM 81 CZ PHE A 413 -48.603 21.881 -1.196 1.00 -7.46 C

ATOM 82 H PHE A 413 -48.696 23.692 4.010 1.00 -7.46 H

ATOM 83 HA PHE A 413 -51.278 24.072 2.589 1.00 -7.46 H

ATOM 84 HB2 PHE A 413 -49.779 21.562 3.430 1.00 -7.46 H

ATOM 85 HB3 PHE A 413 -51.325 21.568 2.638 1.00 -7.46 H

ATOM 86 HD1 PHE A 413 -48.289 20.567 1.905 1.00 -7.46 H

ATOM 87 HD2 PHE A 413 -51.087 23.509 0.424 1.00 -7.46 H

ATOM 88 HE1 PHE A 413 -47.294 20.418 -0.331 1.00 -7.46 H

ATOM 89 HE2 PHE A 413 -50.069 23.323 -1.799 1.00 -7.46 H

ATOM 90 HZ PHE A 413 -48.165 21.803 -2.181 1.00 -7.46 H

ATOM 91 N GLY A 414 -51.155 24.060 5.684 1.00 0.21 N

ATOM 92 CA GLY A 414 -51.892 24.027 6.944 1.00 0.21 C

ATOM 93 C GLY A 414 -51.163 23.127 7.948 1.00 1.00 C

ATOM 94 O GLY A 414 -49.946 22.929 7.860 1.00 1.00 O

ATOM 95 H GLY A 414 -50.261 24.538 5.686 1.00 0.21 H

ATOM 96 HA2 GLY A 414 -51.938 25.040 7.343 1.00 0.21 H

ATOM 97 HA3 GLY A 414 -52.923 23.691 6.818 1.00 0.21 H

ATOM 98 N VAL A 415 -51.908 22.667 8.959 1.00 1.00 N

ATOM 99 CA VAL A 415 -51.402 21.944 10.124 1.00 1.00 C

ATOM 100 C VAL A 415 -50.863 20.534 9.785 1.00 -6.01 C

ATOM 101 O VAL A 415 -51.438 19.822 8.956 1.00 -6.01 O

ATOM 102 H VAL A 415 -52.905 22.817 8.945 1.00 1.00 H

ATOM 103 H01 VAL A 415 -50.595 22.526 10.569 1.00 1.00 H

ATOM 104 H04 VAL A 415 -52.239 21.812 10.809 1.00 1.00 H

ATOM 105 N VAL A 416 -49.769 20.164 10.456 1.00 -6.01 N

ATOM 106 CA VAL A 416 -49.186 18.833 10.437 1.00 -6.01 C

ATOM 107 C VAL A 416 -49.203 18.312 11.883 1.00 -6.01 C

ATOM 108 O VAL A 416 -48.886 19.060 12.815 1.00 -6.01 O

ATOM 109 CB VAL A 416 -47.714 18.851 9.939 1.00 -6.01 C

ATOM 110 CG1 VAL A 416 -47.103 17.439 9.812 1.00 -6.01 C

ATOM 111 CG2 VAL A 416 -47.585 19.588 8.596 1.00 -6.01 C

ATOM 112 H VAL A 416 -49.348 20.803 11.129 1.00 -6.01 H

ATOM 113 HA VAL A 416 -49.773 18.167 9.806 1.00 -6.01 H

ATOM 114 HB VAL A 416 -47.105 19.399 10.661 1.00 -6.01 H

ATOM 115 HG11 VAL A 416 -46.085 17.487 9.429 1.00 -6.01 H

ATOM 116 HG12 VAL A 416 -47.056 16.921 10.769 1.00 -6.01 H

ATOM 117 HG13 VAL A 416 -47.682 16.818 9.128 1.00 -6.01 H

ATOM 118 HG21 VAL A 416 -46.580 19.486 8.189 1.00 -6.01 H

ATOM 119 HG22 VAL A 416 -48.288 19.196 7.863 1.00 -6.01 H

ATOM 120 HG23 VAL A 416 -47.779 20.656 8.703 1.00 -6.01 H

ATOM 121 N LYS A 417 -49.588 17.046 12.051 1.00 -6.01 N

ATOM 122 CA LYS A 417 -49.692 16.379 13.341 1.00 -6.01 C

ATOM 123 H LYS A 417 -49.820 16.474 11.240 1.00 -6.01 H

ATOM 124 H01 LYS A 417 -49.228 15.395 13.280 1.00 -6.01 H

ATOM 125 H02 LYS A 417 -50.743 16.269 13.609 1.00 -6.01 H

ATOM 126 H03 LYS A 417 -49.185 16.974 14.100 1.00 -6.01 H

ATOM 127 CA VAL A 427 -46.550 8.712 11.107 1.00 -1.05 C

ATOM 128 C VAL A 427 -46.720 10.202 11.433 1.00 -1.05 C

ATOM 129 O VAL A 427 -47.152 10.564 12.530 1.00 -1.05 O

ATOM 130 H01 VAL A 427 -47.342 8.141 11.591 1.00 -1.05 H

ATOM 131 H02 VAL A 427 -46.604 8.567 10.028 1.00 -1.05 H

ATOM 132 H04 VAL A 427 -45.582 8.369 11.471 1.00 -1.05 H

ATOM 133 N ALA A 428 -46.377 11.035 10.452 1.00 -1.05 N

ATOM 134 CA ALA A 428 -46.722 12.443 10.406 1.00 -1.05 C

ATOM 135 C ALA A 428 -47.848 12.581 9.375 1.00 -0.71 C

ATOM 136 O ALA A 428 -47.773 11.987 8.298 1.00 -0.71 O

ATOM 137 CB ALA A 428 -45.486 13.248 10.002 1.00 -1.05 C

ATOM 138 H ALA A 428 -46.109 10.636 9.552 1.00 -1.05 H

ATOM 139 HA ALA A 428 -47.064 12.797 11.379 1.00 -1.05 H

ATOM 140 HB1 ALA A 428 -45.733 14.300 9.851 1.00 -1.05 H

ATOM 141 HB2 ALA A 428 -44.715 13.199 10.769 1.00 -1.05 H

ATOM 142 HB3 ALA A 428 -45.062 12.863 9.075 1.00 -1.05 H

ATOM 143 N ILE A 429 -48.886 13.325 9.737 1.00 -0.71 N

ATOM 144 CA ILE A 429 -50.100 13.487 8.962 1.00 -0.71 C

ATOM 145 C ILE A 429 -50.238 14.993 8.682 1.00 -9.02 C

ATOM 146 O ILE A 429 -50.501 15.768 9.606 1.00 -9.02 O

ATOM 147 H ILE A 429 -48.889 13.789 10.648 1.00 -0.71 H

ATOM 148 H01 ILE A 429 -50.027 12.934 8.026 1.00 -0.71 H

ATOM 149 H03 ILE A 429 -50.967 13.104 9.501 1.00 -0.71 H

ATOM 150 N LYS A 430 -50.045 15.387 7.418 1.00 -9.02 N

ATOM 151 CA LYS A 430 -50.459 16.692 6.903 1.00 -9.02 C

ATOM 152 C LYS A 430 -51.992 16.685 6.784 1.00 -9.02 C

ATOM 153 O LYS A 430 -52.526 15.789 6.128 1.00 -9.02 O

ATOM 154 CB LYS A 430 -49.850 16.925 5.502 1.00 -9.02 C

ATOM 155 CG LYS A 430 -48.370 17.351 5.434 1.00 -9.02 C

ATOM 156 CD LYS A 430 -47.825 17.181 4.000 1.00 -9.02 C

ATOM 157 CE LYS A 430 -46.700 18.134 3.556 1.00 -9.02 C

ATOM 158 NZ LYS A 430 -45.518 18.118 4.426 1.00 -9.02 N1+

ATOM 159 H LYS A 430 -49.890 14.667 6.716 1.00 -9.02 H

ATOM 160 HA LYS A 430 -50.133 17.482 7.582 1.00 -9.02 H

ATOM 161 HB2 LYS A 430 -50.013 16.031 4.902 1.00 -9.02 H

ATOM 162 HB3 LYS A 430 -50.415 17.713 5.004 1.00 -9.02 H

ATOM 163 HG2 LYS A 430 -48.287 18.395 5.735 1.00 -9.02 H

ATOM 164 HG3 LYS A 430 -47.769 16.772 6.136 1.00 -9.02 H

ATOM 165 HD2 LYS A 430 -47.476 16.153 3.882 1.00 -9.02 H

ATOM 166 HD3 LYS A 430 -48.642 17.287 3.287 1.00 -9.02 H

ATOM 167 HE2 LYS A 430 -46.387 17.847 2.552 1.00 -9.02 H

ATOM 168 HE3 LYS A 430 -47.058 19.160 3.482 1.00 -9.02 H

ATOM 169 HZ1 LYS A 430 -44.712 18.431 3.861 1.00 -9.02 H

ATOM 170 HZ2 LYS A 430 -45.631 18.774 5.188 1.00 -9.02 H

ATOM 171 HZ3 LYS A 430 -45.319 17.181 4.778 1.00 -9.02 H

ATOM 172 N MET A 431 -52.664 17.669 7.388 1.00 -9.02 N

ATOM 173 CA MET A 431 -54.096 17.899 7.226 1.00 -9.02 C

ATOM 174 H MET A 431 -52.164 18.401 7.890 1.00 -9.02 H

ATOM 175 H01 MET A 431 -54.266 18.929 6.913 1.00 -9.02 H

ATOM 176 H02 MET A 431 -54.602 17.720 8.175 1.00 -9.02 H

ATOM 177 H03 MET A 431 -54.491 17.219 6.471 1.00 -9.02 H

ATOM 178 CA LEU A 457 -36.647 10.386 0.636 1.00 -0.04 C

ATOM 179 C LEU A 457 -37.506 10.060 1.866 1.00 -0.04 C

ATOM 180 O LEU A 457 -37.530 8.903 2.291 1.00 -0.04 O

ATOM 181 H01 LEU A 457 -37.131 9.997 -0.260 1.00 -0.04 H

ATOM 182 H02 LEU A 457 -36.533 11.466 0.548 1.00 -0.04 H

ATOM 183 H04 LEU A 457 -35.665 9.926 0.747 1.00 -0.04 H

ATOM 184 N VAL A 458 -38.219 11.058 2.399 1.00 -0.04 N

ATOM 185 CA VAL A 458 -39.257 10.858 3.415 1.00 -0.04 C

ATOM 186 C VAL A 458 -40.435 10.106 2.753 1.00 -0.04 C

ATOM 187 O VAL A 458 -41.017 10.626 1.796 1.00 -0.04 O

ATOM 188 CB VAL A 458 -39.758 12.225 3.976 1.00 -0.04 C

ATOM 189 CG1 VAL A 458 -40.935 12.117 4.969 1.00 -0.04 C

ATOM 190 CG2 VAL A 458 -38.618 13.010 4.648 1.00 -0.04 C

ATOM 191 H VAL A 458 -38.151 11.994 2.009 1.00 -0.04 H

ATOM 192 HA VAL A 458 -38.845 10.262 4.232 1.00 -0.04 H

ATOM 193 HB VAL A 458 -40.105 12.825 3.133 1.00 -0.04 H

ATOM 194 HG11 VAL A 458 -41.232 13.101 5.333 1.00 -0.04 H

ATOM 195 HG12 VAL A 458 -41.820 11.672 4.514 1.00 -0.04 H

ATOM 196 HG13 VAL A 458 -40.665 11.514 5.837 1.00 -0.04 H

ATOM 197 HG21 VAL A 458 -38.943 14.011 4.923 1.00 -0.04 H

ATOM 198 HG22 VAL A 458 -38.274 12.506 5.550 1.00 -0.04 H

ATOM 199 HG23 VAL A 458 -37.759 13.133 3.992 1.00 -0.04 H

ATOM 200 N GLN A 459 -40.714 8.874 3.200 1.00 -0.04 N

ATOM 201 CA GLN A 459 -41.683 7.997 2.544 1.00 -0.04 C

ATOM 202 H GLN A 459 -40.193 8.491 4.001 1.00 -0.04 H

ATOM 203 H01 GLN A 459 -41.237 7.570 1.646 1.00 -0.04 H

ATOM 204 H02 GLN A 459 -41.968 7.196 3.225 1.00 -0.04 H

ATOM 205 H03 GLN A 459 -42.567 8.573 2.271 1.00 -0.04 H

ATOM 206 CA ILE A 473 -49.722 10.259 5.779 1.00 -0.82 C

ATOM 207 C ILE A 473 -48.299 9.911 5.302 1.00 -0.82 C

ATOM 208 O ILE A 473 -48.134 9.176 4.323 1.00 -0.82 O

ATOM 209 H01 ILE A 473 -49.744 10.286 6.868 1.00 -0.82 H

ATOM 210 H02 ILE A 473 -50.010 11.234 5.385 1.00 -0.82 H

ATOM 211 H03 ILE A 473 -50.420 9.502 5.421 1.00 -0.82 H

ATOM 212 N THR A 474 -47.296 10.464 5.982 1.00 -0.82 N

ATOM 213 CA THR A 474 -45.888 10.302 5.659 1.00 -0.82 C

ATOM 214 C THR A 474 -45.135 9.740 6.881 1.00-19.72 C

ATOM 215 O THR A 474 -45.637 9.763 8.008 1.00-19.72 O

ATOM 216 CB THR A 474 -45.265 11.674 5.244 1.00 -0.82 C

ATOM 217 CG2 THR A 474 -45.985 12.335 4.059 1.00 -0.82 C

ATOM 218 OG1 THR A 474 -45.210 12.631 6.293 1.00 -0.82 O

ATOM 219 H THR A 474 -47.474 11.051 6.798 1.00 -0.82 H

ATOM 220 HA THR A 474 -45.763 9.592 4.841 1.00 -0.82 H

ATOM 221 HB THR A 474 -44.235 11.490 4.935 1.00 -0.82 H

ATOM 222 HG1 THR A 474 -44.646 12.270 6.989 1.00 -0.82 H

ATOM 223 HG21 THR A 474 -45.454 13.226 3.723 1.00 -0.82 H

ATOM 224 HG22 THR A 474 -46.050 11.654 3.210 1.00 -0.82 H

ATOM 225 HG23 THR A 474 -46.999 12.636 4.322 1.00 -0.82 H

ATOM 226 N GLU A 475 -43.916 9.258 6.626 1.00-19.72 N

ATOM 227 CA GLU A 475 -42.902 8.871 7.603 1.00-19.72 C

ATOM 228 C GLU A 475 -42.638 9.988 8.637 1.00-10.52 C

ATOM 229 O GLU A 475 -42.476 11.144 8.245 1.00-10.52 O

ATOM 230 CB GLU A 475 -41.655 8.476 6.785 1.00-19.72 C

ATOM 231 CG GLU A 475 -40.388 8.092 7.573 1.00-19.72 C

ATOM 232 CD GLU A 475 -39.233 7.683 6.660 1.00-19.72 C

ATOM 233 OE1 GLU A 475 -38.238 7.142 7.186 1.00-19.72 O

ATOM 234 OE2 GLU A 475 -39.315 7.885 5.432 1.00-19.72 O1-

ATOM 235 H GLU A 475 -43.619 9.202 5.654 1.00-19.72 H

ATOM 236 HA GLU A 475 -43.264 7.995 8.136 1.00-19.72 H

ATOM 237 HB2 GLU A 475 -41.923 7.635 6.145 1.00-19.72 H

ATOM 238 HB3 GLU A 475 -41.405 9.295 6.109 1.00-19.72 H

ATOM 239 HG2 GLU A 475 -40.050 8.929 8.186 1.00-19.72 H

ATOM 240 HG3 GLU A 475 -40.609 7.269 8.253 1.00-19.72 H

ATOM 241 N TYR A 476 -42.634 9.630 9.930 1.00-10.52 N

ATOM 242 CA TYR A 476 -42.320 10.552 11.022 1.00-10.52 C

ATOM 243 C TYR A 476 -40.798 10.728 11.139 1.00 -7.90 C

ATOM 244 O TYR A 476 -40.069 9.736 11.207 1.00 -7.90 O

ATOM 245 CB TYR A 476 -42.937 10.043 12.343 1.00-10.52 C

ATOM 246 CG TYR A 476 -42.658 10.892 13.578 1.00-10.52 C

ATOM 247 CD1 TYR A 476 -43.012 12.259 13.598 1.00-10.52 C

ATOM 248 CD2 TYR A 476 -42.050 10.320 14.716 1.00-10.52 C

ATOM 249 CE1 TYR A 476 -42.772 13.037 14.747 1.00-10.52 C

ATOM 250 CE2 TYR A 476 -41.822 11.097 15.868 1.00-10.52 C

ATOM 251 CZ TYR A 476 -42.194 12.455 15.889 1.00-10.52 C

ATOM 252 OH TYR A 476 -41.991 13.209 17.011 1.00-10.52 O

ATOM 253 H TYR A 476 -42.750 8.660 10.183 1.00-10.52 H

ATOM 254 HA TYR A 476 -42.776 11.517 10.791 1.00-10.52 H

ATOM 255 HB2 TYR A 476 -44.018 9.971 12.238 1.00-10.52 H

ATOM 256 HB3 TYR A 476 -42.587 9.027 12.532 1.00-10.52 H

ATOM 257 HD1 TYR A 476 -43.471 12.722 12.737 1.00-10.52 H

ATOM 258 HD2 TYR A 476 -41.764 9.277 14.719 1.00-10.52 H

ATOM 259 HE1 TYR A 476 -43.030 14.084 14.743 1.00-10.52 H

ATOM 260 HE2 TYR A 476 -41.370 10.634 16.734 1.00-10.52 H

ATOM 261 HH TYR A 476 -41.310 12.816 17.575 1.00-10.52 H

ATOM 262 N MET A 477 -40.360 11.985 11.173 1.00 -7.90 N

ATOM 263 CA MET A 477 -38.971 12.405 11.223 1.00 -7.90 C

ATOM 264 C MET A 477 -38.762 13.102 12.571 1.00-11.01 C

ATOM 265 O MET A 477 -39.019 14.299 12.698 1.00-11.01 O

ATOM 266 CB MET A 477 -38.705 13.331 10.025 1.00 -7.90 C

ATOM 267 CG MET A 477 -38.925 12.654 8.657 1.00 -7.90 C

ATOM 268 SD MET A 477 -37.823 11.258 8.298 1.00 -7.90 S

ATOM 269 CE MET A 477 -36.275 12.166 8.095 1.00 -7.90 C

ATOM 270 H MET A 477 -41.033 12.753 11.127 1.00 -7.90 H

ATOM 271 HA MET A 477 -38.294 11.550 11.162 1.00 -7.90 H

ATOM 272 HB2 MET A 477 -39.342 14.215 10.081 1.00 -7.90 H

ATOM 273 HB3 MET A 477 -37.686 13.707 10.103 1.00 -7.90 H

ATOM 274 HG2 MET A 477 -39.954 12.303 8.571 1.00 -7.90 H

ATOM 275 HG3 MET A 477 -38.809 13.385 7.860 1.00 -7.90 H

ATOM 276 HE1 MET A 477 -35.448 11.486 7.910 1.00 -7.90 H

ATOM 277 HE2 MET A 477 -36.349 12.880 7.277 1.00 -7.90 H

ATOM 278 HE3 MET A 477 -36.048 12.724 8.993 1.00 -7.90 H

ATOM 279 N ALA A 478 -38.372 12.304 13.574 1.00-11.01 N

ATOM 280 CA ALA A 478 -38.390 12.642 14.998 1.00-11.01 C

ATOM 281 C ALA A 478 -37.587 13.885 15.428 1.00-21.62 C

ATOM 282 O ALA A 478 -37.971 14.518 16.412 1.00-21.62 O

ATOM 283 CB ALA A 478 -37.931 11.413 15.797 1.00-11.01 C

ATOM 284 H ALA A 478 -38.232 11.327 13.366 1.00-11.01 H

ATOM 285 HA ALA A 478 -39.431 12.843 15.255 1.00-11.01 H

ATOM 286 HB1 ALA A 478 -37.966 11.607 16.870 1.00-11.01 H

ATOM 287 HB2 ALA A 478 -38.571 10.552 15.604 1.00-11.01 H

ATOM 288 HB3 ALA A 478 -36.906 11.136 15.547 1.00-11.01 H

ATOM 289 N ASN A 479 -36.503 14.217 14.715 1.00-21.62 N

ATOM 290 CA ASN A 479 -35.654 15.380 15.020 1.00-21.62 C

ATOM 291 C ASN A 479 -36.061 16.621 14.211 1.00-14.21 C

ATOM 292 O ASN A 479 -35.529 17.698 14.478 1.00-14.21 O

ATOM 293 CB ASN A 479 -34.161 15.012 14.838 1.00-21.62 C

ATOM 294 CG ASN A 479 -33.578 14.287 16.053 1.00-21.62 C

ATOM 295 ND2 ASN A 479 -32.961 13.129 15.856 1.00-21.62 N

ATOM 296 OD1 ASN A 479 -33.667 14.776 17.177 1.00-21.62 O

ATOM 297 H ASN A 479 -36.283 13.713 13.864 1.00-21.62 H

ATOM 298 HA ASN A 479 -35.809 15.617 16.074 1.00-21.62 H

ATOM 299 HB2 ASN A 479 -33.998 14.452 13.919 1.00-21.62 H

ATOM 300 HB3 ASN A 479 -33.567 15.922 14.738 1.00-21.62 H

ATOM 301 HD21 ASN A 479 -32.933 12.703 14.928 1.00-21.62 H

ATOM 302 HD22 ASN A 479 -32.532 12.636 16.625 1.00-21.62 H

ATOM 303 N GLY A 480 -37.044 16.498 13.311 1.00-14.21 N

ATOM 304 CA GLY A 480 -37.634 17.620 12.590 1.00-14.21 C

ATOM 305 C GLY A 480 -36.662 18.208 11.556 1.00 -0.31 C

ATOM 306 O GLY A 480 -35.807 17.508 11.016 1.00 -0.31 O

ATOM 307 H GLY A 480 -37.447 15.584 13.138 1.00-14.21 H

ATOM 308 HA2 GLY A 480 -38.526 17.268 12.071 1.00-14.21 H

ATOM 309 HA3 GLY A 480 -37.956 18.386 13.297 1.00-14.21 H

ATOM 310 N CYS A 481 -36.883 19.480 11.219 1.00 -0.31 N

ATOM 311 CA CYS A 481 -36.254 20.236 10.135 1.00 -0.31 C

ATOM 312 C CYS A 481 -34.714 20.337 10.256 1.00 -0.31 C

ATOM 313 O CYS A 481 -34.212 20.696 11.325 1.00 -0.31 O

ATOM 314 CB CYS A 481 -36.948 21.615 10.055 1.00 -0.31 C

ATOM 315 SG CYS A 481 -36.222 22.774 8.867 1.00 -0.31 S

ATOM 316 H CYS A 481 -37.597 19.982 11.724 1.00 -0.31 H

ATOM 317 HA CYS A 481 -36.472 19.702 9.210 1.00 -0.31 H

ATOM 318 HB2 CYS A 481 -37.998 21.484 9.791 1.00 -0.31 H

ATOM 319 HB3 CYS A 481 -36.932 22.093 11.034 1.00 -0.31 H

ATOM 320 HG CYS A 481 -36.609 22.105 7.773 1.00 -0.31 H

ATOM 321 N LEU A 482 -34.001 20.082 9.145 1.00 -0.31 N

ATOM 322 CA LEU A 482 -32.541 20.178 9.037 1.00 -0.31 C

ATOM 323 H LEU A 482 -34.493 19.816 8.291 1.00 -0.31 H

ATOM 324 H01 LEU A 482 -32.229 19.877 8.037 1.00 -0.31 H

ATOM 325 H02 LEU A 482 -32.229 21.206 9.221 1.00 -0.31 H

ATOM 326 H03 LEU A 482 -32.079 19.522 9.774 1.00 -0.31 H

ATOM 327 CA LEU A 483 -32.311 24.041 9.083 1.00 1.89 C

ATOM 328 C LEU A 483 -32.272 24.386 10.585 1.00 1.89 C

ATOM 329 O LEU A 483 -31.325 25.022 11.044 1.00 1.89 O

ATOM 330 H01 LEU A 483 -31.451 23.421 8.830 1.00 1.89 H

ATOM 331 H02 LEU A 483 -33.230 23.499 8.858 1.00 1.89 H

ATOM 332 H03 LEU A 483 -32.279 24.961 8.499 1.00 1.89 H

ATOM 333 N ASN A 484 -33.276 23.910 11.333 1.00 1.89 N

ATOM 334 CA ASN A 484 -33.342 24.046 12.792 1.00 1.89 C

ATOM 335 C ASN A 484 -32.233 23.225 13.468 1.00 -0.54 C

ATOM 336 O ASN A 484 -31.525 23.776 14.308 1.00 -0.54 O

ATOM 337 CB ASN A 484 -34.748 23.654 13.301 1.00 1.89 C

ATOM 338 CG ASN A 484 -35.773 24.778 13.141 1.00 1.89 C

ATOM 339 ND2 ASN A 484 -36.496 24.827 12.027 1.00 1.89 N

ATOM 340 OD1 ASN A 484 -35.924 25.612 14.031 1.00 1.89 O

ATOM 341 HA ASN A 484 -33.183 25.111 12.984 1.00 1.89 H

ATOM 342 HB2 ASN A 484 -35.101 22.727 12.850 1.00 1.89 H

ATOM 343 HB3 ASN A 484 -34.692 23.452 14.373 1.00 1.89 H

ATOM 344 HD21 ASN A 484 -36.367 24.171 11.273 1.00 1.89 H

ATOM 345 HD22 ASN A 484 -37.181 25.558 11.903 1.00 1.89 H

ATOM 346 H01 ASN A 484 -34.031 23.430 10.865 1.00 1.89 H

ATOM 347 N TYR A 485 -32.065 21.964 13.047 1.00 -0.54 N

ATOM 348 CA TYR A 485 -31.052 21.023 13.543 1.00 -0.54 C

ATOM 349 C TYR A 485 -29.605 21.555 13.401 1.00 -0.54 C

ATOM 350 O TYR A 485 -28.798 21.390 14.318 1.00 -0.54 O

ATOM 351 CB TYR A 485 -31.266 19.670 12.825 1.00 -0.54 C

ATOM 352 CG TYR A 485 -30.534 18.461 13.381 1.00 -0.54 C

ATOM 353 CD1 TYR A 485 -31.059 17.770 14.492 1.00 -0.54 C

ATOM 354 CD2 TYR A 485 -29.363 17.987 12.754 1.00 -0.54 C

ATOM 355 CE1 TYR A 485 -30.417 16.612 14.975 1.00 -0.54 C

ATOM 356 CE2 TYR A 485 -28.725 16.826 13.234 1.00 -0.54 C

ATOM 357 CZ TYR A 485 -29.249 16.139 14.347 1.00 -0.54 C

ATOM 358 OH TYR A 485 -28.638 15.010 14.809 1.00 -0.54 O

ATOM 359 H TYR A 485 -32.694 21.602 12.338 1.00 -0.54 H

ATOM 360 HA TYR A 485 -31.241 20.877 14.607 1.00 -0.54 H

ATOM 361 HB2 TYR A 485 -32.327 19.417 12.846 1.00 -0.54 H

ATOM 362 HB3 TYR A 485 -31.010 19.768 11.772 1.00 -0.54 H

ATOM 363 HD1 TYR A 485 -31.960 18.121 14.976 1.00 -0.54 H

ATOM 364 HD2 TYR A 485 -28.956 18.503 11.896 1.00 -0.54 H

ATOM 365 HE1 TYR A 485 -30.826 16.090 15.829 1.00 -0.54 H

ATOM 366 HE2 TYR A 485 -27.836 16.470 12.736 1.00 -0.54 H

ATOM 367 HH TYR A 485 -27.818 14.811 14.340 1.00 -0.54 H

ATOM 368 N LEU A 486 -29.321 22.237 12.281 1.00 -0.54 N

ATOM 369 CA LEU A 486 -28.063 22.931 11.999 1.00 -0.54 C

ATOM 370 H LEU A 486 -30.021 22.261 11.542 1.00 -0.54 H

ATOM 371 H01 LEU A 486 -28.192 23.582 11.134 1.00 -0.54 H

ATOM 372 H02 LEU A 486 -27.775 23.528 12.864 1.00 -0.54 H

ATOM 373 H03 LEU A 486 -27.284 22.198 11.788 1.00 -0.54 H

ATOM 374 CA LEU A 518 -37.715 17.726 -10.101 1.00 0.42 C

ATOM 375 C LEU A 518 -38.299 18.015 -8.710 1.00 0.42 C

ATOM 376 O LEU A 518 -39.489 17.770 -8.489 1.00 0.42 O

ATOM 377 H01 LEU A 518 -38.326 16.977 -10.604 1.00 0.42 H

ATOM 378 H02 LEU A 518 -37.706 18.643 -10.690 1.00 0.42 H

ATOM 379 H03 LEU A 518 -36.696 17.352 -9.996 1.00 0.42 H

ATOM 380 N HIS A 519 -37.482 18.587 -7.820 1.00 0.42 N

ATOM 381 CA HIS A 519 -37.941 19.120 -6.538 1.00 0.42 C

ATOM 382 C HIS A 519 -38.502 20.540 -6.716 1.00 -0.52 C

ATOM 383 O HIS A 519 -39.641 20.782 -6.317 1.00 -0.52 O

ATOM 384 CB HIS A 519 -36.820 19.085 -5.479 1.00 0.42 C

ATOM 385 CG HIS A 519 -37.327 19.204 -4.063 1.00 0.42 C

ATOM 386 CD2 HIS A 519 -37.155 18.332 -3.014 1.00 0.42 C

ATOM 387 ND1 HIS A 519 -38.137 20.257 -3.627 1.00 0.42 N

ATOM 388 CE1 HIS A 519 -38.429 19.983 -2.363 1.00 0.42 C

ATOM 389 NE2 HIS A 519 -37.880 18.845 -1.948 1.00 0.42 N

ATOM 390 H HIS A 519 -36.528 18.840 -8.126 1.00 0.42 H

ATOM 391 HA HIS A 519 -38.743 18.475 -6.172 1.00 0.42 H

ATOM 392 HB2 HIS A 519 -36.278 18.141 -5.550 1.00 0.42 H

ATOM 393 HB3 HIS A 519 -36.083 19.869 -5.662 1.00 0.42 H

ATOM 394 HD2 HIS A 519 -36.611 17.402 -2.964 1.00 0.42 H

ATOM 395 HE1 HIS A 519 -39.062 20.603 -1.742 1.00 0.42 H

ATOM 396 HE2 HIS A 519 -38.047 18.422 -1.034 1.00 0.42 H

ATOM 397 N ARG A 520 -37.695 21.439 -7.299 1.00 -0.52 N

ATOM 398 CA ARG A 520 -37.942 22.869 -7.513 1.00 -0.52 C

ATOM 399 C ARG A 520 -37.834 23.759 -6.250 1.00-28.54 C

ATOM 400 O ARG A 520 -37.796 24.979 -6.416 1.00-28.54 O

ATOM 401 H ARG A 520 -36.758 21.146 -7.601 1.00 -0.52 H

ATOM 402 H01 ARG A 520 -37.217 23.230 -8.243 1.00 -0.52 H

ATOM 403 H03 ARG A 520 -38.976 22.948 -7.847 1.00 -0.52 H

ATOM 404 N ASP A 521 -37.744 23.197 -5.035 1.00-28.54 N

ATOM 405 CA ASP A 521 -37.591 23.956 -3.781 1.00-28.54 C

ATOM 406 C ASP A 521 -36.721 23.143 -2.802 1.00-28.54 C

ATOM 407 O ASP A 521 -37.079 22.919 -1.644 1.00-28.54 O

ATOM 408 CB ASP A 521 -38.951 24.412 -3.192 1.00-28.54 C

ATOM 409 CG ASP A 521 -38.866 25.412 -2.031 1.00-28.54 C

ATOM 410 OD1 ASP A 521 -37.809 26.042 -1.816 1.00-28.54 O

ATOM 411 OD2 ASP A 521 -39.919 25.620 -1.392 1.00-28.54 O1-

ATOM 412 H ASP A 521 -37.799 22.189 -4.933 1.00-28.54 H

ATOM 413 HA ASP A 521 -37.012 24.857 -3.996 1.00-28.54 H

ATOM 414 HB2 ASP A 521 -39.544 24.870 -3.983 1.00-28.54 H

ATOM 415 HB3 ASP A 521 -39.488 23.527 -2.844 1.00-28.54 H

ATOM 416 N LEU A 522 -35.584 22.658 -3.317 1.00-28.54 N

ATOM 417 CA LEU A 522 -34.583 21.930 -2.546 1.00-28.54 C

ATOM 418 H LEU A 522 -35.344 22.883 -4.270 1.00-28.54 H

ATOM 419 H01 LEU A 522 -35.004 21.642 -1.583 1.00-28.54 H

ATOM 420 H02 LEU A 522 -33.714 22.568 -2.386 1.00-28.54 H

ATOM 421 H03 LEU A 522 -34.281 21.037 -3.093 1.00-28.54 H

ATOM 422 CA ALA A 524 -32.640 22.963 4.344 1.00 21.32 C

ATOM 423 C ALA A 524 -33.977 22.963 5.108 1.00 21.32 C

ATOM 424 O ALA A 524 -34.166 22.135 5.998 1.00 21.32 O

ATOM 425 H01 ALA A 524 -32.733 22.349 3.448 1.00 21.32 H

ATOM 426 H02 ALA A 524 -32.383 23.984 4.061 1.00 21.32 H

ATOM 427 H04 ALA A 524 -31.857 22.556 4.983 1.00 21.32 H

ATOM 428 N ARG A 525 -34.894 23.864 4.730 1.00 21.32 N

ATOM 429 CA ARG A 525 -36.269 23.911 5.227 1.00 21.32 C

ATOM 430 C ARG A 525 -37.115 22.695 4.789 1.00 -6.73 C

ATOM 431 O ARG A 525 -38.065 22.363 5.493 1.00 -6.73 O

ATOM 432 CB ARG A 525 -36.940 25.241 4.816 1.00 21.32 C

ATOM 433 CG ARG A 525 -37.004 25.476 3.298 1.00 21.32 C

ATOM 434 CD ARG A 525 -37.851 26.681 2.878 1.00 21.32 C

ATOM 435 NE ARG A 525 -37.629 26.952 1.453 1.00 21.32 N

ATOM 436 CZ ARG A 525 -36.581 27.604 0.944 1.00 21.32 C

ATOM 437 NH1 ARG A 525 -35.733 28.254 1.740 1.00 21.32 N1+

ATOM 438 NH2 ARG A 525 -36.380 27.586 -0.365 1.00 21.32 N

ATOM 439 H ARG A 525 -34.641 24.506 3.992 1.00 21.32 H

ATOM 440 HA ARG A 525 -36.215 23.902 6.314 1.00 21.32 H

ATOM 441 HB2 ARG A 525 -37.951 25.267 5.227 1.00 21.32 H

ATOM 442 HB3 ARG A 525 -36.408 26.071 5.282 1.00 21.32 H

ATOM 443 HG2 ARG A 525 -36.014 25.514 2.848 1.00 21.32 H

ATOM 444 HG3 ARG A 525 -37.499 24.608 2.864 1.00 21.32 H

ATOM 445 HD2 ARG A 525 -38.896 26.373 2.915 1.00 21.32 H

ATOM 446 HD3 ARG A 525 -37.763 27.548 3.534 1.00 21.32 H

ATOM 447 HE ARG A 525 -38.238 26.472 0.790 1.00 21.32 H

ATOM 448 HH11 ARG A 525 -35.876 28.298 2.734 1.00 21.32 H

ATOM 449 HH12 ARG A 525 -34.911 28.720 1.376 1.00 21.32 H

ATOM 450 H01 ARG A 525 -37.020 27.088 -0.967 1.00 21.32 H

ATOM 451 H02 ARG A 525 -35.587 28.070 -0.761 1.00 21.32 H

ATOM 452 N ASN A 526 -36.772 22.069 3.654 1.00 -6.73 N

ATOM 453 CA ASN A 526 -37.492 20.940 3.045 1.00 -6.73 C

ATOM 454 C ASN A 526 -36.631 19.670 3.138 1.00 2.47 C

ATOM 455 O ASN A 526 -36.730 18.775 2.297 1.00 2.47 O

ATOM 456 CB ASN A 526 -37.892 21.276 1.584 1.00 -6.73 C

ATOM 457 CG ASN A 526 -39.015 22.310 1.464 1.00 -6.73 C

ATOM 458 ND2 ASN A 526 -38.948 23.163 0.450 1.00 -6.73 N

ATOM 459 OD1 ASN A 526 -39.951 22.329 2.260 1.00 -6.73 O

ATOM 460 H ASN A 526 -35.909 22.329 3.184 1.00 -6.73 H

ATOM 461 HA ASN A 526 -38.404 20.748 3.612 1.00 -6.73 H

ATOM 462 HB2 ASN A 526 -37.015 21.577 1.009 1.00 -6.73 H

ATOM 463 HB3 ASN A 526 -38.280 20.381 1.097 1.00 -6.73 H

ATOM 464 HD21 ASN A 526 -38.194 23.109 -0.239 1.00 -6.73 H

ATOM 465 HD22 ASN A 526 -39.665 23.858 0.276 1.00 -6.73 H

ATOM 466 N CYS A 527 -35.814 19.594 4.188 1.00 2.47 N

ATOM 467 CA CYS A 527 -35.038 18.429 4.569 1.00 2.47 C

ATOM 468 C CYS A 527 -35.350 18.192 6.050 1.00 -2.35 C

ATOM 469 O CYS A 527 -35.333 19.141 6.838 1.00 -2.35 O

ATOM 470 CB CYS A 527 -33.535 18.659 4.351 1.00 2.47 C

ATOM 471 SG CYS A 527 -33.136 18.710 2.581 1.00 2.47 S

ATOM 472 H CYS A 527 -35.803 20.348 4.864 1.00 2.47 H

ATOM 473 HA CYS A 527 -35.351 17.550 4.002 1.00 2.47 H

ATOM 474 HB2 CYS A 527 -33.221 19.594 4.814 1.00 2.47 H

ATOM 475 HB3 CYS A 527 -32.959 17.856 4.809 1.00 2.47 H

ATOM 476 HG CYS A 527 -33.741 19.884 2.346 1.00 2.47 H

ATOM 477 N LEU A 528 -35.650 16.941 6.399 1.00 -2.35 N

ATOM 478 CA LEU A 528 -36.021 16.516 7.743 1.00 -2.35 C

ATOM 479 C LEU A 528 -34.985 15.495 8.235 1.00 -2.35 C

ATOM 480 O LEU A 528 -34.376 14.805 7.418 1.00 -2.35 O

ATOM 481 CB LEU A 528 -37.423 15.874 7.688 1.00 -2.35 C

ATOM 482 CG LEU A 528 -38.599 16.845 7.439 1.00 -2.35 C

ATOM 483 CD1 LEU A 528 -39.904 16.069 7.217 1.00 -2.35 C

ATOM 484 CD2 LEU A 528 -38.789 17.859 8.579 1.00 -2.35 C

ATOM 485 H LEU A 528 -35.552 16.187 5.719 1.00 -2.35 H

ATOM 486 HA LEU A 528 -36.028 17.359 8.433 1.00 -2.35 H

ATOM 487 HB2 LEU A 528 -37.420 15.093 6.926 1.00 -2.35 H

ATOM 488 HB3 LEU A 528 -37.608 15.372 8.634 1.00 -2.35 H

ATOM 489 HG LEU A 528 -38.397 17.400 6.521 1.00 -2.35 H

ATOM 490 HD11 LEU A 528 -40.638 16.683 6.695 1.00 -2.35 H

ATOM 491 HD12 LEU A 528 -39.758 15.171 6.622 1.00 -2.35 H

ATOM 492 HD13 LEU A 528 -40.339 15.761 8.166 1.00 -2.35 H

ATOM 493 HD21 LEU A 528 -39.839 17.989 8.847 1.00 -2.35 H

ATOM 494 HD22 LEU A 528 -38.268 17.547 9.478 1.00 -2.35 H

ATOM 495 HD23 LEU A 528 -38.416 18.840 8.286 1.00 -2.35 H

ATOM 496 N VAL A 529 -34.814 15.402 9.554 1.00 -2.35 N

ATOM 497 CA VAL A 529 -33.839 14.547 10.221 1.00 -2.35 C

ATOM 498 H VAL A 529 -35.318 16.038 10.174 1.00 -2.35 H

ATOM 499 H01 VAL A 529 -34.278 14.134 11.129 1.00 -2.35 H

ATOM 500 H02 VAL A 529 -33.551 13.735 9.554 1.00 -2.35 H

ATOM 501 H03 VAL A 529 -32.958 15.134 10.479 1.00 -2.35 H

ATOM 502 CA VAL A 537 -35.577 15.035 1.528 1.00 -0.56 C

ATOM 503 C VAL A 537 -37.117 14.936 1.629 1.00 -0.56 C

ATOM 504 O VAL A 537 -37.673 13.842 1.483 1.00 -0.56 O

ATOM 505 H01 VAL A 537 -35.258 14.738 0.529 1.00 -0.56 H

ATOM 506 H02 VAL A 537 -35.265 16.062 1.720 1.00 -0.56 H

ATOM 507 H03 VAL A 537 -35.122 14.374 2.266 1.00 -0.56 H

ATOM 508 N SER A 538 -37.762 16.075 1.898 1.00 -0.56 N

ATOM 509 CA SER A 538 -39.191 16.211 2.144 1.00 -0.56 C

ATOM 510 C SER A 538 -39.837 17.000 0.986 1.00-19.38 C

ATOM 511 O SER A 538 -39.176 17.845 0.377 1.00-19.38 O

ATOM 512 CB SER A 538 -39.339 16.931 3.508 1.00 -0.56 C

ATOM 513 OG SER A 538 -40.677 17.084 3.936 1.00 -0.56 O

ATOM 514 H SER A 538 -37.257 16.958 1.923 1.00 -0.56 H

ATOM 515 HA SER A 538 -39.660 15.227 2.212 1.00 -0.56 H

ATOM 516 HB2 SER A 538 -38.795 16.390 4.283 1.00 -0.56 H

ATOM 517 HB3 SER A 538 -38.905 17.929 3.462 1.00 -0.56 H

ATOM 518 HG SER A 538 -40.900 18.034 3.870 1.00 -0.56 H

ATOM 519 N ASP A 539 -41.123 16.714 0.731 1.00-19.38 N

ATOM 520 CA ASP A 539 -42.080 17.501 -0.068 1.00-19.38 C

ATOM 521 C ASP A 539 -41.617 17.845 -1.498 1.00-19.38 C

ATOM 522 O ASP A 539 -41.816 18.954 -2.001 1.00-19.38 O

ATOM 523 CB ASP A 539 -42.689 18.696 0.712 1.00-19.38 C

ATOM 524 CG ASP A 539 -43.401 18.270 1.998 1.00-19.38 C

ATOM 525 OD1 ASP A 539 -43.518 19.081 2.942 1.00-19.38 O

ATOM 526 OD2 ASP A 539 -43.917 17.136 2.068 1.00-19.38 O1-

ATOM 527 H ASP A 539 -41.573 16.028 1.324 1.00-19.38 H

ATOM 528 HA ASP A 539 -42.903 16.808 -0.240 1.00-19.38 H

ATOM 529 HB2 ASP A 539 -41.880 19.382 0.968 1.00-19.38 H

ATOM 530 HB3 ASP A 539 -43.393 19.248 0.088 1.00-19.38 H

ATOM 531 N PHE A 540 -41.015 16.841 -2.138 1.00-19.38 N

ATOM 532 CA PHE A 540 -40.564 16.839 -3.521 1.00-19.38 C

ATOM 533 H PHE A 540 -40.923 15.954 -1.664 1.00-19.38 H

ATOM 534 H01 PHE A 540 -41.203 16.182 -4.111 1.00-19.38 H

ATOM 535 H02 PHE A 540 -40.615 17.851 -3.922 1.00-19.38 H

ATOM 536 H03 PHE A 540 -39.535 16.482 -3.567 1.00-19.38 H

ATOM 537 CA GLY A 541 -42.659 18.001 -6.542 1.00 -0.28 C

ATOM 538 C GLY A 541 -44.028 18.578 -6.138 1.00 -0.28 C

ATOM 539 O GLY A 541 -44.856 18.805 -7.023 1.00 -0.28 O

ATOM 540 H01 GLY A 541 -42.726 16.915 -6.605 1.00 -0.28 H

ATOM 541 H02 GLY A 541 -42.366 18.404 -7.511 1.00 -0.28 H

ATOM 542 H03 GLY A 541 -41.915 18.276 -5.795 1.00 -0.28 H

ATOM 543 N LEU A 542 -44.275 18.827 -4.843 1.00 -0.28 N

ATOM 544 CA LEU A 542 -45.523 19.382 -4.308 1.00 -0.28 C

ATOM 545 C LEU A 542 -45.692 20.885 -4.595 1.00 -1.55 C

ATOM 546 O LEU A 542 -46.827 21.367 -4.596 1.00 -1.55 O

ATOM 547 CB LEU A 542 -45.615 19.110 -2.787 1.00 -0.28 C

ATOM 548 CG LEU A 542 -46.055 17.678 -2.411 1.00 -0.28 C

ATOM 549 CD1 LEU A 542 -45.896 17.434 -0.895 1.00 -0.28 C

ATOM 550 CD2 LEU A 542 -47.492 17.372 -2.886 1.00 -0.28 C

ATOM 551 H LEU A 542 -43.534 18.656 -4.177 1.00 -0.28 H

ATOM 552 HA LEU A 542 -46.351 18.882 -4.812 1.00 -0.28 H

ATOM 553 HB2 LEU A 542 -44.653 19.339 -2.327 1.00 -0.28 H

ATOM 554 HB3 LEU A 542 -46.323 19.804 -2.332 1.00 -0.28 H

ATOM 555 HG LEU A 542 -45.382 16.984 -2.918 1.00 -0.28 H

ATOM 556 HD11 LEU A 542 -45.229 16.593 -0.705 1.00 -0.28 H

ATOM 557 HD12 LEU A 542 -45.477 18.298 -0.381 1.00 -0.28 H

ATOM 558 HD13 LEU A 542 -46.838 17.210 -0.393 1.00 -0.28 H

ATOM 559 HD21 LEU A 542 -48.106 16.895 -2.123 1.00 -0.28 H

ATOM 560 HD22 LEU A 542 -48.024 18.273 -3.194 1.00 -0.28 H

ATOM 561 HD23 LEU A 542 -47.479 16.694 -3.738 1.00 -0.28 H

ATOM 562 N SER A 543 -44.588 21.596 -4.871 1.00 -1.55 N

ATOM 563 CA SER A 543 -44.541 23.020 -5.229 1.00 -1.55 C

ATOM 564 C SER A 543 -45.464 23.421 -6.403 1.00 -1.55 C

ATOM 565 O SER A 543 -45.919 24.564 -6.462 1.00 -1.55 O

ATOM 566 CB SER A 543 -43.072 23.409 -5.505 1.00 -1.55 C

ATOM 567 OG SER A 543 -42.475 22.548 -6.463 1.00 -1.55 O

ATOM 568 H SER A 543 -43.684 21.134 -4.821 1.00 -1.55 H

ATOM 569 HA SER A 543 -44.882 23.580 -4.356 1.00 -1.55 H

ATOM 570 HB2 SER A 543 -43.005 24.438 -5.860 1.00 -1.55 H

ATOM 571 HB3 SER A 543 -42.484 23.365 -4.589 1.00 -1.55 H

ATOM 572 HG SER A 543 -42.080 21.817 -5.954 1.00 -1.55 H

ATOM 573 N ARG A 544 -45.753 22.461 -7.291 1.00 -1.55 N

ATOM 574 CA ARG A 544 -46.653 22.587 -8.432 1.00 -1.55 C

ATOM 575 H ARG A 544 -45.343 21.550 -7.138 1.00 -1.55 H

ATOM 576 H01 ARG A 544 -46.140 23.100 -9.245 1.00 -1.55 H

ATOM 577 H02 ARG A 544 -46.960 21.595 -8.764 1.00 -1.55 H

ATOM 578 H03 ARG A 544 -47.533 23.160 -8.139 1.00 -1.55 H

ATOM 579 CA TYR A 545 -49.943 22.352 -6.437 1.00 0.04 C

ATOM 580 C TYR A 545 -50.344 23.465 -5.465 1.00 0.04 C

ATOM 581 O TYR A 545 -51.534 23.580 -5.183 1.00 0.04 O

ATOM 582 H01 TYR A 545 -50.637 21.517 -6.341 1.00 0.04 H

ATOM 583 H02 TYR A 545 -49.971 22.733 -7.458 1.00 0.04 H

ATOM 584 H03 TYR A 545 -48.934 22.013 -6.204 1.00 0.04 H

ATOM 585 N VAL A 546 -49.385 24.271 -4.994 1.00 0.04 N

ATOM 586 CA VAL A 546 -49.614 25.429 -4.131 1.00 0.04 C

ATOM 587 C VAL A 546 -50.393 26.530 -4.892 1.00 0.04 C

ATOM 588 O VAL A 546 -49.990 26.904 -5.995 1.00 0.04 O

ATOM 589 CB VAL A 546 -48.256 26.015 -3.637 1.00 0.04 C

ATOM 590 CG1 VAL A 546 -48.388 27.302 -2.797 1.00 0.04 C

ATOM 591 CG2 VAL A 546 -47.436 24.980 -2.846 1.00 0.04 C

ATOM 592 H VAL A 546 -48.439 24.140 -5.323 1.00 0.04 H

ATOM 593 HA VAL A 546 -50.201 25.106 -3.270 1.00 0.04 H

ATOM 594 HB VAL A 546 -47.662 26.271 -4.516 1.00 0.04 H

ATOM 595 HG11 VAL A 546 -47.451 27.551 -2.298 1.00 0.04 H

ATOM 596 HG12 VAL A 546 -48.642 28.158 -3.420 1.00 0.04 H

ATOM 597 HG13 VAL A 546 -49.148 27.197 -2.022 1.00 0.04 H

ATOM 598 HG21 VAL A 546 -46.455 25.378 -2.588 1.00 0.04 H

ATOM 599 HG22 VAL A 546 -47.931 24.710 -1.915 1.00 0.04 H

ATOM 600 HG23 VAL A 546 -47.265 24.064 -3.413 1.00 0.04 H

ATOM 601 N LEU A 547 -51.487 27.026 -4.298 1.00 0.04 N

ATOM 602 CA LEU A 547 -52.342 28.059 -4.901 1.00 0.04 C

ATOM 603 H LEU A 547 -51.798 26.640 -3.410 1.00 0.04 H

ATOM 604 H01 LEU A 547 -51.808 29.009 -4.918 1.00 0.04 H

ATOM 605 H02 LEU A 547 -52.601 27.769 -5.919 1.00 0.04 H

ATOM 606 H03 LEU A 547 -53.253 28.165 -4.312 1.00 0.04 H

ATOM 607 CA GLU A 550 -46.574 34.775 -3.166 1.00 -4.23 C

ATOM 608 C GLU A 550 -45.489 33.686 -3.164 1.00 -4.23 C

ATOM 609 O GLU A 550 -44.309 34.022 -3.075 1.00 -4.23 O

ATOM 610 H01 GLU A 550 -46.201 35.661 -2.653 1.00 -4.23 H

ATOM 611 H02 GLU A 550 -46.831 35.031 -4.194 1.00 -4.23 H

ATOM 612 H03 GLU A 550 -47.461 34.404 -2.652 1.00 -4.23 H

ATOM 613 N TYR A 551 -45.891 32.415 -3.271 1.00 -4.23 N

ATOM 614 CA TYR A 551 -44.974 31.291 -3.398 1.00 -4.23 C

ATOM 615 C TYR A 551 -44.615 31.023 -4.869 1.00 -4.23 C

ATOM 616 O TYR A 551 -43.441 30.799 -5.158 1.00 -4.23 O

ATOM 617 CB TYR A 551 -45.561 30.055 -2.684 1.00 -4.23 C

ATOM 618 CG TYR A 551 -44.658 28.837 -2.721 1.00 -4.23 C

ATOM 619 CD1 TYR A 551 -43.543 28.760 -1.861 1.00 -4.23 C

ATOM 620 CD2 TYR A 551 -44.895 27.806 -3.653 1.00 -4.23 C

ATOM 621 CE1 TYR A 551 -42.661 27.666 -1.944 1.00 -4.23 C

ATOM 622 CE2 TYR A 551 -44.014 26.712 -3.734 1.00 -4.23 C

ATOM 623 CZ TYR A 551 -42.892 26.645 -2.886 1.00 -4.23 C

ATOM 624 OH TYR A 551 -42.028 25.598 -2.991 1.00 -4.23 O

ATOM 625 H TYR A 551 -46.878 32.216 -3.384 1.00 -4.23 H

ATOM 626 HA TYR A 551 -44.033 31.527 -2.901 1.00 -4.23 H

ATOM 627 HB2 TYR A 551 -45.766 30.295 -1.639 1.00 -4.23 H

ATOM 628 HB3 TYR A 551 -46.523 29.790 -3.123 1.00 -4.23 H

ATOM 629 HD1 TYR A 551 -43.344 29.549 -1.149 1.00 -4.23 H

ATOM 630 HD2 TYR A 551 -45.742 27.855 -4.322 1.00 -4.23 H

ATOM 631 HE1 TYR A 551 -41.799 27.619 -1.293 1.00 -4.23 H

ATOM 632 HE2 TYR A 551 -44.202 25.935 -4.458 1.00 -4.23 H

ATOM 633 HH TYR A 551 -41.287 25.627 -2.335 1.00 -4.23 H

ATOM 634 N THR A 552 -45.605 31.031 -5.771 1.00 -4.23 N

ATOM 635 CA THR A 552 -45.416 30.565 -7.144 1.00 -4.23 C

ATOM 636 H THR A 552 -46.566 31.206 -5.497 1.00 -4.23 H

ATOM 637 H01 THR A 552 -46.023 29.676 -7.313 1.00 -4.23 H

ATOM 638 H02 THR A 552 -45.717 31.349 -7.839 1.00 -4.23 H

ATOM 639 H03 THR A 552 -44.365 30.323 -7.303 1.00 -4.23 H

TER

**TAF1 Protein**

ATOM 1 CA ASP A1524 -6.176 1.146 5.550 1.00 10.98 C

ATOM 2 C ASP A1524 -6.345 0.121 6.694 1.00 9.95 C

ATOM 3 O ASP A1524 -7.138 -0.811 6.572 1.00 10.97 O

ATOM 4 HA ASP A1524 -6.876 1.945 5.793 1.00 0.00 H

ATOM 5 H01 ASP A1524 -6.423 0.696 4.589 1.00 10.98 H

ATOM 6 H02 ASP A1524 -5.151 1.506 5.465 1.00 10.98 H

ATOM 7 N SER A1525 -5.635 0.329 7.813 1.00 9.76 N

ATOM 8 CA SER A1525 -5.688 -0.497 9.027 1.00 10.52 C

ATOM 9 C SER A1525 -6.967 -0.331 9.877 1.00 9.66 C

ATOM 10 O SER A1525 -7.120 -1.043 10.872 1.00 10.11 O

ATOM 11 CB SER A1525 -4.402 -0.263 9.848 1.00 10.93 C

ATOM 12 OG SER A1525 -4.192 1.111 10.140 1.00 10.48 O

ATOM 13 H SER A1525 -4.984 1.102 7.843 1.00 0.00 H

ATOM 14 HA SER A1525 -5.685 -1.544 8.720 1.00 0.00 H

ATOM 15 HB2 SER A1525 -4.437 -0.815 10.788 1.00 0.00 H

ATOM 16 HB3 SER A1525 -3.545 -0.651 9.299 1.00 0.00 H

ATOM 17 HG SER A1525 -3.609 1.462 9.439 1.00 0.00 H

ATOM 18 N TRP A1526 -7.882 0.561 9.465 1.00 9.65 N

ATOM 19 CA TRP A1526 -9.124 0.899 10.168 1.00 11.06 C

ATOM 20 C TRP A1526 -10.043 -0.251 10.665 1.00 9.77 C

ATOM 21 O TRP A1526 -10.668 -0.037 11.706 1.00 10.69 O

ATOM 22 CB TRP A1526 -9.925 1.979 9.400 1.00 11.98 C

ATOM 23 CG TRP A1526 -10.506 1.560 8.080 1.00 10.54 C

ATOM 24 CD1 TRP A1526 -9.939 1.766 6.870 1.00 11.04 C

ATOM 25 CD2 TRP A1526 -11.697 0.750 7.830 1.00 10.81 C

ATOM 26 CE2 TRP A1526 -11.752 0.443 6.436 1.00 10.62 C

ATOM 27 CE3 TRP A1526 -12.716 0.205 8.646 1.00 10.60 C

ATOM 28 NE1 TRP A1526 -10.674 1.116 5.899 1.00 10.65 N

ATOM 29 CZ2 TRP A1526 -12.742 -0.393 5.890 1.00 11.51 C

ATOM 30 CZ3 TRP A1526 -13.708 -0.640 8.113 1.00 11.40 C

ATOM 31 CH2 TRP A1526 -13.718 -0.949 6.739 1.00 11.48 C

ATOM 32 H TRP A1526 -7.691 1.070 8.617 1.00 0.00 H

ATOM 33 HA TRP A1526 -8.785 1.387 11.080 1.00 0.00 H

ATOM 34 HB2 TRP A1526 -10.761 2.304 10.022 1.00 0.00 H

ATOM 35 HB3 TRP A1526 -9.311 2.868 9.256 1.00 0.00 H

ATOM 36 HD1 TRP A1526 -9.032 2.332 6.710 1.00 0.00 H

ATOM 37 HE1 TRP A1526 -10.416 1.123 4.915 1.00 0.00 H

ATOM 38 HE3 TRP A1526 -12.720 0.417 9.706 1.00 0.00 H

ATOM 39 HZ2 TRP A1526 -12.750 -0.620 4.833 1.00 0.00 H

ATOM 40 HZ3 TRP A1526 -14.456 -1.060 8.768 1.00 0.00 H

ATOM 41 HH2 TRP A1526 -14.477 -1.605 6.338 1.00 0.00 H

ATOM 42 N PRO A1527 -10.106 -1.447 10.019 1.00 9.01 N

ATOM 43 CA PRO A1527 -10.917 -2.561 10.555 1.00 9.76 C

ATOM 44 C PRO A1527 -10.444 -3.096 11.920 1.00 9.66 C

ATOM 45 O PRO A1527 -11.257 -3.633 12.670 1.00 10.57 O

ATOM 46 CB PRO A1527 -10.847 -3.642 9.457 1.00 10.50 C

ATOM 47 CG PRO A1527 -10.492 -2.888 8.190 1.00 9.35 C

ATOM 48 CD PRO A1527 -9.568 -1.806 8.705 1.00 9.50 C

ATOM 49 HA PRO A1527 -11.947 -2.210 10.644 1.00 0.00 H

ATOM 50 HB2 PRO A1527 -10.066 -4.372 9.679 1.00 0.00 H

ATOM 51 HB3 PRO A1527 -11.782 -4.188 9.346 1.00 0.00 H

ATOM 52 HG2 PRO A1527 -10.028 -3.507 7.423 1.00 0.00 H

ATOM 53 HG3 PRO A1527 -11.389 -2.437 7.765 1.00 0.00 H

ATOM 54 HD2 PRO A1527 -8.572 -2.231 8.823 1.00 0.00 H

ATOM 55 HD3 PRO A1527 -9.506 -0.978 8.001 1.00 0.00 H

ATOM 56 N PHE A1528 -9.149 -2.934 12.214 1.00 9.49 N

ATOM 57 CA PHE A1528 -8.477 -3.525 13.369 1.00 10.13 C

ATOM 58 C PHE A1528 -8.275 -2.491 14.494 1.00 9.69 C

ATOM 59 O PHE A1528 -7.816 -2.863 15.573 1.00 10.08 O

ATOM 60 CB PHE A1528 -7.122 -4.095 12.887 1.00 10.32 C

ATOM 61 CG PHE A1528 -7.219 -4.967 11.641 1.00 10.86 C

ATOM 62 CD1 PHE A1528 -7.843 -6.229 11.709 1.00 11.29 C

ATOM 63 CD2 PHE A1528 -6.848 -4.460 10.376 1.00 10.99 C

ATOM 64 CE1 PHE A1528 -8.046 -6.976 10.559 1.00 11.34 C

ATOM 65 CE2 PHE A1528 -7.059 -5.220 9.234 1.00 10.92 C

ATOM 66 CZ PHE A1528 -7.654 -6.472 9.329 1.00 10.48 C

ATOM 67 H PHE A1528 -8.534 -2.482 11.547 1.00 0.00 H

ATOM 68 HA PHE A1528 -9.070 -4.349 13.770 1.00 0.00 H

ATOM 69 HB2 PHE A1528 -6.409 -3.289 12.705 1.00 0.00 H

ATOM 70 HB3 PHE A1528 -6.695 -4.695 13.691 1.00 0.00 H

ATOM 71 HD1 PHE A1528 -8.188 -6.617 12.654 1.00 0.00 H

ATOM 72 HD2 PHE A1528 -6.407 -3.479 10.291 1.00 0.00 H

ATOM 73 HE1 PHE A1528 -8.523 -7.944 10.622 1.00 0.00 H

ATOM 74 HE2 PHE A1528 -6.771 -4.833 8.268 1.00 0.00 H

ATOM 75 HZ PHE A1528 -7.822 -7.058 8.441 1.00 0.00 H

ATOM 76 N HIS A1529 -8.599 -1.212 14.232 1.00 9.64 N

ATOM 77 CA HIS A1529 -8.406 -0.086 15.156 1.00 9.71 C

ATOM 78 C HIS A1529 -9.277 -0.170 16.418 1.00 9.65 C

ATOM 79 O HIS A1529 -8.828 0.259 17.477 1.00 10.89 O

ATOM 80 CB HIS A1529 -8.680 1.263 14.453 1.00 10.37 C

ATOM 81 CG HIS A1529 -7.607 1.777 13.535 1.00 11.57 C

ATOM 82 CD2 HIS A1529 -6.509 1.131 13.013 1.00 10.57 C

ATOM 83 ND1 HIS A1529 -7.640 3.081 13.028 1.00 12.33 N

ATOM 84 CE1 HIS A1529 -6.596 3.162 12.211 1.00 12.25 C

ATOM 85 NE2 HIS A1529 -5.897 2.032 12.159 1.00 11.87 N

ATOM 86 H HIS A1529 -9.010 -1.007 13.331 1.00 0.00 H

ATOM 87 HA HIS A1529 -7.368 -0.088 15.486 1.00 0.00 H

ATOM 88 HB2 HIS A1529 -9.616 1.214 13.896 1.00 0.00 H

ATOM 89 HB3 HIS A1529 -8.829 2.043 15.202 1.00 0.00 H

ATOM 90 HD2 HIS A1529 -6.154 0.117 13.130 1.00 0.00 H

ATOM 91 HE1 HIS A1529 -6.347 4.040 11.631 1.00 0.00 H

ATOM 92 HE2 HIS A1529 -5.112 1.839 11.526 1.00 0.00 H

ATOM 93 N HIS A1530 -10.502 -0.673 16.261 1.00 8.80 N

ATOM 94 CA HIS A1530 -11.554 -0.725 17.279 1.00 9.28 C

ATOM 95 C HIS A1530 -12.222 -2.109 17.223 1.00 9.76 C

ATOM 96 O HIS A1530 -12.091 -2.761 16.184 1.00 9.94 O

ATOM 97 CB HIS A1530 -12.600 0.370 16.965 1.00 11.42 C

ATOM 98 CG HIS A1530 -12.086 1.784 17.038 1.00 12.21 C

ATOM 99 CD2 HIS A1530 -11.785 2.701 16.056 1.00 13.67 C

ATOM 100 ND1 HIS A1530 -11.818 2.422 18.235 1.00 12.61 N

ATOM 101 CE1 HIS A1530 -11.361 3.640 17.952 1.00 13.76 C

ATOM 102 NE2 HIS A1530 -11.321 3.881 16.644 1.00 14.28 N

ATOM 103 H HIS A1530 -10.744 -1.111 15.383 1.00 0.00 H

ATOM 104 HA HIS A1530 -11.119 -0.562 18.264 1.00 0.00 H

ATOM 105 HB2 HIS A1530 -13.017 0.209 15.970 1.00 0.00 H

ATOM 106 HB3 HIS A1530 -13.446 0.298 17.648 1.00 0.00 H

ATOM 107 HD1 HIS A1530 -11.913 2.024 19.203 1.00 0.00 H

ATOM 108 HD2 HIS A1530 -11.853 2.595 14.981 1.00 0.00 H

ATOM 109 HE1 HIS A1530 -11.061 4.353 18.709 1.00 0.00 H

ATOM 110 N PRO A1531 -12.980 -2.513 18.274 1.00 10.74 N

ATOM 111 CA PRO A1531 -13.785 -3.751 18.235 1.00 9.75 C

ATOM 112 C PRO A1531 -14.813 -3.757 17.090 1.00 10.06 C

ATOM 113 O PRO A1531 -15.435 -2.722 16.835 1.00 11.55 O

ATOM 114 CB PRO A1531 -14.493 -3.785 19.606 1.00 11.84 C

ATOM 115 CG PRO A1531 -13.683 -2.869 20.506 1.00 11.94 C

ATOM 116 CD PRO A1531 -13.157 -1.811 19.547 1.00 10.27 C

ATOM 117 HA PRO A1531 -13.093 -4.591 18.144 1.00 0.00 H

ATOM 118 HB2 PRO A1531 -15.510 -3.396 19.531 1.00 0.00 H

ATOM 119 HB3 PRO A1531 -14.565 -4.796 20.007 1.00 0.00 H

ATOM 120 HG2 PRO A1531 -14.254 -2.455 21.338 1.00 0.00 H

ATOM 121 HG3 PRO A1531 -12.842 -3.422 20.926 1.00 0.00 H

ATOM 122 HD2 PRO A1531 -13.894 -1.017 19.423 1.00 0.00 H

ATOM 123 HD3 PRO A1531 -12.241 -1.371 19.939 1.00 0.00 H

ATOM 124 N VAL A1532 -15.000 -4.913 16.443 1.00 10.59 N

ATOM 125 CA VAL A1532 -16.036 -5.108 15.431 1.00 10.66 C

ATOM 126 C VAL A1532 -17.434 -4.976 16.072 1.00 10.33 C

ATOM 127 O VAL A1532 -17.701 -5.604 17.099 1.00 11.31 O

ATOM 128 CB VAL A1532 -15.939 -6.506 14.755 1.00 10.42 C

ATOM 129 CG1 VAL A1532 -17.012 -6.745 13.675 1.00 11.19 C

ATOM 130 CG2 VAL A1532 -14.549 -6.741 14.146 1.00 11.88 C

ATOM 131 H VAL A1532 -14.440 -5.722 16.670 1.00 0.00 H

ATOM 132 HA VAL A1532 -15.905 -4.338 14.669 1.00 0.00 H

ATOM 133 HB VAL A1532 -16.084 -7.278 15.514 1.00 0.00 H

ATOM 134 HG11 VAL A1532 -16.832 -7.679 13.144 1.00 0.00 H

ATOM 135 HG12 VAL A1532 -18.015 -6.813 14.095 1.00 0.00 H

ATOM 136 HG13 VAL A1532 -17.013 -5.944 12.934 1.00 0.00 H

ATOM 137 HG21 VAL A1532 -14.520 -7.646 13.541 1.00 0.00 H

ATOM 138 HG22 VAL A1532 -14.245 -5.910 13.512 1.00 0.00 H

ATOM 139 HG23 VAL A1532 -13.793 -6.859 14.923 1.00 0.00 H

ATOM 140 N ASN A1533 -18.298 -4.161 15.473 1.00 9.84 N

ATOM 141 CA ASN A1533 -19.688 -4.023 15.877 1.00 11.32 C

ATOM 142 C ASN A1533 -20.514 -5.049 15.090 1.00 11.78 C

ATOM 143 O ASN A1533 -20.457 -5.070 13.857 1.00 11.00 O

ATOM 144 CB ASN A1533 -20.173 -2.584 15.598 1.00 12.34 C

ATOM 145 CG ASN A1533 -21.461 -2.214 16.342 1.00 13.36 C

ATOM 146 ND2 ASN A1533 -21.610 -0.951 16.720 1.00 15.76 N

ATOM 147 OD1 ASN A1533 -22.332 -3.051 16.568 1.00 14.79 O

ATOM 148 H ASN A1533 -18.021 -3.678 14.616 1.00 0.00 H

ATOM 149 HA ASN A1533 -19.826 -4.237 16.940 1.00 0.00 H

ATOM 150 HB2 ASN A1533 -19.399 -1.884 15.916 1.00 0.00 H

ATOM 151 HB3 ASN A1533 -20.312 -2.425 14.530 1.00 0.00 H

ATOM 152 HD21 ASN A1533 -20.906 -0.256 16.516 1.00 0.00 H

ATOM 153 HD22 ASN A1533 -22.440 -0.658 17.214 1.00 0.00 H

ATOM 154 N LYS A1534 -21.312 -5.850 15.805 1.00 12.28 N

ATOM 155 CA LYS A1534 -22.236 -6.827 15.228 1.00 12.54 C

ATOM 156 C LYS A1534 -23.340 -6.182 14.363 1.00 12.39 C

ATOM 157 O LYS A1534 -23.893 -6.854 13.497 1.00 13.31 O

ATOM 158 H LYS A1534 -21.331 -5.755 16.810 1.00 0.00 H

ATOM 159 H01 LYS A1534 -22.709 -7.379 16.040 1.00 12.54 H

ATOM 160 H02 LYS A1534 -21.653 -7.478 14.577 1.00 12.54 H

ATOM 161 N LYS A1535 -23.630 -4.888 14.570 1.00 11.80 N

ATOM 162 CA LYS A1535 -24.524 -4.118 13.702 1.00 13.19 C

ATOM 163 C LYS A1535 -23.929 -3.878 12.298 1.00 13.30 C

ATOM 164 O LYS A1535 -24.694 -3.677 11.354 1.00 14.61 O

ATOM 165 H LYS A1535 -23.154 -4.375 15.306 1.00 0.00 H

ATOM 166 H01 LYS A1535 -24.718 -3.152 14.169 1.00 13.19 H

ATOM 167 H02 LYS A1535 -25.440 -4.696 13.577 1.00 13.19 H

ATOM 168 N PHE A1536 -22.594 -3.893 12.185 1.00 12.42 N

ATOM 169 CA PHE A1536 -21.869 -3.609 10.947 1.00 11.58 C

ATOM 170 C PHE A1536 -21.345 -4.902 10.296 1.00 12.08 C

ATOM 171 O PHE A1536 -21.318 -4.970 9.067 1.00 13.02 O

ATOM 172 CB PHE A1536 -20.734 -2.596 11.225 1.00 12.39 C

ATOM 173 CG PHE A1536 -21.147 -1.275 11.876 1.00 11.42 C

ATOM 174 CD1 PHE A1536 -22.414 -0.688 11.642 1.00 12.74 C

ATOM 175 CD2 PHE A1536 -20.197 -0.537 12.614 1.00 12.59 C

ATOM 176 CE1 PHE A1536 -22.741 0.527 12.226 1.00 13.77 C

ATOM 177 CE2 PHE A1536 -20.537 0.683 13.183 1.00 14.16 C

ATOM 178 CZ PHE A1536 -21.810 1.206 13.001 1.00 14.11 C

ATOM 179 H PHE A1536 -22.024 -4.108 12.994 1.00 0.00 H

ATOM 180 HA PHE A1536 -22.539 -3.159 10.213 1.00 0.00 H

ATOM 181 HB2 PHE A1536 -19.981 -3.067 11.861 1.00 0.00 H

ATOM 182 HB3 PHE A1536 -20.223 -2.364 10.290 1.00 0.00 H

ATOM 183 HD1 PHE A1536 -23.155 -1.183 11.032 1.00 0.00 H

ATOM 184 HD2 PHE A1536 -19.203 -0.927 12.763 1.00 0.00 H

ATOM 185 HE1 PHE A1536 -23.722 0.953 12.069 1.00 0.00 H

ATOM 186 HE2 PHE A1536 -19.808 1.227 13.767 1.00 0.00 H

ATOM 187 HZ PHE A1536 -22.071 2.155 13.449 1.00 0.00 H

ATOM 188 N VAL A1537 -20.991 -5.918 11.098 1.00 11.42 N

ATOM 189 CA VAL A1537 -20.587 -7.244 10.619 1.00 12.31 C

ATOM 190 C VAL A1537 -21.333 -8.325 11.454 1.00 12.18 C

ATOM 191 O VAL A1537 -20.816 -8.753 12.490 1.00 11.21 O

ATOM 192 CB VAL A1537 -19.047 -7.463 10.722 1.00 10.87 C

ATOM 193 CG1 VAL A1537 -18.590 -8.763 10.029 1.00 13.38 C

ATOM 194 CG2 VAL A1537 -18.234 -6.288 10.150 1.00 12.22 C

ATOM 195 H VAL A1537 -20.970 -5.780 12.105 1.00 0.00 H

ATOM 196 HA VAL A1537 -20.844 -7.372 9.567 1.00 0.00 H

ATOM 197 HB VAL A1537 -18.773 -7.543 11.773 1.00 0.00 H

ATOM 198 HG11 VAL A1537 -17.518 -8.918 10.153 1.00 0.00 H

ATOM 199 HG12 VAL A1537 -19.089 -9.647 10.426 1.00 0.00 H

ATOM 200 HG13 VAL A1537 -18.791 -8.732 8.958 1.00 0.00 H

ATOM 201 HG21 VAL A1537 -17.167 -6.504 10.192 1.00 0.00 H

ATOM 202 HG22 VAL A1537 -18.493 -6.087 9.111 1.00 0.00 H

ATOM 203 HG23 VAL A1537 -18.388 -5.371 10.719 1.00 0.00 H

ATOM 204 N PRO A1538 -22.558 -8.720 11.025 1.00 13.05 N

ATOM 205 CA PRO A1538 -23.432 -9.640 11.790 1.00 12.76 C

ATOM 206 C PRO A1538 -22.842 -10.993 12.223 1.00 12.40 C

ATOM 207 O PRO A1538 -23.067 -11.409 13.360 1.00 13.36 O

ATOM 208 CB PRO A1538 -24.664 -9.818 10.884 1.00 16.65 C

ATOM 209 CG PRO A1538 -24.723 -8.536 10.070 1.00 15.07 C

ATOM 210 CD PRO A1538 -23.255 -8.211 9.838 1.00 13.90 C

ATOM 211 HA PRO A1538 -23.740 -9.123 12.695 1.00 0.00 H

ATOM 212 HB2 PRO A1538 -24.533 -10.669 10.212 1.00 0.00 H

ATOM 213 HB3 PRO A1538 -25.578 -9.990 11.452 1.00 0.00 H

ATOM 214 HG2 PRO A1538 -25.299 -8.629 9.148 1.00 0.00 H

ATOM 215 HG3 PRO A1538 -25.178 -7.747 10.672 1.00 0.00 H

ATOM 216 HD2 PRO A1538 -22.877 -8.732 8.957 1.00 0.00 H

ATOM 217 HD3 PRO A1538 -23.134 -7.140 9.680 1.00 0.00 H

ATOM 218 N ASP A1539 -22.129 -11.667 11.314 1.00 12.31 N

ATOM 219 CA ASP A1539 -21.695 -13.060 11.490 1.00 13.72 C

ATOM 220 C ASP A1539 -20.302 -13.199 12.125 1.00 12.43 C

ATOM 221 O ASP A1539 -19.845 -14.329 12.288 1.00 12.72 O

ATOM 222 CB ASP A1539 -21.796 -13.907 10.192 1.00 16.15 C

ATOM 223 CG ASP A1539 -23.190 -13.988 9.570 1.00 21.66 C

ATOM 224 OD1 ASP A1539 -24.190 -13.977 10.321 1.00 23.07 O

ATOM 225 OD2 ASP A1539 -23.265 -14.194 8.343 1.00 27.87 O1-

ATOM 226 H ASP A1539 -21.983 -11.261 10.401 1.00 0.00 H

ATOM 227 HA ASP A1539 -22.366 -13.533 12.207 1.00 0.00 H

ATOM 228 HB2 ASP A1539 -21.116 -13.480 9.455 1.00 0.00 H

ATOM 229 HB3 ASP A1539 -21.463 -14.929 10.377 1.00 0.00 H

ATOM 230 N TYR A1540 -19.642 -12.088 12.488 1.00 12.50 N

ATOM 231 CA TYR A1540 -18.263 -12.076 12.999 1.00 11.29 C

ATOM 232 C TYR A1540 -18.069 -12.982 14.232 1.00 10.62 C

ATOM 233 O TYR A1540 -17.258 -13.909 14.208 1.00 10.90 O

ATOM 234 CB TYR A1540 -17.811 -10.629 13.296 1.00 11.38 C

ATOM 235 CG TYR A1540 -16.317 -10.494 13.523 1.00 10.77 C

ATOM 236 CD1 TYR A1540 -15.457 -10.445 12.408 1.00 11.17 C

ATOM 237 CD2 TYR A1540 -15.776 -10.447 14.826 1.00 10.66 C

ATOM 238 CE1 TYR A1540 -14.065 -10.369 12.588 1.00 11.40 C

ATOM 239 CE2 TYR A1540 -14.380 -10.369 15.006 1.00 11.75 C

ATOM 240 CZ TYR A1540 -13.525 -10.336 13.887 1.00 10.93 C

ATOM 241 OH TYR A1540 -12.174 -10.285 14.061 1.00 11.38 O

ATOM 242 H TYR A1540 -20.092 -11.192 12.361 1.00 0.00 H

ATOM 243 HA TYR A1540 -17.626 -12.472 12.207 1.00 0.00 H

ATOM 244 HB2 TYR A1540 -18.060 -9.987 12.454 1.00 0.00 H

ATOM 245 HB3 TYR A1540 -18.352 -10.214 14.147 1.00 0.00 H

ATOM 246 HD1 TYR A1540 -15.861 -10.478 11.407 1.00 0.00 H

ATOM 247 HD2 TYR A1540 -16.424 -10.484 15.691 1.00 0.00 H

ATOM 248 HE1 TYR A1540 -13.426 -10.340 11.718 1.00 0.00 H

ATOM 249 HE2 TYR A1540 -13.968 -10.344 16.005 1.00 0.00 H

ATOM 250 HH TYR A1540 -11.684 -10.321 13.212 1.00 0.00 H

ATOM 251 N TYR A1541 -18.888 -12.743 15.261 1.00 11.33 N

ATOM 252 CA TYR A1541 -18.820 -13.423 16.556 1.00 12.61 C

ATOM 253 C TYR A1541 -19.377 -14.859 16.542 1.00 13.02 C

ATOM 254 O TYR A1541 -19.246 -15.565 17.542 1.00 14.26 O

ATOM 255 CB TYR A1541 -19.506 -12.541 17.617 1.00 12.46 C

ATOM 256 CG TYR A1541 -18.792 -11.218 17.838 1.00 11.02 C

ATOM 257 CD1 TYR A1541 -17.630 -11.187 18.637 1.00 11.52 C

ATOM 258 CD2 TYR A1541 -19.251 -10.032 17.225 1.00 10.83 C

ATOM 259 CE1 TYR A1541 -16.934 -9.979 18.818 1.00 10.85 C

ATOM 260 CE2 TYR A1541 -18.544 -8.825 17.403 1.00 10.65 C

ATOM 261 CZ TYR A1541 -17.382 -8.801 18.201 1.00 10.46 C

ATOM 262 OH TYR A1541 -16.679 -7.649 18.395 1.00 11.54 O

ATOM 263 H TYR A1541 -19.557 -11.992 15.189 1.00 0.00 H

ATOM 264 HA TYR A1541 -17.766 -13.507 16.826 1.00 0.00 H

ATOM 265 HB2 TYR A1541 -20.549 -12.362 17.350 1.00 0.00 H

ATOM 266 HB3 TYR A1541 -19.532 -13.066 18.574 1.00 0.00 H

ATOM 267 HD1 TYR A1541 -17.261 -12.089 19.104 1.00 0.00 H

ATOM 268 HD2 TYR A1541 -20.137 -10.041 16.607 1.00 0.00 H

ATOM 269 HE1 TYR A1541 -16.048 -9.950 19.429 1.00 0.00 H

ATOM 270 HE2 TYR A1541 -18.903 -7.927 16.920 1.00 0.00 H

ATOM 271 HH TYR A1541 -17.078 -6.883 17.933 1.00 0.00 H

ATOM 272 N LYS A1542 -19.970 -15.286 15.418 1.00 12.76 N

ATOM 273 CA LYS A1542 -20.361 -16.677 15.186 1.00 14.33 C

ATOM 274 C LYS A1542 -19.137 -17.531 14.793 1.00 14.47 C

ATOM 275 O LYS A1542 -19.116 -18.726 15.088 1.00 18.86 O

ATOM 276 CB LYS A1542 -21.443 -16.748 14.086 1.00 14.87 C

ATOM 277 CG LYS A1542 -22.692 -15.891 14.371 1.00 15.76 C

ATOM 278 CD LYS A1542 -23.716 -15.955 13.227 1.00 17.05 C

ATOM 279 CE LYS A1542 -24.890 -14.975 13.417 1.00 19.42 C

ATOM 280 NZ LYS A1542 -25.720 -14.875 12.207 1.00 23.83 N1+

ATOM 281 H LYS A1542 -20.015 -14.665 14.622 1.00 0.00 H

ATOM 282 HA LYS A1542 -20.781 -17.093 16.104 1.00 0.00 H

ATOM 283 HB2 LYS A1542 -21.011 -16.448 13.131 1.00 0.00 H

ATOM 284 HB3 LYS A1542 -21.746 -17.788 13.955 1.00 0.00 H

ATOM 285 HG2 LYS A1542 -23.157 -16.218 15.302 1.00 0.00 H

ATOM 286 HG3 LYS A1542 -22.402 -14.851 14.524 1.00 0.00 H

ATOM 287 HD2 LYS A1542 -23.212 -15.760 12.280 1.00 0.00 H

ATOM 288 HD3 LYS A1542 -24.105 -16.972 13.153 1.00 0.00 H

ATOM 289 HE2 LYS A1542 -25.508 -15.279 14.262 1.00 0.00 H

ATOM 290 HE3 LYS A1542 -24.512 -13.976 13.642 1.00 0.00 H

ATOM 291 HZ1 LYS A1542 -25.141 -14.520 11.425 1.00 0.00 H

ATOM 292 HZ2 LYS A1542 -26.101 -15.764 11.943 1.00 0.00 H

ATOM 293 HZ3 LYS A1542 -26.473 -14.226 12.345 1.00 0.00 H

ATOM 294 N VAL A1543 -18.149 -16.904 14.141 1.00 13.69 N

ATOM 295 CA VAL A1543 -16.920 -17.540 13.674 1.00 14.11 C

ATOM 296 H VAL A1543 -18.231 -15.913 13.954 1.00 0.00 H

ATOM 297 HA VAL A1543 -17.062 -18.620 13.591 1.00 0.00 H

ATOM 298 H01 VAL A1543 -16.116 -17.335 14.381 1.00 14.11 H

ATOM 299 H02 VAL A1543 -16.663 -17.139 12.694 1.00 14.11 H

ATOM 300 CA PRO A1547 -12.007 -11.320 19.480 1.00 12.04 C

ATOM 301 C PRO A1547 -10.860 -10.495 20.099 1.00 11.17 C

ATOM 302 O PRO A1547 -10.690 -10.504 21.321 1.00 11.94 O

ATOM 303 HA PRO A1547 -11.797 -11.490 18.424 1.00 0.00 H

ATOM 304 H01 PRO A1547 -12.945 -10.775 19.583 1.00 12.04 H

ATOM 305 H02 PRO A1547 -12.089 -12.277 19.995 1.00 12.04 H

ATOM 306 N MET A1548 -10.111 -9.785 19.251 1.00 10.58 N

ATOM 307 CA MET A1548 -9.041 -8.871 19.648 1.00 10.55 C

ATOM 308 C MET A1548 -8.895 -7.752 18.606 1.00 10.83 C

ATOM 309 O MET A1548 -9.276 -7.939 17.449 1.00 12.10 O

ATOM 310 H MET A1548 -10.321 -9.805 18.252 1.00 0.00 H

ATOM 311 H01 MET A1548 -8.103 -9.422 19.722 1.00 10.55 H

ATOM 312 H02 MET A1548 -9.286 -8.431 20.615 1.00 10.55 H

ATOM 313 N ASP A1549 -8.352 -6.610 19.028 1.00 10.01 N

ATOM 314 CA ASP A1549 -8.282 -5.369 18.258 1.00 10.23 C

ATOM 315 C ASP A1549 -7.228 -4.469 18.925 1.00 10.47 C

ATOM 316 O ASP A1549 -6.835 -4.716 20.070 1.00 11.06 O

ATOM 317 CB ASP A1549 -9.640 -4.621 18.182 1.00 11.34 C

ATOM 318 CG ASP A1549 -10.195 -4.188 19.536 1.00 10.29 C

ATOM 319 OD1 ASP A1549 -10.839 -5.006 20.222 1.00 11.16 O

ATOM 320 OD2 ASP A1549 -9.935 -3.034 19.922 1.00 11.00 O1-

ATOM 321 H ASP A1549 -7.993 -6.529 19.973 1.00 0.00 H

ATOM 322 HA ASP A1549 -7.945 -5.599 17.246 1.00 0.00 H

ATOM 323 HB2 ASP A1549 -9.553 -3.740 17.544 1.00 0.00 H

ATOM 324 HB3 ASP A1549 -10.389 -5.227 17.672 1.00 0.00 H

ATOM 325 N LEU A1550 -6.801 -3.427 18.204 1.00 10.11 N

ATOM 326 CA LEU A1550 -5.788 -2.481 18.660 1.00 10.79 C

ATOM 327 H LEU A1550 -7.187 -3.263 17.275 1.00 0.00 H

ATOM 328 HA LEU A1550 -4.941 -3.072 19.011 1.00 0.00 H

ATOM 329 H01 LEU A1550 -6.182 -1.870 19.472 1.00 10.79 H

ATOM 330 H02 LEU A1550 -5.490 -1.809 17.855 1.00 10.79 H

ATOM 331 CA LEU A1574 -4.660 -12.855 17.523 1.00 10.38 C

ATOM 332 C LEU A1574 -5.501 -12.074 16.491 1.00 10.78 C

ATOM 333 O LEU A1574 -6.354 -12.674 15.843 1.00 11.42 O

ATOM 334 HA LEU A1574 -4.700 -13.906 17.234 1.00 0.00 H

ATOM 335 H01 LEU A1574 -5.075 -12.724 18.522 1.00 10.38 H

ATOM 336 H02 LEU A1574 -3.632 -12.494 17.541 1.00 10.38 H

ATOM 337 N ILE A1575 -5.181 -10.787 16.280 1.00 10.98 N

ATOM 338 CA ILE A1575 -5.766 -9.936 15.235 1.00 11.79 C

ATOM 339 C ILE A1575 -5.552 -10.525 13.819 1.00 11.80 C

ATOM 340 O ILE A1575 -6.505 -10.632 13.043 1.00 12.57 O

ATOM 341 CB ILE A1575 -5.189 -8.483 15.300 1.00 11.09 C

ATOM 342 CG1 ILE A1575 -5.611 -7.792 16.617 1.00 12.27 C

ATOM 343 CG2 ILE A1575 -5.543 -7.578 14.097 1.00 13.59 C

ATOM 344 CD1 ILE A1575 -4.893 -6.464 16.896 1.00 12.98 C

ATOM 345 H ILE A1575 -4.446 -10.371 16.837 1.00 0.00 H

ATOM 346 HA ILE A1575 -6.843 -9.893 15.415 1.00 0.00 H

ATOM 347 HB ILE A1575 -4.104 -8.562 15.308 1.00 0.00 H

ATOM 348 HG12 ILE A1575 -6.686 -7.616 16.594 1.00 0.00 H

ATOM 349 HG13 ILE A1575 -5.438 -8.452 17.466 1.00 0.00 H

ATOM 350 HG21 ILE A1575 -5.113 -6.582 14.197 1.00 0.00 H

ATOM 351 HG22 ILE A1575 -5.173 -7.965 13.148 1.00 0.00 H

ATOM 352 HG23 ILE A1575 -6.622 -7.465 14.013 1.00 0.00 H

ATOM 353 HD11 ILE A1575 -4.990 -6.190 17.945 1.00 0.00 H

ATOM 354 HD12 ILE A1575 -3.829 -6.525 16.666 1.00 0.00 H

ATOM 355 HD13 ILE A1575 -5.323 -5.650 16.313 1.00 0.00 H

ATOM 356 N LEU A1576 -4.312 -10.939 13.526 1.00 11.39 N

ATOM 357 CA LEU A1576 -3.932 -11.602 12.279 1.00 10.66 C

ATOM 358 H LEU A1576 -3.594 -10.847 14.231 1.00 0.00 H

ATOM 359 H01 LEU A1576 -4.005 -10.894 11.453 1.00 10.66 H

ATOM 360 H02 LEU A1576 -4.599 -12.444 12.097 1.00 10.66 H

ATOM 361 H03 LEU A1576 -2.906 -11.962 12.358 1.00 10.66 H

ATOM 362 CA ALA A1577 -5.192 -15.124 13.206 1.00 11.01 C

ATOM 363 C ALA A1577 -6.714 -15.096 12.979 1.00 10.73 C

ATOM 364 O ALA A1577 -7.231 -15.945 12.252 1.00 11.85 O

ATOM 365 HA ALA A1577 -4.749 -15.711 12.400 1.00 0.00 H

ATOM 366 H01 ALA A1577 -4.967 -15.584 14.168 1.00 11.01 H

ATOM 367 H02 ALA A1577 -4.787 -14.112 13.209 1.00 11.01 H

ATOM 368 N ASN A1578 -7.400 -14.106 13.561 1.00 10.03 N

ATOM 369 CA ASN A1578 -8.839 -13.890 13.375 1.00 10.46 C

ATOM 370 C ASN A1578 -9.167 -13.413 11.952 1.00 10.64 C

ATOM 371 O ASN A1578 -10.238 -13.748 11.445 1.00 10.89 O

ATOM 372 CB ASN A1578 -9.393 -12.910 14.436 1.00 10.63 C

ATOM 373 CG ASN A1578 -9.452 -13.482 15.856 1.00 10.84 C

ATOM 374 ND2 ASN A1578 -9.739 -12.640 16.838 1.00 11.98 N

ATOM 375 OD1 ASN A1578 -9.273 -14.679 16.081 1.00 12.28 O

ATOM 376 H ASN A1578 -6.923 -13.479 14.206 1.00 0.00 H

ATOM 377 HA ASN A1578 -9.311 -14.862 13.527 1.00 0.00 H

ATOM 378 HB2 ASN A1578 -8.823 -11.979 14.430 1.00 0.00 H

ATOM 379 HB3 ASN A1578 -10.418 -12.641 14.173 1.00 0.00 H

ATOM 380 HD21 ASN A1578 -9.997 -11.666 16.640 1.00 0.00 H

ATOM 381 HD22 ASN A1578 -9.806 -12.978 17.796 1.00 0.00 H

ATOM 382 N SER A1579 -8.255 -12.687 11.291 1.00 10.64 N

ATOM 383 CA SER A1579 -8.415 -12.333 9.882 1.00 10.78 C

ATOM 384 C SER A1579 -8.223 -13.564 8.977 1.00 11.10 C

ATOM 385 O SER A1579 -9.042 -13.775 8.083 1.00 11.23 O

ATOM 386 CB SER A1579 -7.520 -11.140 9.494 1.00 11.39 C

ATOM 387 OG SER A1579 -7.883 -10.640 8.217 1.00 11.13 O

ATOM 388 H SER A1579 -7.383 -12.434 11.740 1.00 0.00 H

ATOM 389 HA SER A1579 -9.447 -12.001 9.752 1.00 0.00 H

ATOM 390 HB2 SER A1579 -7.624 -10.333 10.222 1.00 0.00 H

ATOM 391 HB3 SER A1579 -6.467 -11.425 9.484 1.00 0.00 H

ATOM 392 HG SER A1579 -7.488 -11.217 7.530 1.00 0.00 H

ATOM 393 N VAL A1580 -7.212 -14.403 9.258 1.00 11.38 N

ATOM 394 CA VAL A1580 -7.001 -15.678 8.566 1.00 12.35 C

ATOM 395 H VAL A1580 -6.558 -14.164 9.998 1.00 0.00 H

ATOM 396 HA VAL A1580 -6.865 -15.446 7.508 1.00 0.00 H

ATOM 397 H01 VAL A1580 -7.867 -16.326 8.700 1.00 12.35 H

ATOM 398 H02 VAL A1580 -6.131 -16.199 8.966 1.00 12.35 H

ATOM 399 CA LYS A1581 -10.064 -17.422 10.151 1.00 13.76 C

ATOM 400 C LYS A1581 -11.284 -16.839 9.413 1.00 12.65 C

ATOM 401 O LYS A1581 -11.930 -17.571 8.662 1.00 15.25 O

ATOM 402 HA LYS A1581 -9.925 -18.441 9.782 1.00 0.00 H

ATOM 403 H01 LYS A1581 -10.246 -17.434 11.226 1.00 13.76 H

ATOM 404 H02 LYS A1581 -9.175 -16.818 9.970 1.00 13.76 H

ATOM 405 N TYR A1582 -11.611 -15.564 9.667 1.00 12.37 N

ATOM 406 CA TYR A1582 -12.857 -14.962 9.187 1.00 12.30 C

ATOM 407 C TYR A1582 -12.819 -14.612 7.688 1.00 11.51 C

ATOM 408 O TYR A1582 -13.776 -14.904 6.969 1.00 12.66 O

ATOM 409 CB TYR A1582 -13.246 -13.742 10.060 1.00 12.11 C

ATOM 410 CG TYR A1582 -14.616 -13.162 9.743 1.00 11.73 C

ATOM 411 CD1 TYR A1582 -15.773 -13.922 10.008 1.00 12.83 C

ATOM 412 CD2 TYR A1582 -14.744 -11.892 9.145 1.00 12.78 C

ATOM 413 CE1 TYR A1582 -17.043 -13.447 9.632 1.00 14.43 C

ATOM 414 CE2 TYR A1582 -16.016 -11.411 8.773 1.00 16.07 C

ATOM 415 CZ TYR A1582 -17.163 -12.196 8.998 1.00 15.48 C

ATOM 416 OH TYR A1582 -18.384 -11.749 8.587 1.00 20.55 O

ATOM 417 H TYR A1582 -11.038 -14.999 10.289 1.00 0.00 H

ATOM 418 HA TYR A1582 -13.646 -15.705 9.315 1.00 0.00 H

ATOM 419 HB2 TYR A1582 -13.255 -14.031 11.110 1.00 0.00 H

ATOM 420 HB3 TYR A1582 -12.490 -12.960 9.969 1.00 0.00 H

ATOM 421 HD1 TYR A1582 -15.686 -14.893 10.470 1.00 0.00 H

ATOM 422 HD2 TYR A1582 -13.868 -11.289 8.951 1.00 0.00 H

ATOM 423 HE1 TYR A1582 -17.914 -14.059 9.826 1.00 0.00 H

ATOM 424 HE2 TYR A1582 -16.105 -10.443 8.300 1.00 0.00 H

ATOM 425 HH TYR A1582 -19.074 -12.417 8.675 1.00 0.00 H

ATOM 426 N ASN A1583 -11.721 -13.995 7.238 1.00 12.05 N

ATOM 427 CA ASN A1583 -11.610 -13.406 5.898 1.00 12.20 C

ATOM 428 CB ASN A1583 -10.833 -12.070 5.945 1.00 11.86 C

ATOM 429 CG ASN A1583 -11.470 -11.033 6.861 1.00 11.50 C

ATOM 430 ND2 ASN A1583 -10.714 -10.462 7.788 1.00 11.15 N

ATOM 431 OD1 ASN A1583 -12.655 -10.741 6.730 1.00 12.41 O

ATOM 432 H ASN A1583 -10.917 -13.865 7.847 1.00 0.00 H

ATOM 433 HB2 ASN A1583 -9.786 -12.225 6.208 1.00 0.00 H

ATOM 434 HB3 ASN A1583 -10.834 -11.635 4.949 1.00 0.00 H

ATOM 435 HD21 ASN A1583 -9.712 -10.639 7.872 1.00 0.00 H

ATOM 436 HD22 ASN A1583 -11.125 -9.801 8.453 1.00 0.00 H

ATOM 437 H01 ASN A1583 -12.610 -13.225 5.503 1.00 12.20 H

ATOM 438 H02 ASN A1583 -11.069 -14.101 5.256 1.00 12.20 H

ATOM 439 CA GLU A1586 -5.289 -14.150 0.948 1.00 15.47 C

ATOM 440 C GLU A1586 -6.215 -12.932 0.808 1.00 16.35 C

ATOM 441 O GLU A1586 -6.144 -12.241 -0.209 1.00 17.33 O

ATOM 442 HA GLU A1586 -4.306 -13.713 1.123 1.00 0.00 H

ATOM 443 H01 GLU A1586 -5.292 -14.747 0.036 1.00 15.47 H

ATOM 444 H02 GLU A1586 -5.598 -14.823 1.748 1.00 15.47 H

ATOM 445 N SER A1587 -7.055 -12.655 1.811 1.00 14.39 N

ATOM 446 CA SER A1587 -7.859 -11.436 1.842 1.00 12.55 C

ATOM 447 C SER A1587 -6.968 -10.198 2.072 1.00 12.87 C

ATOM 448 O SER A1587 -5.950 -10.293 2.765 1.00 12.41 O

ATOM 449 CB SER A1587 -8.933 -11.577 2.933 1.00 12.98 C

ATOM 450 OG SER A1587 -9.841 -10.489 2.906 1.00 13.23 O

ATOM 451 H SER A1587 -7.040 -13.226 2.644 1.00 0.00 H

ATOM 452 HA SER A1587 -8.358 -11.334 0.876 1.00 0.00 H

ATOM 453 HB2 SER A1587 -9.494 -12.498 2.781 1.00 0.00 H

ATOM 454 HB3 SER A1587 -8.478 -11.643 3.922 1.00 0.00 H

ATOM 455 HG SER A1587 -10.538 -10.696 2.267 1.00 0.00 H

ATOM 456 N GLN A1588 -7.383 -9.046 1.528 1.00 12.10 N

ATOM 457 CA GLN A1588 -6.703 -7.764 1.719 1.00 13.00 C

ATOM 458 C GLN A1588 -6.731 -7.306 3.193 1.00 11.09 C

ATOM 459 O GLN A1588 -5.772 -6.679 3.643 1.00 12.21 O

ATOM 460 CB GLN A1588 -7.301 -6.711 0.756 1.00 15.00 C

ATOM 461 CG GLN A1588 -6.577 -5.344 0.714 1.00 17.09 C

ATOM 462 CD GLN A1588 -5.082 -5.454 0.402 1.00 17.60 C

ATOM 463 NE2 GLN A1588 -4.738 -5.955 -0.777 1.00 17.67 N

ATOM 464 OE1 GLN A1588 -4.238 -5.111 1.223 1.00 20.27 O

ATOM 465 H GLN A1588 -8.244 -9.032 1.001 1.00 0.00 H

ATOM 466 HA GLN A1588 -5.659 -7.931 1.451 1.00 0.00 H

ATOM 467 HB2 GLN A1588 -7.304 -7.123 -0.254 1.00 0.00 H

ATOM 468 HB3 GLN A1588 -8.349 -6.542 1.007 1.00 0.00 H

ATOM 469 HG2 GLN A1588 -7.043 -4.701 -0.034 1.00 0.00 H

ATOM 470 HG3 GLN A1588 -6.698 -4.826 1.666 1.00 0.00 H

ATOM 471 HE21 GLN A1588 -5.436 -6.224 -1.456 1.00 0.00 H

ATOM 472 HE22 GLN A1588 -3.765 -6.041 -1.033 1.00 0.00 H

ATOM 473 N TYR A1589 -7.776 -7.698 3.941 1.00 11.41 N

ATOM 474 CA TYR A1589 -7.838 -7.574 5.401 1.00 11.00 C

ATOM 475 C TYR A1589 -6.634 -8.268 6.066 1.00 10.62 C

ATOM 476 O TYR A1589 -5.904 -7.636 6.827 1.00 11.11 O

ATOM 477 CB TYR A1589 -9.165 -8.161 5.934 1.00 11.41 C

ATOM 478 CG TYR A1589 -10.424 -7.371 5.610 1.00 10.77 C

ATOM 479 CD1 TYR A1589 -10.679 -6.151 6.270 1.00 10.59 C

ATOM 480 CD2 TYR A1589 -11.371 -7.872 4.692 1.00 11.35 C

ATOM 481 CE1 TYR A1589 -11.858 -5.429 5.996 1.00 11.85 C

ATOM 482 CE2 TYR A1589 -12.551 -7.153 4.422 1.00 11.09 C

ATOM 483 CZ TYR A1589 -12.792 -5.925 5.068 1.00 11.36 C

ATOM 484 OH TYR A1589 -13.924 -5.213 4.797 1.00 12.24 O

ATOM 485 H TYR A1589 -8.523 -8.222 3.504 1.00 0.00 H

ATOM 486 HA TYR A1589 -7.795 -6.512 5.652 1.00 0.00 H

ATOM 487 HB2 TYR A1589 -9.282 -9.190 5.590 1.00 0.00 H

ATOM 488 HB3 TYR A1589 -9.120 -8.230 7.021 1.00 0.00 H

ATOM 489 HD1 TYR A1589 -9.976 -5.773 6.998 1.00 0.00 H

ATOM 490 HD2 TYR A1589 -11.208 -8.819 4.202 1.00 0.00 H

ATOM 491 HE1 TYR A1589 -12.052 -4.498 6.508 1.00 0.00 H

ATOM 492 HE2 TYR A1589 -13.264 -7.556 3.716 1.00 0.00 H

ATOM 493 HH TYR A1589 -14.524 -5.678 4.199 1.00 0.00 H

ATOM 494 N THR A1590 -6.394 -9.528 5.696 1.00 10.48 N

ATOM 495 CA THR A1590 -5.315 -10.348 6.227 1.00 10.99 C

ATOM 496 H THR A1590 -7.010 -9.999 5.052 1.00 0.00 H

ATOM 497 HA THR A1590 -5.356 -10.281 7.315 1.00 0.00 H

ATOM 498 H01 THR A1590 -4.355 -9.979 5.867 1.00 10.99 H

ATOM 499 H02 THR A1590 -5.425 -11.383 5.902 1.00 10.99 H

ATOM 500 CA LYS A1591 -2.538 -8.717 4.101 1.00 11.13 C

ATOM 501 C LYS A1591 -2.224 -7.415 4.865 1.00 11.37 C

ATOM 502 O LYS A1591 -1.063 -7.166 5.190 1.00 12.18 O

ATOM 503 HA LYS A1591 -1.713 -9.406 4.301 1.00 0.00 H

ATOM 504 H01 LYS A1591 -2.611 -8.518 3.032 1.00 11.13 H

ATOM 505 H02 LYS A1591 -3.489 -9.141 4.424 1.00 11.13 H

ATOM 506 N THR A1592 -3.264 -6.639 5.194 1.00 11.41 N

ATOM 507 CA THR A1592 -3.153 -5.456 6.046 1.00 11.92 C

ATOM 508 C THR A1592 -2.817 -5.827 7.511 1.00 11.31 C

ATOM 509 O THR A1592 -1.911 -5.229 8.091 1.00 11.64 O

ATOM 510 CB THR A1592 -4.446 -4.600 6.003 1.00 12.04 C

ATOM 511 CG2 THR A1592 -4.370 -3.276 6.778 1.00 13.31 C

ATOM 512 OG1 THR A1592 -4.766 -4.283 4.657 1.00 14.66 O

ATOM 513 H THR A1592 -4.195 -6.895 4.888 1.00 0.00 H

ATOM 514 HA THR A1592 -2.329 -4.846 5.667 1.00 0.00 H

ATOM 515 HB THR A1592 -5.279 -5.178 6.404 1.00 0.00 H

ATOM 516 HG1 THR A1592 -5.019 -5.108 4.203 1.00 0.00 H

ATOM 517 HG21 THR A1592 -5.290 -2.707 6.654 1.00 0.00 H

ATOM 518 HG22 THR A1592 -4.234 -3.439 7.847 1.00 0.00 H

ATOM 519 HG23 THR A1592 -3.546 -2.654 6.428 1.00 0.00 H

ATOM 520 N ALA A1593 -3.490 -6.851 8.059 1.00 11.25 N

ATOM 521 CA ALA A1593 -3.232 -7.390 9.396 1.00 10.28 C

ATOM 522 C ALA A1593 -1.816 -7.979 9.543 1.00 10.71 C

ATOM 523 O ALA A1593 -1.193 -7.797 10.587 1.00 10.58 O

ATOM 524 CB ALA A1593 -4.301 -8.430 9.758 1.00 10.96 C

ATOM 525 H ALA A1593 -4.266 -7.268 7.548 1.00 0.00 H

ATOM 526 HA ALA A1593 -3.324 -6.562 10.100 1.00 0.00 H

ATOM 527 HB1 ALA A1593 -4.177 -8.759 10.790 1.00 0.00 H

ATOM 528 HB2 ALA A1593 -5.304 -8.015 9.671 1.00 0.00 H

ATOM 529 HB3 ALA A1593 -4.245 -9.308 9.117 1.00 0.00 H

ATOM 530 N GLN A1594 -1.301 -8.622 8.486 1.00 10.85 N

ATOM 531 CA GLN A1594 0.082 -9.090 8.410 1.00 11.41 C

ATOM 532 H GLN A1594 -1.900 -8.825 7.690 1.00 0.00 H

ATOM 533 HA GLN A1594 0.245 -9.725 9.283 1.00 0.00 H

ATOM 534 H01 GLN A1594 0.768 -8.243 8.427 1.00 11.41 H

ATOM 535 H02 GLN A1594 0.260 -9.640 7.486 1.00 11.41 H

TER

END
